# High levels of adherence to a rectal microbicide gel and to oral Pre-Exposure Prophylaxis (PrEP) achieved in MTN-017 among men who have sex with men (MSM) and transgender women

**DOI:** 10.1371/journal.pone.0181607

**Published:** 2017-07-27

**Authors:** Alex Carballo-Diéguez, Ivan C. Balán, William Brown, Rebecca Giguere, Curtis Dolezal, Cheng-Shiun Leu, Mark A. Marzinke, Craig W. Hendrix, Jeanna M. Piper, Barbra A. Richardson, Cynthia Grossman, Sherri Johnson, Kailazarid Gomez, Stephanie Horn, Ratiya Pamela Kunjara Na Ayudhya, Karen Patterson, Cindy Jacobson, Linda-Gail Bekker, Suwat Chariyalertsak, Anupong Chitwarakorn, Pedro Gonzales, Timothy H. Holtz, Albert Liu, Kenneth H. Mayer, Carmen Zorrilla, Javier Lama, Ian McGowan, Ross D. Cranston

**Affiliations:** 1 HIV Center for Clinical and Behavioral Studies at New York State Psychiatric Institute and Columbia University, New York, NY, United States of America; 2 Center for AIDS Prevention Studies, Department of Medicine, University of California San Francisco, San Francisco, CA, United States of America; 3 Department of Medicine, Johns Hopkins University, Baltimore, MD, United States of America; 4 Division of AIDS, National Institute of Allergy and Infectious Diseases, Rockville, MD, United States of America; 5 Department of Biostatistics, University of Washington, Seattle, WA, United States of America; 6 National Institute of Mental Health, Bethesda, MD, United States of America; 7 FHI 360, Durham, NC, United States of America; 8 Magee-Womens Research Institute, Pittsburgh, PA, United States of America; 9 Statistical Center for HIV/AIDS Research and Prevention, Seattle, WA, United States of America; 10 Desmond Tutu HIV Centre, University of Cape Town, Cape Town, South Africa; 11 Research Institute for Health Sciences, Chiang Mai University, Chiang Mai, Thailand; 12 Thailand Ministry of Public Health, Nonthaburi, Thailand; 13 Asociación Civil Impacta Salud y Educación, Lima, Peru; 14 Thailand Ministry of Public Health—Centers for Disease Control Collaboration, Bangkok, Thailand; 15 San Francisco Department of Public Health, San Francisco, CA, United States of America; 16 The Fenway Institute, Boston, MA, United States of America; 17 Department of Medicine, University of Puerto Rico, San Juan, PR, United States of America; 18 School of Medicine, University of Pittsburgh, Pittsburgh, PA, United States of America; University of Maryland School of Medicine, UNITED STATES

## Abstract

Trials to assess microbicide safety require strict adherence to prescribed regimens. If adherence is suboptimal, safety cannot be adequately assessed. MTN-017 was a phase 2, randomized sequence, open-label, expanded safety and acceptability crossover study comparing 1) daily oral emtricitabine/tenofovir disoproxil fumarate (FTC/TDF), 2) daily use of reduced-glycerin 1% tenofovir (RG-TFV) gel applied rectally, and 3) RG-TFV gel applied before and after receptive anal intercourse (RAI)—if participants had no RAI in a week, they were asked to use two doses of gel within 24 hours. Product use was assessed by mixed methods including unused product return count, text messaging reports, and qualitative plasma TFV pharmacokinetic (PK) results. Convergence interviews engaged participants in determining the most accurate number of doses used based on product count and text messaging reports. Client-centered adherence counseling was also used. Participants (N = 187) were men who have sex with men and transgender women enrolled in the United States (42%), Thailand (29%), Peru (19%) and South Africa (10%). Mean age was 31.4 years (range 18–64 years). Based on convergence interviews, over an 8-week period, 94% of participants had **≥**80% adherence to daily tablet, 41% having perfect adherence; 83% had **≥**80% adherence to daily gel, 29% having perfect adherence; and 93% had **≥**80% adherence to twice-weekly use during the RAI-associated gel regimen, 75% having perfect adherence and 77% having **≥**80% adherence to gel use before and after RAI. Only 4.4% of all daily product PK results were undetectable and unexpected (TFV concentrations <0.31 ng/mL) given self-reported product use near sampling date. The mixed methods adherence measurement indicated high adherence to product use in all three regimens. Adherence to RAI-associated rectal gel use was as high as adherence to daily oral PrEP. A rectal microbicide gel, if efficacious, could be an alternative for individuals uninterested in daily oral PrEP.

## Introduction

Trials designed to assess the safety and effectiveness of microbicides and other drugs require that participants adhere strictly to prescribed product use regimens. If adherence is suboptimal, safety and effectiveness cannot be adequately assessed. When product use does not occur under direct observation of the researchers, accurate measurement of adherence is frequently a challenge.

In HIV prevention and treatment trials, direct and indirect methods have been used to measure adherence [1]. Direct methods include biological assays of active drug, metabolite or other markers in blood, urine, other bodily fluids or hair to confirm active drug intake. Indirect methods include tablet count, pharmacy refill records, electronic drug monitoring (e.g., MEMS Cap), therapeutic impact (e.g., viral load or CD4 T cell count), clinician assessment, medical chart review and self-reports (quantitative and qualitative).

Unfortunately, all these methods have limitations. Direct methods of adherence assessment, such as pharmacokinetic (PK) testing each sample type collected—plasma, hair, peripheral blood mononuclear cells (PBMC), and dried blood spot–present a trade-off in accuracy of recent dose-taking against averaging dose-taking over long periods, all of which are insensitive to some periods of non-adherence. For example, plasma tenofovir (TFV) and PBMC TFV-diphosphate (TFV-DP) can provide quantitative estimates of doses but only within the previous week, whereas TFV and TFV-DP in hair and dried blood spot respectively provide quantitative measures of adherence averaged over one to several months but lack precision for adherence in the most recent weeks [2–4]. Indirect methods of assessment are even more vulnerable to inaccuracies as tablet or applicator count may be affected, for example, by participants’ forgetting to return the products to the clinic or “dumping” (disposing of products without using them) [5]. Self-reports may be affected by recall bias (a systematic error caused by differences in the accuracy or completeness of participant’s recollections), especially concerning habitual behaviors such as daily pill taking [6]; social desirability (tendency of some individuals to respond in a way they deem to be more socially acceptable than their "true" answer) [7,8]; and misrepresentation.

Given that structured surveys with forced response categories or even semi-structured items may not suffice to understand nuances in product use, qualitative research methods (e.g., in-depth interviews [IDIs]) have been employed to better capture participants’ experience of product use. Yet, while qualitative methods serve exploratory purposes and bring the researchers closer to the “voice” of the participants, the results may not be generalizable.

In sum, currently there is no gold standard for the measurement of adherence to product use in HIV prevention trials. In response to this critical challenge, some researchers have resorted to mixed methods and triangulation (use of two or more measures for a single phenomenon) [4,8]. For example, Pool et al. (2010) used mixed methods and triangulation in a Phase III study of the vaginal microbicide candidate PRO-2000 (MDP301) [9,10]. Data were collected from a random subsample of 725 women using structured case report form (CRF) interviews, coital diaries and IDIs conducted with the participants and their partners. Returned used and unused gel applicators were counted and additional data were collected through focus group discussions and ethnography. Although the authors were able to assess the most likely level of adherence to product use among study participants, they still identified several problems with the measurements used. CRFs–the main source of self-report data on behavior and adherence in many studies–were the least accurate with regard to measuring sexual behavior, product and condom use. Although coital diaries should have been filled in close to the coital event, they could have been completed shortly before having to turn them in, which could have made them susceptible to recall and social desirability biases. IDIs carried out in many different countries required extensive training of personnel, translation of interview guides to different languages, translation of actual interviews back to English, and processing of copious amounts of data. Partners were difficult to recruit, and few came. When discrepant results were reported, male partners deferred to their female partners’ reports (“counting how often you had sex is a ‘woman thing’”). Therefore, the mixed methods approach, although conceptually interesting and recommended by Pool and colleagues, still poses challenges.

This article describes the adherence measurement methods used in MTN-017, their implementation, and the adherence levels to product use observed in the trial. A separate publication will describe the client-centered approach to counseling that was used in MTN-017 to support and encourage good adherence to product use.

## Methods

### Study design and regimens

MTN-017 was reviewed and approved by IRBs at all participating institutions, including the Centers for Disease Control and Prevention, the Thai Ministry of Public Health, the Thai Food and Drug Administration, the University of Cape Town Research Ethics Council, the South African Medicines Control Council, the Chiang Mai University Research Institute for Health Sciences, Chiang Mai University Research Ethics Council, Fenway Community Health, Asociación Civil Impacta Salud y Educación, the Peruvian National Institutes of Health, the Peruvian Food and Drug Administration, the University of Puerto Rico, the University of Pittsburgh, and the University of San Francisco, Committee on Human Research, and the NY State Psychiatric Institute IRB (Protocol #6704), which approved the protocol on December 21, 2012. All participants signed a written informed consent form. The authors confirm that all ongoing and related trials for this drug/intervention are registered.

MTN-017 was a Phase 2, randomized sequence, open-label, expanded safety and acceptability crossover study which enrolled participants between September 2013 and November 2014 and compared 1) daily oral emtricitabine/tenofovir disoproxil fumarate (FTC/TDF); 2) daily use of reduced-glycerin 1% tenofovir (RG-TFV) gel applied rectally; and 3) RG-TFV gel applied before and after receptive anal intercourse (RAI). To assess product use in this study, we decided to build on Pool et al.’s (2010) mixed methods and triangulation approach while enriching it based on our own experience in prior studies of the Microbicide Trials Network (MTN-004, 006, 007, 012) and one independent study (Project Gel, R01 HD059533, Carballo-Diéguez and McGowan, PIs). In these previous studies, we used a variety of assessment methods that included an interactive voice response telephone reporting system [11], computer assisted self-interviews (CASI) [12], remote phone and video IDIs [13], and count of returned used and unused applicators [12]. In addition, in MTN-017 we used telephone short message system (SMS) for adherence reporting as well as also used detectable TFV concentration (cutoff of 0.31 ng/mL) as a qualitative adherence measure provided to the clinical study team within two weeks from sample collection.

**Fig 1:**
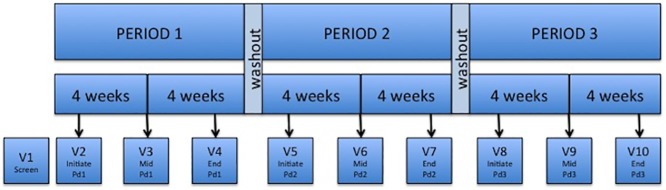
MTN-017 study design. Pd:Period.

The study consisted of three periods with a one-week washout period in between them (Fig 1). Each period lasted eight weeks and included three visits (initiate, mid-, and end-period visits). There was an initial screening visit prior to enrollment/period 1. Follow-up of all enrolled participants was completed in May 2015.

Participants were randomly assigned in equal numbers to use the product regimens in one out of six possible sequences (Table 1). During the daily regimens, participants were instructed to take one tablet orally (daily oral regimen) or to insert one dose of gel rectally per day (daily rectal regimen) at approximately the same time every day. Missed doses were to be taken as soon as possible, unless the next dose was estimated to be due within six hours, in which case, the missed dose was skipped. During the RAI rectal regimen, participants were given instructions to follow the “BAT 24” dosing schedule used in CAPRISA 004 [14], inserting one dose of gel up to 12 hours ***b****efore* RAI, a second dose of gel as soon as possible ***a****fter* RAI but within 12 hours of intercourse, and no more than ***t****wo* doses in 24 hours. Those who did not have RAI in a 7-day period were instructed to insert two doses before the period ended.

**Table 1:** Study regimens in MTN-017.

Sequence	Period 1 (8 weeks)	Washout (~1 week)	Period 2 (8 weeks)	Washout (~1 week)	Period 3 (8 weeks)
1	Daily Oral		Daily Rectal		RAI Rectal
2	RAI Rectal		Daily Oral		Daily Rectal
3	Daily Rectal		RAI Rectal		Daily Oral
4	Daily Rectal		Daily Oral		RAI Rectal
5	Daily Oral		RAI Rectal		Daily Rectal
6	RAI Rectal		Daily Rectal		Daily Oral

FTC: emtricitabine, TDF: tenofovir disoproxil fumarate, TFV: tenofovir; RAI: receptive anal intercourse, RG: reduced glycerin, Daily Rectal: Daily TFV-RG 1% gel, Daily Oral: Daily FTC/TDF, RAI Rectal: RAI-associated TFV-RG 1% gel

## Study sites and participants

The study took place at eight sites in four countries: Cape Town (South Africa); Bangkok and Chiang Mai (Thailand); Lima (Peru); and San Francisco, Pittsburgh, and Boston (US mainland) as well as San Juan, Puerto Rico (US territory). Participants, recruited from a variety of venues including the Internet, primary health clinics and community-based organizations, included male and transgender females aged 18 years or older, non-HIV infected, in general good health, with a history of RAI at least once in the past three months. More details on participant eligibility criteria appear in Cranston RD et al. [15]].

### Adherence measurement and support

**Fig 2:**
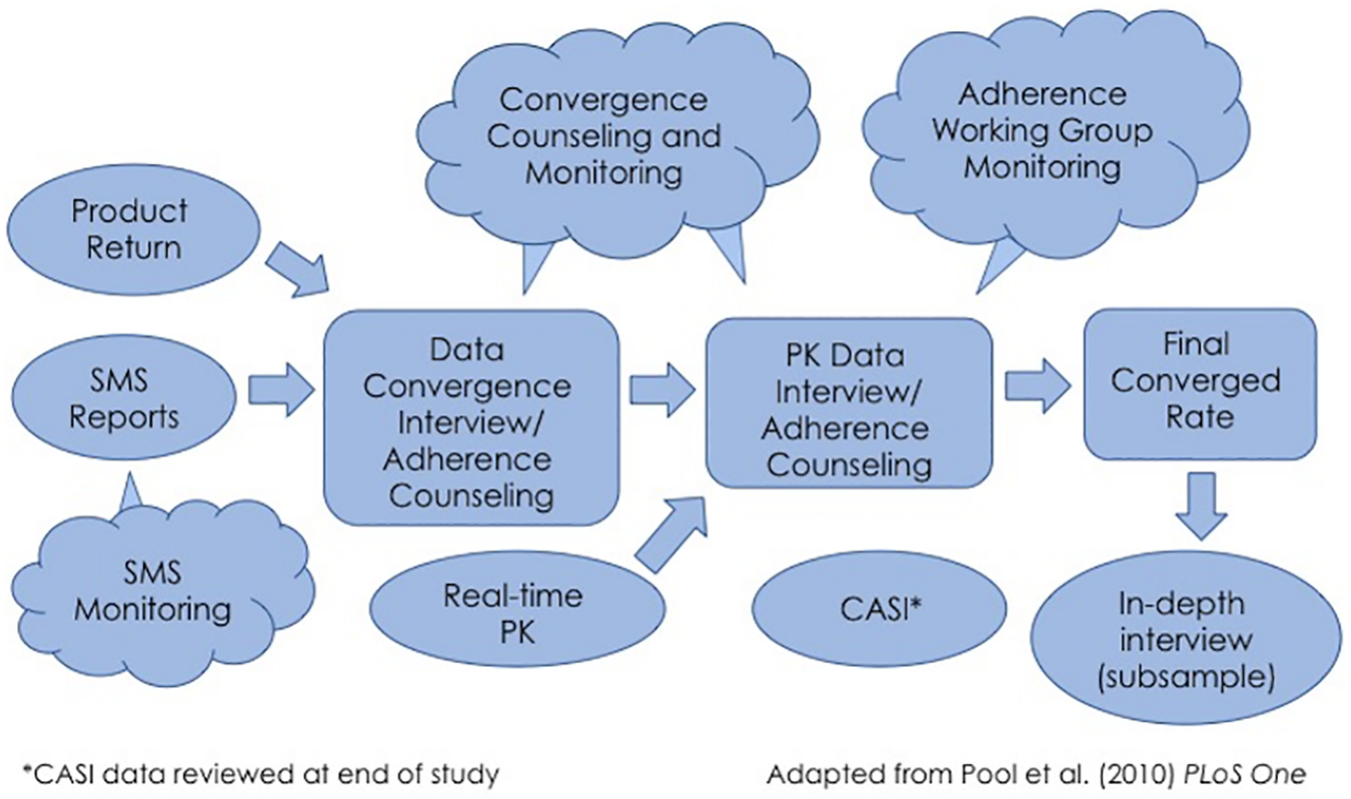
Adherence measurement and counseling in MTN-017. SMS: Short message service, PK: pharmacokinetic results, CASI: Computer Assisted Self-Interview.

Mixed methods were used to estimate adherence to product use during the trial. This is schematically presented in Fig 2.

#### Product return

Participants in daily regimens were given bottles with 30 tablets or bags with 30 applicators at the initiate-period visit to use over a 4-week period and were resupplied at the mid-period visit, which was scheduled to occur 28 days later (see Fig 1). They were asked to bring their bottle with unused tablets and all unused applicators to each mid- and end-period visit. Therefore, for daily regimens, given that participants were expected to use one dose per day, the expected number of doses taken at each follow-up was 28, for a total of 56 per regimen, although it could vary based on the follow-up visit window (the target visit window was between 26 and 30 days). Only those participants who returned for their follow up visit after 30 days would have no product left. Those in the RAI rectal regimen were expected to use at least two doses per week, so the expected minimum number of doses taken at each follow-up was 8, for a total of 16 per regimen, although it could vary based on frequency of sexual activity. Site staff counted the number of unused products returned and entered the data into the Product Dispensation and Return CRF. When participants forgot to bring unused products back to the clinic, they were asked to estimate the number of unused tablets or applicators not returned, and this was entered in a separate field of the CRF. Both returned and unreturned unused doses were summed. That number was subtracted from the number of doses dispensed, and the result was also entered in the CRF as the estimated number of applicators used or tablets taken between visits based on product return.

#### SMS or text message reports

During all three periods, participants received daily text messages at a time of their choice asking them how many times they had used the product *since their last report*. Although we initially considered inquiring through SMS whether RAI had occurred and if product use preceded and followed it, we decided against including these questions to avoid significantly lengthening the daily SMS sessions. Participants received a modest monetary compensation for each completed SMS session, plus a bonus if six out of seven sessions per week were completed. Before each scheduled participant visit to the study site, Columbia University staff prepared individual calendars for each participant depicting the SMS reports received (or missing) for each day during the prior four weeks. Calendars were uploaded to a secure data-sharing site for study staff use during the data convergence interviews, described below. Methods and challenges to implementing the SMS-based reminder and survey system are further detailed in a separate publication [16].

#### Data convergence interview and adherence support

When participants returned to the clinics at each visit they met with an adherence counselor who had been thoroughly trained on client-centered interviewing techniques. The adherence counselor and participant reviewed together the participant’s adherence to product use. The counselor showed the participant a data convergence CRF documenting product return count and SMS report count for the previous four weeks. Using a client-centered approach and following a written script, the counselor first explained to the participants that discrepancies between different measures could be expected, and subsequently engaged the participant’s collaboration to estimate together the most accurate number of times the product had been used *(“Which of these numbers–SMS and returned product count—do you think best represents the actual number of times you used the product*. *Why*?*”)* After discussion with the participant, the counselor entered this number into the CRF along with an explanation of any discrepancy between product return and SMS reports and how the most accurate number was determined in such cases. Cognizant of the objections raised to the use of the term “triangulation” in social research given its connotations of precision [10], we refer to this process as data convergence. These initial questions were followed by client-centered adherence support to reinforce attained adherence and encourage improvement when possible.

#### PK data interviews

At each mid- and end-period visit, plasma samples were collected for PK testing to evaluate detection of study drug using the lower limit of quantitation of the assay (0.31 ng/mL), regardless of study arm [17]. (Note: *Detectable* is not equivalent to *protective* oral drug concentration [18]). Samples were analyzed at a single facility in Baltimore, US. Test results were sent to study sites by the MTN laboratory center within two weeks of sample receipt. At the first end-period visit and in subsequent mid- and end-period visits of the daily regimens, adherence counselors conducted an interview that followed the same script as the data convergence interview but also incorporated a discussion with the participant of whether the PK tests detected or did not detect presence of TFV in their plasma. *(“Last time*, *you reported using the product XX times*. *Now*, *the blood work does/does not show any product in your system*. *Why do you think that might be*?*)*. The PK result and participant’s explanation of the observed results were documented on a PK data convergence CRF. No PK data interviews were conducted during the RAI rectal regimen given the unpredictability of timing of product use in relation to the time of plasma sampling.

#### Determination of final converged rates

Two or three Columbia University behavioral team members independently reviewed the product return CRFs, SMS calendars, and data convergence and PK data convergence CRFs. They determined the most likely adherence score for each participant case by case, and discussed results with each other until reaching consensus. In some cases, adherence determination was straightforward (e.g., product return count and SMS reports coincided, or discrepancies could be explained due to international time differences between the study sites and Columbia University where the SMS calendars were prepared). In other cases, especially when discrepancies were wider, attention was paid to the participants’ explanations and the comments entered by the site adherence counselor, as well as any external circumstances (e.g., problems scheduling interviews exactly at 4-week intervals) that could account for the discrepancies. In no case was an undetectable PK result used to modify an adherence score because there would have been no way to determine a new score beyond what had emerged in the data convergence and PK interviews. However, unexpected negative PK results were carefully recorded, tallied, and discussed during the monthly calls with the Adherence Working Group (discussed below).

#### Computer Assisted Self-Interview (CASI)

At the end of each study period, participants completed a CASI. It included several questions on frequency of RAI, product use on those occasions (before, after, or both before-and-after), and product use during weeks when they did not report RAI (for the RAI rectal regimen).

#### In-depth interviews (IDIs)

Based on the final converged rates, a subset of participants from each country/regimen, half with excellent adherence and half with poor adherence during the first study period (regardless of regimen) were selected for IDIs. Excellent adherence was operationalized as 100% product use during the daily regimens (oral and rectal) or at least 10 occasions of product use during the RAI rectal regimen. Poor adherence was initially operationalized as less than 60% product use during the daily regimens; however, when adherence monitoring showed that very few participants fell under the 60% product use cutoff, the poor adherence criterion was revised to include participants under 80% product use. The script for the IDIs included questions on the experience using the product during the prior eight weeks, challenges encountered, and strategies used to remember to use the product.

#### On-going monitoring

Thorough and continuous monitoring of the adherence measurement and counseling system was in effect throughout the study.

#### SMS monitoring

SMS reporting was methodically controlled daily from the study research offices at Columbia University in New York, US. This involved ongoing data cleaning to eliminate duplicate reports, monitor incomplete sessions, and identify any other anomalies [16]. When participants did not report within 48 hours, the Columbia University staff contacted the site staff to alert them of the situation so that the site staff could contact the participant to follow up on potential problems.

#### Convergence and counseling monitoring

All data convergence and PK interviews as well as adherence support discussions were audio recorded. Native Spanish, Thai and Xhosa-speaking researchers on the Columbia University behavioral team who were also fluent in English followed a standardized fidelity monitoring system. They reviewed the first 10 sessions of each counselor, followed by one of every five for the next 15 sessions, and one of every 10 thereafter.

#### Adherence Working Group monitoring

The study team instituted an Adherence Working Group composed of the protocol chair and co-chair, members of the Columbia University behavioral team, members of FHI360 (an organization that forms part of the MTN Leadership and Operations Center and is responsible for study implementation management and site training), members of SCHARP (the statistical and data management center), the MTN study pharmacist, and NIH staff. The adherence working group met by conference call approximately once a month. In preparation for the call, the Columbia University behavioral team collected information on all cases of participants assigned to daily regimens who had undetectable PK results at mid or end-period visits. This information included the participant’s adherence based on product return count and on SMS reports and calendars, explanations for discrepancies offered to the site adherence counselor during the data convergence and PK interviews as well as relevant information collected during adherence support sessions. Undetectable PK results were categorized as 1,“expected,” (i.e., cases in which the participant had reported interruption of product use seven days prior to PK sample drawing in the oral periods and one day prior to sample drawing in the daily rectal periods) or 2, “unexpected” (e.g., cases in which the participant had reported using the product at least once in the seven days prior to sample drawing in the daily oral period or the day prior to sample drawing for the rectal daily period). This data-review allowed the adherence working group to stay informed of any adherence irregularities that might have required intervention.

### Data management and analysis

Evaluable participants were those who were enrolled and not replaced, or were the final enrolled replacement participant for another participant who was replaced. The MTN-017 study replaced any participants with no adherence data available in either the daily rectal regimen or the RAI rectal regimen (i.e., the participant was not dispensed study product and/or did not report on study product use at least once in the daily rectal regimen, the RAI rectal regimen, or both). This included cases where the adherence data were missing due to loss-to-follow-up, participant refusal to use study product, or site–initiated product holds or discontinuations. These replacement criteria were implemented to ensure enough person-years’ worth of data on the study gel regimens to meet FDA safety requirements in preparation for a Phase III study. In total, eight participants were replaced.

Adherence was calculated as the percentage of prescribed doses taken orally or administered rectally during the visit window for each period, based on the Final Converged Rates entered by the behavioral Columbia University team. For the RAI rectal regimen, the adherence measures used were a) the percentage of doses taken out of an expected 8 doses per 4-week period; b) based on retrospective CASI reports, an occasion of RAI was considered "adherent" only if the gel was used *both* before and after RAI (in accordance with study instructions); we also assessed if the gel was used *either* before *or* after RAI.

Descriptive statistics were generated for all evaluable participants based on their demographic characteristics. We also reported adherence rates by returned product count, SMS messages, data convergence interview, and final converged rate.

The primary adherence scores were based on the final converged rate. We defined participants with high adherence as those with adherence rates ≥ 80%. We employed the Generalized Linear Model (GLM) to compare the proportion of participants with high adherence by regimen. Generalized Estimating Equations (GEE) was used to account for within subject correlation due to repeated measures [19]. The model included regimen and study period as independent variables with logit link function and exchangeable working correlation. We compared daily rectal and RAI rectal to daily oral regimen and reported the adjusted odds ratio (AOR) as well as its corresponding p-value and 95% confidence interval (95% CI).

We calculated the proportion of participants with high adherence by regimen and sequence. To understand whether having used the gel daily prior to using it with RAI could influence high adherence in the RAI rectal regimen, we calculated the proportion of high adherence rates in the sequences in which participants used the gel daily before using it with RAI (i.e., sequences 1, 3, 4 in Table 1) and compared them to the sequences in which they used gel with RAI before using it daily (i.e., sequences 2, 5, 6). Fisher's Exact Test was used for the comparison.

## Results

### Sample description

Participants (N = 187) were recruited from the United States (N = 79, 42%), Thailand (N = 54, 29%), Peru (N = 36, 19%), and South Africa (N = 18, 10%). As shown in Table 2, their mean age was 31.4 years (range 18–64), and on average they had completed 12.3 years of education (SD 1.9). Twelve percent were transgender women (TGW) by self-report. Most identified as gay or homosexual. The three respondents identifying as heterosexual were two TGW and a man. Participants had a median of 2.0 RAI occasions (M = 4.4, SD = 5.7) in the eight weeks prior to baseline.

**Table 2:** Sample characteristics of the evaluable participants in MTN-017 (N = 187).

	Mean (SD)	Range
Age	31.4 (9.3)	18–64
Education (years)	12.3 (1.9)	0–13
	N*	%*
Currently working full- or part-time	144	79%
Currently in school full- or part-time	52	28%
Country of residence		
Peru	36	19%
South Africa	18	10%
Thailand	54	29%
United States (including Puerto Rico)	79	42%
Gender		
Man	163	88%
Transgender Woman	23	12%
Sexual identity		
Gay/homosexual	164	91%
Bisexual	13	7%
Straight/heterosexual	3	2%
	Mean (SD)	Range
Frequency of receptive anal intercourse (past 8 weeks)	4.4 (5.7)	0–40

*Ns may not sum to 187 due to missing data; percentages are of those with non-missing data.

### Adherence to scheduled product use

Table 3 presents the adherence to product use in the different regimens of the trial: daily oral, daily rectal, and RAI rectal (regardless of actual occurrence of RAI) regimens. The table shows the adherence measurements obtained independently from product return (including doses not taken returned to the clinic and, separately, doses reportedly not taken but not returned to the clinic), SMS reports of doses taken, data convergence interview estimates of doses taken, final scores of doses taken after Columbia University behavioral team review, and overall percentage of participants adhering to product use ≥80% of prescribed doses.

**Table 3:** Adherence: Prescribed doses taken orally or administered rectally per regimen in MTN-017.

Regimen	Prescribed dose	Expected number of doses taken^1^	Product return (Means (SD))			SMS	Data convergence interview	Final scores	% of participants with high adherence^2^
			Doses not taken		Doses taken	Doses taken	Doses taken	Doses taken	
			returned	not returned					
Daily oral (FTC/TDF)	1 tablet daily	56 tablets	6.7 (8.4)	0.4 (2.0)	51.1 (8.9)	46.4 (10.7)	51.3 (9.1)	51.1 (8.9)	94
Daily rectal (TFV-RG 1% gel)	1 applicator daily	56 applicators	9.5 (9.6)	0.4 (2.2)	48.0 (11.7)	43.8 (12.8)	48.4 (11.7)	48.0 (11.7)	83
RAI rectal (TFV-RG 1% gel)	At least 2 applicators every 7 days	At least 16 applicators	30.9 (13.5)	0.4 (3.1)	22.9 (12.6)	20.6 (12.0)	22.9 (12.7)	22.9 (12.6)	93

^1^Dependent on timing of the appointments.

^2^Proportion of participants per regimen achieving ≥80% adherence to product use as prescribed.

#### Product return

Based on product return, the estimated mean number of tablets the participants took during the daily oral regimen was 51.1 (SD 8.9). Four percent of participants took all tablets before returning to the clinic and therefore returned none; 88% of participants had tablets left and brought them all to the clinic, for a mean of 6.7 tablets returned; and 8% of participants had tablets left but did not return them to the clinic, the mean number of tablets not returned and not taken for this subgroup being 4.7 (Standard Deviation (SD) 6.1, range 1–24) and for the whole sample, 0.4 (SD 2.0).

The estimated mean number of gel doses the participants used during the daily rectal regimen was 48.0 (SD 11.7). Six percent of participants had used all applicators before returning to the clinic and therefore returned none; 87% had applicators left and returned them all to the clinic, for a mean of 9.5 unused applicators returned; and 7% of participants had some applicators left but did not return them all to the clinic, the mean number of unused and unreturned applicators for this subgroup being 5.2 (SD 6.9, range 1–24) and for the whole sample, 0.4 (SD 2.2).

The mean number of doses taken during the RAI rectal regimen, regardless of occurrence of RAI, was 23.1 (SD 12.5). In this regimen, 2.7% of participants had used all applicators before returning to the clinic and therefore returned none; 90.8% had applicators left and returned them all to the clinic for a mean of 30.9 unused applicators returned; and 6.5% had some applicators left but did not return them all to the clinic, the mean number of unused and unreturned applicators for this subgroup being 5.50 (SD 11.57, range 1–42) and for the whole sample, 0.4 (SD 3.1).

#### SMS

Table 3 also shows the accumulated tally of product use per regimen as reported through SMS from the date of first report of the period until the date of calendar preparation for discussion with participant during the data convergence interview. Doses taken according to SMS reports were 46.4 (SD 10.7) for daily oral, 43.8 (SD 12.8) for daily rectal, and 20.6 (SD 12.0) for RAI rectal. For all regimens, the average number of doses taken based on SMS reports was lower than the numbers of doses taken estimated on the basis of product return. This difference was observed even after sites with poor SMS reporting had been excluded.

#### Data convergence interview

In the data convergence interview, participants leaned towards considering the product return count more accurate than the SMS reports. Several reasons were given for this choice: At times, the calendars did not include the participants’ latest reports (for example, a calendar could have been prepared on a Friday in New York for discussion on a Monday morning in Thailand). Other times, participants had been unable to report on a daily basis plus, at the time of resuming reports, forgotten to provide the cumulative number of doses taken *since their latest report*, which was the question posed in the SMS to allow participants to report for more than one day if they missed a report, mitigating the need to impute missing data from missed SMS reports. However, there were also cases in which participants considered the SMS report more accurate due to having used an applicator incorrectly (e.g., accidentally discharging it before insertion or dropping the applicator on the floor).

#### Final converged adherence rates

The conclusions reached by participants and adherence counselors during the data convergence interview were reviewed by the Columbia University behavioral team following the monitoring procedures described in the methods section. Only slight adjustments were made to ensure that the final rate was not higher than 100% (e.g., if a participant reported using the product 30 times in 29 days), when reported scores were deemed impossible based on further review of product count, or when the team's quality control check identified typographical errors in the number reported, based on the numbers discussed during the audiotaped interview.

#### PK data interviews

**Fig 3:**
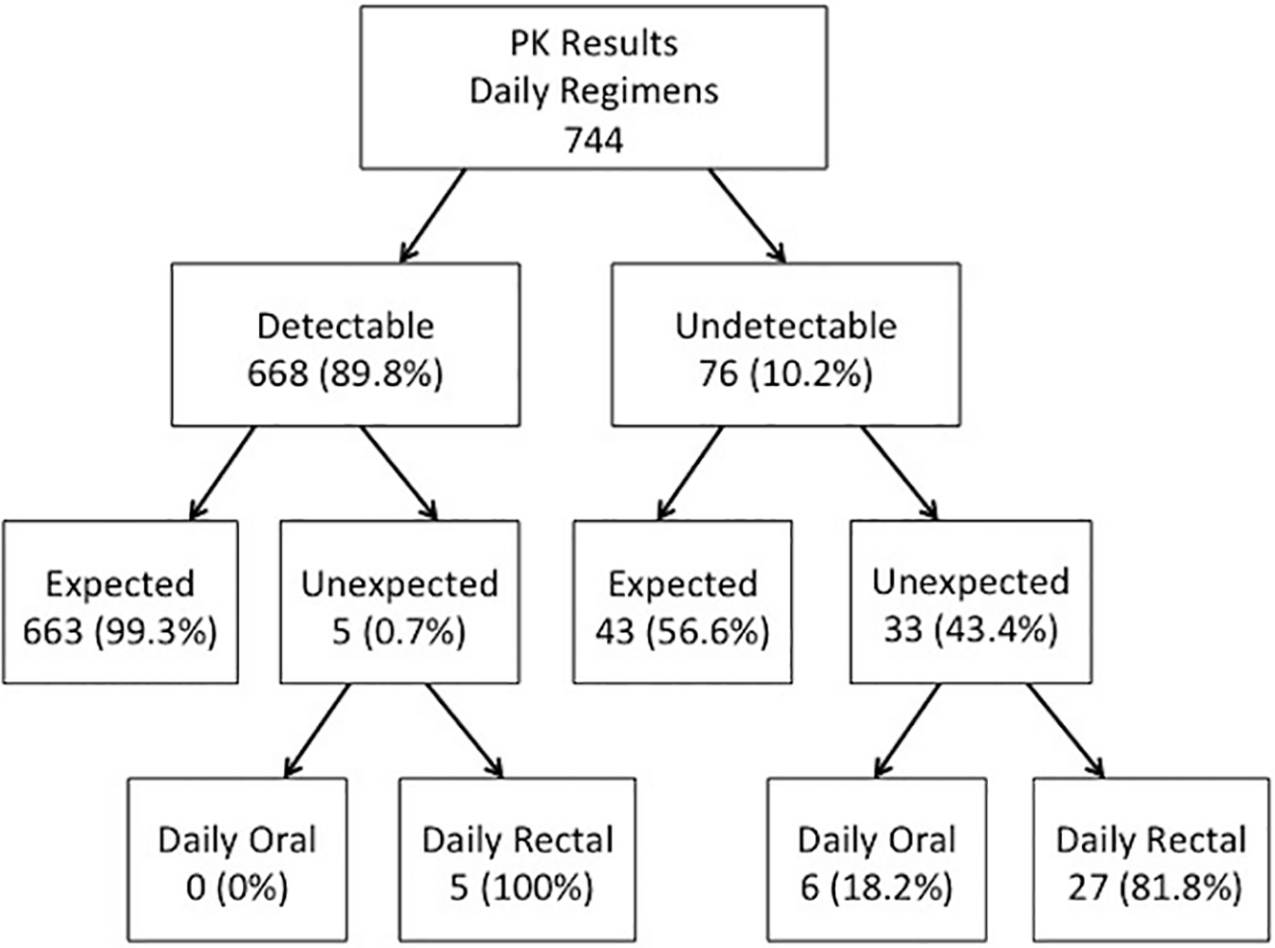
Daily regimens PK results in MTN-017. PK: Pharmacokinetic.

Fig 3 shows the PK results from the daily regimens. Of 744 PK tests performed at mid or end-period visits, 668 (89.8%) showed detectable results and 76 (10.2%) undetectable results. Among the 668 detectable results, all but 5 were expected given the participants’ reports of using the products within seven days prior to plasma sampling in the daily oral period or the day prior to plasma sampling in the daily rectal period. Of the five unexpected detectable results (all in the daily rectal period) with no product use the day prior to plasma sampling, one participant reported being on prescribed oral pre-exposure prophylaxis (PrEP) with FTC/TDF obtained independently from the study. Among the 76 undetectable results (not shown in Fig 3: 11 in the daily oral regimen and 65 in the daily rectal regimen), 43 were expected (e.g., the participant had reported not taking the product either seven days prior to sample collection in the daily oral regimen or the day before sample collection in the daily rectal regimen) and 33 unexpected (undetectable results despite reports of product use in the days prior to sample collection). Of the 33 unexpected undetectable PK results, six were in the daily oral and 27 in the daily rectal regimens. In sum, only 38 of the 744 PK results (5.1%) were unexpected and not aligned with the data convergence interview results, and only 33 of the 744 PK results (4.4%) were negative PK results that did not support data convergence interview reports. Table 4 presents examples of these unexpected negative PK results and the comments the participants made during the interviews. Only three participants, all in the daily gel regimen (two of them in Lima and one in San Francisco) had undetectable PKs at both mid- and end-period visits. Given the types of explanations provided by participants as well as the limitations of PK results to detect drug holidays or precise adherence in the recent weeks, PK results could not be used to modify adherence scores.

**Table 4:** Examples of unexpected undetectable PK results in MTN-017.

Regimen	Visit #	Days between visits	Doses taken	Notes
Daily rectal	3	28	25	SMS Report indicated product was used once per day until the weekend before the visit. Then no product use on 10/18, 10/19, and 10/20 and then product use once per day on 10/21 and 10/22. His visit was on 10/23. When participant was asked why he thought the test result was negative, he stated that after using the product he would often go to the bathroom within 5–15 minutes of inserting it. When this occurred he would notice gel in his stool.
Daily rectal	6	28	28	SMS report indicates daily product use. During PK interview, participant stated that perhaps his PK was not detectable because he was taking medicine for gastritis. He could not think of any other reason why the result would be negative, and reassured the counselor that he had been taking the product.
Daily oral	4	19	19	In the week prior to Visit 4, participant reported using product 7 times, but reported using it 1 time the day before the visit, 3 times 2 days before the visit, and 0 times 3 days before the visit. In PK convergence interview, participant doesn't understand why his result is negative. Reviews calendar carefully with counselor. Says that he took tablet regularly although was not always able to take it at the same time each day. He wonders whether it might have been due to taking the tablet without food.

#### Analysis of final converged adherence rates

An analysis of the final converged rates based on product return, SMS, and DCIs shows that some participants had perfect adherence to their regimens, including 41% in the daily oral regimen, 29% in the daily rectal regimen, and 75% in the RAI rectal regimen (this last figure refers to having used the product at least twice a week, regardless of intercourse). As shown in Table 3, 94% of participants in the daily oral regimen took the tablet ≥ 80% of the prescribed occasions, and 83% in the daily rectal and 93% in the RAI rectal regimens used the gel ≥ 80% of prescribed occasions. In sum, high adherence to product use was observed in all regimens. Yet, it was less likely during the daily rectal regimen (Adjusted Odds Ratio (AOR) = 0.35, 95% CI = 0.19, 0.63, p<0.001) than during the daily oral regimen. Adherence to gel use at least twice weekly during the RAI rectal regimen was similar to that for the daily oral regimen.

Table 5 shows the proportion of participants with high adherence to regimen use by sequence. Comparing those who used the RAI rectal regimen after daily rectal (sequences 1, 3, or 4) to those who used the RAI rectal regimen before daily rectal (sequences 2, 5, or 6) on the proportion reporting high adherence, we found no significant differences (Fisher’s exact tests: 85% vs. 81%, p = 0.56 for daily rectal adherence; 93% vs. 93%, p = 0.99 for RAI rectal regimen adherence).

**Table 5:** Proportion of participants with high adherence to product use by sequence in MTN-017.

	Daily Oral	Daily Rectal	RAI Rectal
Sequence 1: ODR*	100%	87%	93%
Sequence 2: ROD*	90%	79%	90%
Sequence 3: DRO*	90%	87%	97%
Sequence 4: DOR*	90%	81%	90%
Sequence 5: ORD*	97%	78%	97%
Sequence 6: RDO*	94%	87%	90%

*O: daily FTC/TDF tablet; D: daily TFV-RG 1% gel; R: RAI TFV-RG 1% gel

Comparison of RAI after daily rectal (sequences 1, 3 or 4) to RAI before daily rectal (sequences 2, 5, or 6) on daily rectal or RAI rectal was not statistically significant.

### Adherence to product use before and after RAI

Regarding self-reported (CASI) adherence during the RAI rectal regimen, of the 187 evaluable participants, six were missing data for this section of the assessment. The remaining 181 participants reported a median of 4.0 RAI occasions (M = 7.1; SD = 10.1) during the RAI rectal regimen with a median of 2.0 partners (M = 4.5; SD = 7.2). In addition, 23 (12.7%) reported no RAI in the previous eight weeks and 128 (70.7%) reported at least one period of seven days when they did not have intercourse. Of the 152 participants who answered the questions about gel use with RAI, 18 (11.8%) had RAI occasions when they did not use any gel. Most common reasons for not using the gel included forgetting, not having it on hand, and a change in the regular routine.

Adherence to product use before *and* after RAI, as prescribed by study design, was high, with 77% of participants reporting ≥80% adherence. Average adherence for using the gel both before and after RAI was 85.1 (SD: 28.6; 95% CI: 80.5–89.7). Mean adherence for using the gel *before* penetration was 90.5 (SD: 22.6; 95% CI: 86.9–94.2) and 89.5 (SD: 24.3; 95% CI: 85.6–93.4) for using the gel *after* penetration. Average adherence for using the gel *either* before *or* after RAI was 95.0 (SD: 17.5; 95% CI: 92.2–97.8).

## Discussion

High levels of adherence to daily use of oral FTC/TDF, to RG-TFV applied daily, and to RG-TFV applied at least twice per week or before-and-after RAI were achieved in MTN-017. In all regimens, at least 83% of participants used the products on ≥ 80% of the prescribed occasions according to our convergence adherence assessment. Our findings on oral FTC/TDF adherence among MSM and TGW are similar to self-report data from the iPrex study, in which at 8 weeks mean adherence to daily oral FTC/TDF was 93% [20]; however, further analysis found that only 51% of those reporting perfect adherence had drug detected [21], while in this study real-time drug detection enabled us to converge adherence data throughout the study. Our findings on RAI-associated rectal gel use are also similar to those from a previous study on placebo gel use with RAI over a 3-month period, in which participants reported using the gel before RAI on over 80% of occasions [12]. No long-term daily rectal gel studies have been published to compare adherence scores.

The fact that adherence to oral FTC/TDF was high was not surprising given that daily [20] and even intermittent [22] adherence to oral PrEP have shown effectiveness to prevent HIV transmission, and participants in MTN-017 were aware of the effectiveness of daily oral FTC/TDF. This was not the case for a rectal gel, whose potential effectiveness as a microbicide to prevent HIV transmission is still unproven. From this perspective, the high adherence to gel use both in the daily rectal and the RAI rectal regimens was remarkable, showing the strong commitment of the participants to follow trial requirements.

The regimen that showed the lowest adherence rate was daily rectal gel. Although this regimen was selected in order to demonstrate the safety of the gel applied rectally, daily rectal gel application was shown in our study to not be a practical way of delivering prevention of HIV transmission during RAI. Self-administering a rectal gel daily may be cumbersome and less comfortable than taking a daily tablet, especially for individuals who do not have RAI frequently or regularly, as was the case with many of our participants. By contrast, self-administering a gel around the time of RAI could be seen as a practical pre-coital, and even post-coital protective measure, especially for individuals who have infrequent RAI. Interestingly, if the rectal gel could prevent HIV transmission whether applied before *or* after RAI, 95% of the RAI occasions that participants in our sample had would have been protected. Further research is needed to identify the reasons that prevented 11.8% of participants from using the gel during RAI occasions.

From a measurement perspective, it is not possible to have 100% certitude on adherence to product use during the trial, given that dose taking was not directly observed. Following Pool et al. [10], we assume that the converged adherence estimates are the most accurate and plausible on pragmatic grounds, and potential corrections of the converged adherence estimates based on the unexpected undetectable PK results would have had a negligible impact on the final scores. However, we cannot rule out the possibility of deceit in which a participant could have purposely reported product intake with SMS, used only one dose prior to returning to the clinic (which would have resulted in a detectable PK reading) and dumped all the remaining doses (so that product return count would have led us to assume high product use). Yet, the open and collaborative interactions between participants and adherence counselors, which were audio-recorded and monitored, lead us to believe that cases of deceit were nonexistent or very scarce.

This was the first time in a rectal microbicide study that near-real time plasma PK test results were provided to the study team and participant to assist with adherence assessment and counseling. Being able to accomplish this with sites in four different continents and a central laboratory processing specimens in the US was a major accomplishment. Furthermore, obtaining almost 90% detectable and expected PK results in the daily regimens was a significant success for this trial. The fact that 82% of unexpected undetectable results in the daily regimens were observed in the rectal gel regimen may be explained by well-established differences in plasma TFV concentrations that are far higher after oral than rectal dosing [23]. It should also be considered whether behavioral issues (e.g., erroneous administration, urgency to evacuate immediately post gel administration) or physiological ones (e.g., poor absorption) contribute to such results.

The adherence measurement and support systems employed in this trial of less than 200 participants were specifically designed to optimize product use as prescribed to allow the evaluation of product safety. A trial with larger number of participants and different aims (e.g., evaluation of product effectiveness for HIV prevention) would require a different adherence measurement and support design. For example, it would be ideal to simplify the tools used and make measurement less labor intensive. Unfortunately, the design of MTN-017 does not allow us to evaluate separately the relative accuracy of the different tools. At first glance, product return count seems to offer values that are very close to the final adherence scores, and one could be tempted to think that this measure alone suffices. However, participants in our study were aware from the very beginning of the study that product return was just one of the measures of product use, and that they would discuss with an adherence counselor not just product return but also SMS and PK reports.

Furthermore, the use of daily SMS messages may have worked as a reminder to use the product. Also, the collaborative, client-centered approach used for adherence support may have influenced the willingness of participants to adhere as closely as possible to the protocol requirements as well as raised their consciousness on ways to maintain or improve product use. To evaluate these interventions separately would require a different, specifically designed study.

Caution is required to interpret our results. The high rates of product use achieved in the trial do not indicate that comparable high rates would be observed in the “real world” or that product use would reach drug concentrations sufficient to provide protection from HIV. Participants in this trial may have been particularly motivated to fulfill trial requirements, benefited from a highly supportive research environment, knew that they were carefully monitored and agreed to it, and used the products only for 8-week periods. Microbicides used for HIV prevention will require consistent and correct use for as long at the potential of HIV transmission exists, and this may be challenging. Nevertheless, MTN-017 shows the *feasibility* of attaining high levels of adherence and the HIV prevention potential of these technologies.

## Supporting information

S1 Doc MTN-017 study protocol v1.0. (PDF)

S2 Doc MTN-017 adherence dataset. (SAV)
