# Supplementary material for: High levels of adherence to a rectal microbicide gel and to oral Pre-Exposure Prophylaxis (PrEP) achieved in MTN-017 among men who have sex with men (MSM) and transgender women
Source: PLoS One. 2017 Jul 27;12(7):e0181607. doi: 10.1371/journal.pone.0181607 (PMC5531503; doi:10.1371/journal.pone.0181607)
Supplement: S1 Doc — (PDF) [file pone.0181607.s001.pdf]

MTN-017

A Phase 2 Randomized Sequence Open Label Expanded Safety and Acceptability Study of Oral Emtricitabine/Tenofovir Disoproxil Fumarate Tablet and Rectally-Applied Tenofovir Reduced-Glycerin 1% Gel

Microbicide Trials Network

Funding Agencies:
Division of AIDS, US National Institute of Allergy and Infectious Diseases
US National Institute of Mental Health
US National Institutes of Health

Grant #:
UM1 AI 068633

DAIDS Protocol #11857

IND Sponsor:
CONRAD

Pharmaceutical Company Collaborator:
Gilead Sciences, Inc.

IND #73,382

Protocol Chair:
Ross D. Cranston, MD, FRCP

Protocol Co-Chair:
Javier R. Lama, MD, MPH

Version 1.0

July 17, 2012

MTN-017

A Phase 2 Randomized Sequence Open Label Expanded Safety and Acceptability Study of Oral Emtricitabine/Tenofovir Disoproxil Fumarate Tablet and Rectally-Applied Tenofovir Reduced-Glycerin 1% Gel

TABLE OF CONTENTS

LIST OF ABBREVIATIONS AND ACRONYMS vi
PROTOCOL TEAM ROSTER ix
INVESTIGATOR SIGNATURE FORM 1
1 KEY ROLES 6
1.1 Protocol Identification 6
1.2 Sponsor and Monitor Identification 6
1.3 Medical Officer 6
1.4 Clinical Laboratories 7
1.5 Data Center 7
1.6 Study Operations 7
2 INTRODUCTION 8
2.1 Background of Rectal Microbicide Research and Study Rationale 8
2.2 *In vitro* and *Ex vivo* Studies 8
2.3 Animal Studies 9
2.4 Clinical Studies 10
2.5 Behavioral Studies 15
2.6 Rationale for Study Design 15
2.7 Justification of Dosing 17
2.8 Incorporating Effective Antiretroviral HIV-1 Prevention Strategies 17
3 OBJECTIVES 17
3.1 Primary Objectives 17
3.2 Secondary Objectives 18
3.3 Exploratory Objectives 18
4 STUDY DESIGN 19
4.1 Identification of Study Design 19
4.2 Summary of Endpoints 19
4.3 Description of Study Population 20
4.4 Time to Complete Accrual 20
4.5 Study Groups 20
4.6 Sequence and Duration of Trial Period 21
4.7 Expected Duration of Participation 21
4.8 Sites 21
5 STUDY POPULATION 22
5.1 Selection of the Study Population 22
5.2 Inclusion Criteria 22
5.3 Exclusion Criteria 23
5.4 Co-enrollment Guidelines 24

6 STUDY PRODUCT 25
6.1 Regimen....25
6.2 Administration 26
6.3 Formulation 26
6.4 Supply and Accountability 27
6.5 Adherence Counseling 28
6.6 Concomitant Medications and Practices....28
6.7 Prohibited Medications and Practices....29
7 STUDY PROCEDURES 29
7.1 Screening Visit 30
7.2 Study Visits 30
7.3 Follow-Up Phone Calls 32
7.4 Mid-Period Visits 33
7.5 Period End Visits....34
7.6 Follow-up Procedures for Participants Who Discontinue Study Product 35
7.7 Interim Visits....36
7.8 Pharmacokinetics and Pharmacodynamics 37
7.9 Intensive Pharmacokinetics, Pharmacodynamics and Immunological Sample Collection .38
7.10 Behavioral Measures....39
7.11 Clinical Evaluations and Procedures 42
7.12 Laboratory Evaluations....43
7.13 Specimen Collection and Processing 44
7.14 Specimen Handling 44
7.15 Biohazard Containment....44
8 ASSESSMENT OF SAFETY 45
8.1 Safety Monitoring 45
8.2 Clinical Data and Safety Review....45
8.3 Adverse Events Definitions and Reporting Requirements....46
8.4 Expedited Adverse Event Reporting Requirements 47
8.5 Social Harms Reporting....48
8.6 Regulatory Requirements....49
9 CLINICAL MANAGEMENT....49
9.1 Grading System....49
9.2 Dose Modification Instructions....49
9.3 General Criteria for Temporary Hold and/or Permanent Discontinuation of Study Product 49
9.4 Temporary Product Hold/Permanent Discontinuation in Response to Adverse Events.....50
9.5 Criteria for Early Termination of Study Participation 50
9.6 HIV-1 Infection 51
9.7 Hepatitis B Infection 51
9.8 Genital Sexually Transmitted Infection/Reproductive Tract Infection 52
10 STATISTICAL CONSIDERATIONS....52
10.1 Overview and Summary of Design 52

10.2 Study Endpoints 52
10.3 Primary Study Hypotheses 53
10.4 Sample Size and Power Calculations 54
10.5 Participant Accrual, Follow-up and Retention 56
10.6 Randomization 56
10.7 Data and Safety Monitoring and Analysis 57
11 DATA HANDLING AND RECORDKEEPING 58
11.1 Data Management Responsibilities 58
11.2 Source Documents and Access to Source Data/Documents 58
11.3 Quality Control and Quality Assurance 59
12 CLINICAL SITE MONITORING 59
13 HUMAN SUBJECTS PROTECTIONS 60
13.1 Institutional Review Boards/Ethics Committees 60
13.2 Protocol Registration 60
13.3 Study Coordination 60
13.4 Risk Benefit Statement 61
13.5 Informed Consent Process 65
13.6 Participant Confidentiality 65
13.7 Special Populations 66
13.8 Compensation 66
13.9 Communicable Disease Reporting 66
13.10 Access to HIV-related Care 67
13.11 Study Discontinuation 67
14 PUBLICATION POLICY 67
15 APPENDICES 67
APPENDIX I: SCHEDULE OF STUDY VISITS AND EVALUATIONS 68
APPENDIX II: ALGORITHM FOR HIV ANTIBODY FOR SCREENING AND FOLLOW-UP....70
APPENDIX III: SAMPLE INFORMED CONSENT FORM (SCREENING, ENROLLMENT, LONG-TERM STORAGE) 71
References 86

Table of Figures

Table 1: Study Regimen 3
Table 2: Study Groups 21
Table 3: Study Regimen 25
Table 4: Screening 30
Table 5: Initiate Period Visits 31
Table 6: Follow-Up Phone Call 32
Table 7: Mid-Period Visits....33
Table 8: Period End Visits 34
Table 9: Interim Visit(s)....36
Table 10: PK and PD Sample Collection Schedule....37
Table 11: PK/PD/Immunology Sample Collection Schedule 38
Table 12: Minimum Detectable Differences in Rates of Safety Outcomes Assuming 80% Power 54

Table 13: Minimum Detectable Difference in Rates of Acceptability Assuming 80% Power....55
Table 14: Minimum Detectable Difference in Rates of >90% Adherence Assuming 80% Power 56
Table 15: Study Regimen....................57

Figure 1: Study Schedule....................5
Figure 2: Study Schedule....................29
Figure 3: Method for Converging Adherence....................40

MTN-017

A Phase 2 Randomized Sequence Open Label Expanded Safety and Acceptability Study of Oral Emtricitabine/Tenofovir Disoproxil Fumarate Tablet and Rectally-Applied Tenofovir Reduced-Glycerin 1% Gel

LIST OF ABBREVIATIONS AND ACRONYMS

3TC	lamivudine
AE	adverse event
AIDS	Acquired Immunodeficiency Syndrome
AKA	also known as
ALT	alanine transaminase
ART	antiretroviral therapy
ARV	antiretroviral
AST	aspartate aminotransferase
ATN	Adolescent Trials Medicine Network
AUC	area under the curve
BAT	before and after therapy
BID	twice daily
BRWG	Behavioral Research Working Group
BSWG	Biomedical Science Working Group
CAB	Community Advisory Board
CAPRISA	Centre for the AIDS Programme of Research in South Africa
CASI	computer assisted self-interview
CBC	complete blood count
CDC	Centers for Disease Control and Prevention
CFR	Code of Federal Regulations
CHARM	Combination HIV Antiretroviral Rectal Microbicide
CI	Confidence Interval
C_{max}	maximum concentration
CORE	Coordinating and Operations Center
CRF	case report form
CROI	Conference on Retroviruses and Opportunistic Infections
CRS	Clinical Research Site
CT	*Chlamydia trachomatis,* Chlamydia
CTA	Clinical Trial Agreement
CWG	Community Working Group
DAERS	DAIDS Adverse Event Reporting System
DAIDS	Division of AIDS
DMPA	depot medroxyprogesterone acetate
DNA	deoxyribonucleic acid
DSMB	Data Safety Monitoring Board
EAE	expedited adverse event
EC	ethics committees
EFV	efavirenz
FDA	(US) Food and Drug Administration
FHCRC	Fred Hutchinson Cancer Research Center
FTC	emtricitabine
g	grams
GC	*Neisseria gonorrhoeae,* gonorrhea
GCP	Good Clinical Practices
GRFT	griffithsin
HBsAg	hepatitis B surface antigen

HBV	hepatitis B virus
HCV	hepatitis C virus
HEC	hydroxyethylcellulose
HIV	Human Immunodeficiency Virus
HPTN	HIV Prevention Trials Network
HPV	human papillomavirus
HSV-1/2	herpes simplex virus type 1/2
IATA	International Air Transport Association
ICF	Informed consent form
ICH	International Conference on Harmonisation
IDMC	Independent Data Monitoring Committee
IMPACTA	Asociacion Civil Impacta Salud y Educacion
IND	Investigational New Drug
INR	International normalized ratio
IoR	Investigator of Record
IRB	Institutional Review Board
ITT	Intent-to-treat
KOH	potassium hydroxide
kg	kilogram
LDMS	Laboratory Data Management System
LLOQ	lower limit of quantification
µg	microgram
MDP	Microbicides Development Programme
mg	milligram
mL	milliliter
MSM	men who have sex with men
MTN	Microbicide Trials Network
MTT	(3-(4,5-Dimethylthiazol-2-yl)-2,5-diphenyltetrazolium bromide, a tetrazole)
N-9	nonoxynol-9
ng	nanogram
NAAT	nucleic acid amplification test
NDA	new drug application
NIAID	National Institute of Allergy and Infectious Diseases
NIH	National Institutes of Health
NIMH	National Institute of Mental Health
NL	network laboratory
NNRTI	non-nucleoside reverse transcriptase inhibitor
NRTI	nucleoside reverse transcriptase inhibitor
NSAID	non-steroidal anti-inflammatory drugs
NVP	nevirapine
OHRP	Office for Human Research Protections
PAMA	Pediatric, Adolescent & Maternal AIDS
PBMC	peripheral blood mononuclear cell
PBS	phosphate-buffered saline
PCR	polymerase chain reaction
PD	pharmacodynamics
PEP	post-exposure prophylaxis
PK	pharmacokinetics
PMPA	9-R-2-phosphonomethoxypropyl adenine monohydrate
PoR	Pharmacist of Record
PPD	Pharmaceutical Product Development, Inc.
PRO	Protocol Registration Office
PSRT	Protocol Safety Review Team
PSS	polystyrene sulfonate
PTID	participant identification

PT	prothrombin time
PTT	partial thromboplastin time
PUEV	product use end visit
RAI	receptive anal intercourse
RG	reduced-glycerin
RMP	Rectal Microbicide Program
RNA	ribonucleic acid
RPR	rapid plasma reagin
RSC	Regulatory Support Center
RE	Regulatory Entity
RT	reverse transcriptase
RTI	reproductive tract infection
RT-PCR	real-time polymerase chain reaction
RTV	ritonavir
SAE	serious adverse event
SCHARP	Statistical Center for HIV/AIDS Research & Prevention
SDMC	Statistical Data Management Center
SMC	Study Monitoring Committee
SMS	short message service (text message)
SOP	standard operating procedure
SSP	study specific procedures
SUSARs	Suspected, unexpected serious adverse reactions
SSP	study specific procedures
STD	sexually transmitted disease
STI	sexually transmitted infection
TDF	tenofovir disoproxil fumarate
TER	transepithelial resistance
TERIS	Teratogen Information System
TFV	tenofovir
TFV-DP	Tenofovir-diphosphate
UA	urinalysis
ULN	upper limit of normal
UNAIDS	United Nations Programme on HIV/AIDS
UPMC	University of Pittsburgh Medical Center
USA	United States of America
UTI	urinary tract infection
VOICE	Vaginal and Oral Interventions to Control the Epidemic
WHO	World Health Organization
WRHI	Wits Reproductive Health and HIV Institute
w/w	weight/weight
ZDV	zidovudine

MTN-017

A Phase 2 Randomized Sequence Open Label Expanded Safety and Acceptability Study of Oral Emtricitabine/Tenofovir Disoproxil Fumarate Tablet and Rectally-Applied Tenofovir Reduced-Glycerin 1% Gel

PROTOCOL TEAM ROSTER

Protocol Chair:

Ross D. Cranston, MD, FRCP
Protocol Chair
Division of Infectious Diseases - UPMC
Keystone Building, Suite 510
3520 Fifth Ave.
Pittsburgh, PA 15213 USA
Phone: 412-383-2054
Fax: 412-383-2900
Email: rdc27@pitt.edu

Protocol Co-Chair:

Javier R. Lama, MD, MPH
Protocol Co-Chair
Asociacion Civil Impacta Salud y Educacion (IMPACTA)
Av. Almirante Miguel Grau No. 1010.
Lima 4, Peru
Phone: 51-1-206-7800 Ext 407
Fax: 51-1-477-2479
Email: jrlama@impactaperu.org

Site Investigators

Suwat Chariyalertsak, MD, Dr.PH
Site Investigator
Research Institute for Health Sciences
Chiang Mai University
P.O.Box 80 CMU,
Chiang Mai 50202 Thailand
Phone: 66 53 945055
Fax: 66 53 221849
Email: suwat.c@cmu.ac.th

Ross D. Cranston, MD, FRCP
Protocol Chair/Site Investigator
Division of Infectious Diseases-UPMC
Keystone Building, Suite 510
3520 Fifth Ave.
Pittsburgh, PA 15213 USA
Phone: 412-383-2054
Fax: 412-383-2900
Email: rdc27@pitt.edu

Linda-Gail Bekker, MBChB, PhD
Site Investigator
Desmond Tutu HIV Foundation, IIDMM
University of Cape Town
Cape Town, 7705 South Africa
Phone: 27-21-650-6959
Fax: 27-21-650-6963
E-mail: Linda-Gail.Bekker@hiv-research.org.za

Pedro Gonzales, MD, MAS
Site Investigator
IMPACTA
Jr. Putumayo No.177
Lima 4, Peru
Phone: 51-1- 562-1600 Ext 103
Fax: 51-1- 562-1600 Ext 128
Email: pgonzales@impactaperu.org

Timothy H. Holtz, MD, MPH, FACP
Site Investigator
Thailand MOPH - US CDC Collaboration (TUC)
DDC 7 Building, 4th floor
Ministry of Public Health, Soi 4
Nonthaburi 11000 Thailand
Phone: 66-2-580-0669 ext 456
Fax: 66-2-580-0712
Email: tholtz@th.cdc.gov

Albert Liu, MD, MPH
Site Investigator
HIV Prevention Intervention Studies
HIV Research Section, San Francisco
Department of Public Health
25 Van Ness Avenue, Suite 500
San Francisco, CA 94102 USA
Phone: 415-554-9104
Fax: 415-621-0641
Email: albert.liu@sfdph.org

Kenneth Mayer, MD
Site Investigator
The Fenway Institute
Fenway Community Health
1340 Boylston Street
Boston, MA 02115 USA
Phone: 617-927-6400
Fax: 617-267-0764
Email: Kenneth_Mayer@brown.edu

Carmen D. Zorrilla, MD
Site Investigator
University of Puerto Rico- Maternal-Infant Studies Center
Biomedical Studies Building II, First Floor
San Juan, PR 00936
Phone: 787-771-4740
Fax: 787-771-4739
Email: czorrilla@rcm.upr.edu

US National Institutes of Health (NIH)

Roberta Black, PhD
Microbicide Research Branch Chief
National Institute of Allergy and Infectious Diseases (NIAID), Division of AIDS (DAIDS)
6700 B Rockledge Drive
Bethesda, MD 20817 USA
Phone: 301-496-8199
Fax: 301-402-3684
Email: rblack@niaid.nih.gov

Jeanna Piper, MD
DAIDS Medical Officer
NIAID/DAIDS
6700 B Rockledge Drive
Bethesda, MD 20892-7628 USA
Phone: 301-451-2778
Fax: 301-402-3684
Email: piperj@niaid.nih.gov

Cynthia Grossman, PhD
NIMH Program Officer
Center for Mental Health Research on AIDS, National Institute of Mental Health (NIMH)
6001 Executive Blvd, Rm 6201, MSC 9619
Bethesda, MD 20892 USA
Phone: 301-443-8962
Fax: 301-443-9719
Email:grossmanc@mail.nih.gov

MTN CORE

Katherine Bunge, MD
Protocol Safety Physician
Magee-Womens Hospital of UPMC
300 Halket Street
Pittsburgh, PA 15213 USA
Phone: 412-641-3464
Fax: 412-641-1133
Email: kbunge@mail.magee.edu

Beth Galaska Burzuk, MID
Protocol Development Manager
Microbicide Trials Network
204 Craft Avenue
Pittsburgh, PA 15213 USA
Phone: 412-641-5579
Fax: 412-641-6170
Email: galaskaburzukb@upmc.edu

Sharon Hillier, PhD
Principal Investigator
Microbicide Trials Network
204 Craft Avenue
Pittsburgh, PA 15213 USA
Phone: 412-641-8933
Fax: 412-641-6170
Email: shillier@mail.magee.edu

Ken Ho, MD
Protocol Safety Physician
UPMC, Keystone Building, Suite 533
Division of Infectious Diseases
3520 Fifth Avenue
Pittsburgh, PA 15213 USA
Phone: 412-383-7178
Fax: 412-383-2900
Email: hok2@upmc.edu

Cindy Jacobson, PharmD
Director of Pharmacy Affairs
Microbicide Trials Network
204 Craft Avenue
Pittsburgh, PA 15213 USA
Phone: 412-641-8913
Fax: 412-641-6170
Email: cjacobson@mail.magee.edu

Ian McGowan, MD, PhD, FRCP
Co-Principal Investigator
Microbicide Trials Network
204 Craft Avenue
Pittsburgh, PA 15213 USA
Phone: 412-641-8999
Fax: 412-641-6170
Email: imcgowan@pitt.edu

Sharon A. Riddler, MD, MPH
Protocol Physician
UPMC, Keystone Building, Suite 510
3520 Fifth Avenue
Pittsburgh, PA 15213 USA
Phone: 412-383-1741or 412-383-1675
Fax: 412-383-2900
Email: riddler@dom.pitt.edu

Devika Singh MD, MPH
Protocol Safety Physician
Box 359927, Dept. of Global Health, ICRC
325 Ninth Ave.
Seattle WA 98104 USA
Phone: 206-744-8311
Fax: 206-520-3831
Email: dsingh@u.washington.edu

MTN Network Laboratory (NL)

Charlene S. Dezzutti, PhD
Network Laboratory Director
Microbicide Trials Network
204 Craft Avenue
Pittsburgh, PA 15213 USA
Phone: 412-641-3462
Fax: 412-641-6170
Email: dezzuttics@upmc.edu

Craig Hendrix, MD
Pharmacology CORE Principal Investigator
Johns Hopkins University
600 North Wolfe Street, Harvey 502
Baltimore, MD 21287 USA
Phone: 410-955-9707
Fax: 410-955-9708
Email: cwhendrix@jhmi.edu

Florian Hladik, MD, PhD
NL/BSWG Representative
Fred Hutchinson Cancer Research Center
1100 Fairview Avenue North, PO Box 19024, D3-100,
Seattle, WA 98109-1024
Phone: 206-667-6836
Fax: 206-667-4411
Email: fhladik@fhcrc.org

Ratiya Pamela Kunjara Na Ayudhya, MT, ASCP
Laboratory Manager
Microbicide Trials Network
204 Craft Avenue, Room A541
Pittsburgh, PA 15213 USA
Phone: 412-641-6393
Fax: 412-641-6170
Email: pkunjara@mwri.magee.edu

MTN CORE – FHI 360

Kailazarid Gomez, MPM
Senior Clinical Research Manager
FHI 360
PO Box 13950
Research Triangle Park, NC 27709 USA
Phone: 919-544-7040, Ext. 11282
Fax: 919-544-0207
Email: kgomez@fhi360.org

Stephanie Horn
Prevention Research Specialist
FHI 360
PO Box 13950
Research Triangle Park, NC 27709 USA
Phone: 919-544-704,0 Ext. 11274
Fax: 919-544-7261
Email: shorn@fhi360.org

Sherri Johnson, MPH
Clinical Research Manager
FHI 360
1825 Connecticut Avenue, NW
Washington, DC 20009, USA
Phone: 202-884-8677
Fax: 202-884-8844
Email: sjohnson@fhi360.org

Jonathan Lucas, MPH
Community Program Manager
FHI 360
PO Box 13950
Research Triangle Park, NC 27709 USA
Phone: 919-544-7040, Ext. 11458
Fax: 919-544-0207
Email: jlucas@fhi360.org

MTN Statistical Data Management Center (SDMC)

Yevgeny Grigoriev, MD, PhD
SDMC Clinical Affairs Safety Associate
FHCRC—SCHARP
1100 Fairview Avenue North, LE-400
PO Box 19024
Seattle, WA 98109-1024 USA
Phone: 206-667-3440
Fax: 206-667-4812
Email: ygrigori@scharp.org

Marla Husnik, MS
SDMC Statistical Research Associate
FHCRC—SCHARP
1100 Fairview Avenue North, M2-C200
PO Box 19024
Seattle, WA 98109-1024 USA
Phone: 206-667-5633
Fax: 206-667-4812
Email: marla@scharp.org

Karen Patterson, MPH
SDMC Senior Project Manager
Fred Hutchinson Cancer Research Center
(FHCRC)- Statistical Center for HIV/AIDS
Research & Prevention (SCHARP)
1100 Fairview Avenue North, LE-400
PO Box 19024
Seattle, WA 98109-1024 USA
Phone: 206-667-7052
Fax: 206-667-4812
Email: karen@scharp.org

Barbra Richardson, PhD
SDMC Statistician
FHCRC-SCHARP
1100 Fairview Avenue North, LE-400
PO Box 19024
Seattle, WA 98109-1024 USA
Phone: 206-731-2425 (T, W, TH)
Phone: 206-667-7788 (M, F)
Fax: 206-667-4812
Email: barbrar@uw.edu

MTN Behavioral Research Working Group (BRWG)

Ivan Balán, PhD
Behavioral Consultant
HIV Center for Clinical and Behavioral Studies
NYSPI & Columbia University
1051 Riverside Dr., Unit 15
New York, NY 10032 USA
Phone: 212-543-5887
Fax: 212-543-6003
Email: balaniv@nyspi.columbia.edu

Alex Carballo-Diéguez, PhD
Social Science Coordinator and BRWG Representative
HIV Center for Clinical and Behavioral Studies
NYSPI & Columbia University
1051 Riverside Dr., Unit 15
New York, NY 10032 USA
Phone: 212-543-5261
Fax: 212-543-6003
Email: ac72@columbia.edu

Rebecca Giguere, MPH
BRWG Representative
HIV Center for Clinical and Behavioral Studies
NYSPI & Columbia University
1051 Riverside Dr., Unit 15
New York, NY 10032 USA
Phone: 212-543-5829
Fax: 212-543-6003
Email: giguere@nyspi.columbia.edu

Marina Mabragaña, MD
BRWG Representative
HIV Center for Clinical and Behavioral Studies
NYSPI & Columbia University
1051 Riverside Dr., Unit 15
New York, NY 10032 USA
Phone: 212-568-4237
Fax: 212-543-6003
Email: mabraga@nyspi.columbia.edu

MTN Community Working Group (CWG) Representative

Jim Pickett
MTN CWG Representative
200 West Jackson, Suite 2200
Chicago, IL USA 60606
Phone: 313-334-0920
Fax: 312-922-2916
Email: jpickett@aidschicago.org

MTN-017

A Phase 2 Randomized Sequence Open Label Expanded Safety and Acceptability Study of Oral Emtricitabine/Tenofovir Disoproxil Fumarate Tablet and Rectally-Applied Tenofovir Reduced-Glycerin 1% Gel

INVESTIGATOR SIGNATURE FORM

Version 1.0

July 17, 2012

A Study of the Microbicide Trials Network

Funded by:
Division of AIDS, US National Institute of Allergy and Infectious Diseases
US National Institute of Mental Health
US National Institutes of Health

IND Holder:
CONRAD

I, the Investigator of Record, agree to conduct this study in full accordance with the provisions of this protocol. I will comply with all requirements regarding the obligations of investigators as outlined in the Statement of Investigator (Form FDA 1572), which I have also signed. I agree to maintain all study documentation for at least two years following the date of marketing approval for each of the two study products for the indication in which they were studied. If no marketing application is filed, or if the application is not approved, the records will be retained for two years after the investigation is discontinued and the US Food and Drug Administration is notified. Publication of the results of this study will be governed by MTN policies. Any presentation, abstract, or manuscript will be submitted to the MTN Manuscript Review Committee, DAIDS, NIMH, CONRAD, and Gilead Sciences, Inc. for review prior to submission.

I have read and understand the information in the Investigator's Brochure(s), including the potential risks and side effects of the products under investigation, and will ensure that all associates, colleagues, and employees assisting in the conduct of the study are informed about the obligations incurred by their contribution to the study.

____
Name of Investigator of Record

______________________________ ______________________________
Signature of Investigator of Record Date

MTN-017

A Phase 2 Randomized Sequence Open Label Expanded Safety and Acceptability Study of Oral Emtricitabine/Tenofovir Disoproxil Fumarate Tablet and Rectally-Applied Tenofovir Reduced-Glycerin 1% Gel

PROTOCOL SUMMARY

**Short Title:**	Safety and Acceptability Study of Oral Emtricitabine/Tenofovir Disoproxil Fumarate Tablet and Rectally-Applied Tenofovir Reduced-Glycerin 1% Gel
**Clinical Phase:**	Phase 2
**IND Sponsor:**	CONRAD
**Protocol Chair**:	Ross D. Cranston, MD, FRCP
**Protocol Co-Chair:**	Javier R. Lama, MD, MPH
**Sample Size:**	Approximately 186 participants
**Study Population:**	HIV-uninfected males or transgender females who practice receptive anal intercourse (RAI) and who are 18 years of age or older
**Clinical Research Sites:**	Sites selected by the MTN Executive Committee
**Study Design:**	Phase 2, multi-site, randomized, six-sequence, three-period, open label crossover study
**Study Duration:**	Approximately 27 weeks of follow-up per participant with projected 6-9 calendar months of accrual at each site
**Study Regimen:**	Participants will receive emtricitabine (FTC)/tenofovir disoproxil fumarate (TDF) tablet (Truvada®) and tenofovir (TFV) reduced-glycerin (RG) 1% gel

Table 1: Study Regimen

Sequence	**Period 1** (8 weeks)	Washout (~1 week)	**Period 2** (8 weeks)	Washout (~1 week)	**Period 3** (8 weeks)	**Follow-up** (~1 Week)
1	Oral (Daily FTC/TDF)		Rectal (Daily TFV RG 1% gel)		Rectal (RAI-associated TFV RG 1% gel)	
2	Rectal (RAI-associated TFV RG 1% gel)		Oral (Daily FTC/TDF)		Rectal (Daily TFV RG 1% gel)	
3	Rectal (Daily TFV RG 1% gel)		Rectal (RAI-associated TFV RG 1% gel)		Oral (Daily FTC/TDF)	
4	Rectal (Daily TFV RG 1% gel)		Oral (Daily FTC/TDF)		Rectal (RAI-associated TFV RG 1% gel)	
5	Oral (Daily FTC/TDF)		Rectal (RAI-associated TFV RG 1% gel)		Rectal (Daily TFV RG 1% gel)	
6	Rectal (RAI-associated TFV RG 1% gel)		Rectal (Daily TFV RG 1% gel)		Oral (Daily FTC/TDF)	

Primary Objectives

- **Safety:** To compare the safety profiles of daily FTC/TDF tablet, daily TFV RG 1% gel, and RAI-associated TFV RG 1% gel

- **Acceptability:** To evaluate and compare acceptability of daily FTC/TDF tablet, daily TFV RG 1% gel, and RAI-associated TFV RG 1% gel

Primary Endpoints

- **Safety:**
  - Grade 2 or higher adverse events

- **Acceptability:**
  - Participant self-report of ease of use, liking the product, and likelihood of product use if shown to be effective

Secondary Objectives

- **Pharmacokinetics:** To compare systemic and local pharmacokinetics (PK) among daily FTC/TDF tablet, daily TFV RG 1% gel, and RAI-associated TFV RG 1% gel

- **Adherence:** To evaluate and compare adherence to daily FTC/TDF tablet, daily TFV RG 1% gel, and RAI-associated TFV RG 1% gel

Secondary Endpoints

- **Pharmacokinetics:**
    - Tenofovir concentrations in blood plasma, rectal tissue and rectal fluid
    - Tenofovir-diphosphate concentrations in peripheral blood mononuclear cell (PBMC) and rectal tissue
    - Emtricitabine concentrations in blood plasma, rectal tissue and rectal fluid
    - Emtricitabine-triphosphate concentrations in PBMC and rectal tissue

  Note: Rectal tissue will be collected on a subset of participants taking part in the Rectal Biopsy/Fluid Subset

- **Adherence:**
    - Percentage of prescribed doses taken orally or administered rectally in an 8-week period

Exploratory Objectives

- **Pharmacodynamics:** To characterize pharmacodynamic responses following oral and rectal exposure to antiretroviral drugs

- **Mucosal immunity:** To characterize changes in mucosal immunity between baseline and the end of the daily FTC/TDF and TFV RG 1% gel product use

- **Correlation between PK and adherence:** To assess correlation of PK with adherence measures

- **Factors associated with adherence:** To identify factors associated with product adherence and whether they differ by product used (FTC/TDF or TFV RG 1% gel) or regimen (daily use or RAI-associated use)

- **Sexual activity and condom use:** To examine whether sexual activity or condom use varies by product used

- **Product sharing:** To determine the level of sharing of study products with non-participants and to assess with whom products are shared

- **Problem practices:** To determine the prevalence of behavioral practices associated with anal intercourse that may affect microbicide use

Exploratory Endpoints

- **Pharmacodynamics:**
    - Determination of HIV replication in rectal tissue and anti-HIV activity from rectal fluid, at sites with capacity

- **Mucosal immunity:**
    - o Mediators of mucosal immunity at enrollment and at the end of each study period, at sites with capacity

- **Correlation between PK and adherence:**
    - o PK measurements will be compared with adherence measures

- **Factors associated with adherence:**
    - o Identify associations between number of pills and applicators used during the 8-week cycles and acceptability, demographic, and background factors

- **Sexual activity and condom use:**
    - o Participant self-reported frequency of sexual activity and condom use during the trial

- **Product sharing:**
    - o Participant self-reported sharing of study product, quantity of shared study product, and with whom it was shared

- **Problem practices:**
    - o Practices associated with anal intercourse that may affect microbicide use

Figure 1: Study Schedule

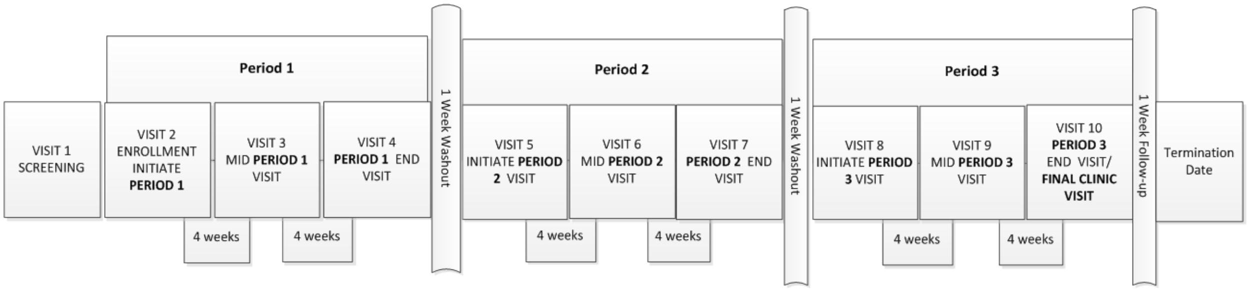

1 KEY ROLES

1.1 Protocol Identification

Protocol Title:	A Phase 2 Randomized Sequence Open Label Expanded Safety and Acceptability Study of Oral Emtricitabine/Tenofovir Disoproxil Fumarate Tablet and Rectally-Applied Tenofovir Reduced-Glycerin 1% Gel
Protocol Number:	MTN-017
Short Title:	Safety and acceptability study of oral FTC/TDF tablet and rectally-applied TFV RG 1% gel
Date:	July 17, 2012

1.2 Sponsor and Monitor Identification

Funding Agencies:	US Division of AIDS (DAIDS)/National Institute of Allergy and Infectious Diseases (NIAID)<br>National Institutes of Health (NIH)<br>6700 B Rockledge Drive<br>Bethesda, MD 20892 USA<br><br>US National Institute of Mental Health (NIMH)<br>6001 Executive Boulevard<br>Rockville, MD 20852 USA
Co-sponsor:	CONRAD<br>1911 North Fort Myer Drive, Suite 900<br>Arlington, VA 22209 USA
Pharmaceutical Company Collaborator:	Gilead Sciences, Inc.<br>333 Lakeside Drive<br>Foster City, CA 94404 USA
Monitor:	Pharmaceutical Product Development (PPD), Inc.<br>929 North Front Street<br>Wilmington, NC 28401-3331 USA

1.3 Medical Officer

Medical Officer:	Jeanna Piper, MD<br>NIAID/DAIDS<br>6700 B Rockledge Drive<br>Bethesda, MD 20892-7628 USA

1.4 Clinical Laboratories

Network Laboratory:	MTN Network Laboratory (NL)<br>204 Craft Avenue<br>Pittsburgh, PA 15213 USA
Pharmacology:	MTN NL Pharmacology CORE<br>Osler 527<br>600 N. Wolfe Street<br>Johns Hopkins University<br>Baltimore, MD 21287 USA

1.5 Data Center

Data Center:	SCHARP<br>FHCRC, 1100 Fairview Avenue N., LE-400,<br>PO Box 19024<br>Seattle, WA 98109-1024 USA

1.6 Study Operations

Study Operations:	FHI 360<br>PO Box 13950<br>Research Triangle Park, NC 27709 USA

2 INTRODUCTION

2.1 Background of Rectal Microbicide Research and Study Rationale

Microbicides are products that are designed to be applied to the vaginal or rectal mucosa with the intent of preventing the acquisition of sexually transmitted infections (STIs) including the human immunodeficiency virus (HIV).[1] While the original impetus for vaginal microbicide development was to provide women with options for HIV prevention in settings where their partners were unwilling to use condoms for penile-vaginal intercourse, there is recognition that rectal microbicides are needed for men and women who practice receptive anal intercourse (RAI).

RAI is associated with the highest probability for sexual acquisition of HIV infection. Unprotected RAI is the sexual behavior with the highest per act risk of HIV acquisition conferring approximately 10 to 20 times more risk than unprotected receptive vaginal intercourse.[2,3] Globally, transgender females and men who have sex with men (MSM) are 19 times more likely to be living with HIV compared with the general population.[4,5]

MTN-017 will evaluate the safety and acceptability of emtricitabine (FTC)/tenofovir disoproxil fumarate (TDF) tablet and rectally-applied tenofovir (TFV) reduced-glycerin (RG) 1% gel in international settings, where HIV prevalence is high, condom use is inconsistent and microbicides have been found to be acceptable among MSM.[6-12]

2.2 *In vitro* and *Ex vivo* Studies

2.2.1 *In vitro* Studies of Emtricitabine (FTC), Tenofovir Disoproxil Fumarate (TDF), and FTC and TDF in Combination

Anti-HIV-1 Activity, Resistance and Mitochondrial Toxicity

Information regarding anti-HIV-1 activity , Resistance and Mitochondrial Toxicity studies can be found in the Investigator's Brochure.[13,14]

2.2.2 *In vitro* and *Ex vivo* Studies of Tenofovir Gel (Various Formulations)

Formulation Testing

A new formulation of tenofovir 1% gel has been developed with a reduced level of glycerin, TFV RG 1% gel. The original formulation, used in all vaginal microbicide tenofovir gel studies, may have been associated with mild gastrointestinal intolerance in a previous rectal microbicide study (RMP-02/MTN-006).[15] These adverse events (AEs) were thought to be potentially linked to the high osmolality of the TFV 1% gel (original vaginal formulation) (3111 mOsm/kg). The TFV RG 1% gel has lower osmolality (846 mOsm/kg) than the TFV 1% gel (original vaginal formulation).[16]

Condom Integrity

Information regarding condom integrity can be found in the Investigator's Brochure.[17]

Safety Testing in Cell Lines

Safety testing of the TFV RG 1% gel epithelial cell lines has demonstrated retention of transepithelial resistance (TER) by Caco-2 and HEC-1-A cell lines, unlike the TFV 1% gel (original vaginal formulation), which induced a transient drop in the epithelial resistance. The TER is the resistance that develops once a cell monolayer grows to confluence. A fall in TER that occurs after product exposure may indicate that the product (e.g. nonoxynol-9 (N-9)) has cellular toxicity. These data suggest the TFV RG 1% gel may be less toxic to the rectal epithelium than the original TFV 1% gel.[16]

Safety Testing in *Ex vivo* Explant Cultures

Preclinical testing of the TFV RG 1% gel and TFV 1% gel was done in colorectal explants. Due to the hyperosmolality of the original TFV 1% gel, colorectal and ectocervical explant tissue exhibited epithelial sloughing and fracture after exposure to the gel. The original TFV 1% gel had no effect on overall tissue viability as measured by MTT (3-(4,5-Dimethylthiazol-2-yl)-2,5-diphenyltetrazolium bromide, a tetrazole). These data suggested that the tenofovir gel did not affect the viability of the tissue, but damaged the epithelium. Subsequent work of the TFV RG 1% gel in colorectal and ectocervical tissue showed no damage to the epithelium by histology and retention of tissue viability as measured by MTT (Dezzutti, data in submission). Additional testing in colorectal and ectocervical explant cultures also showed the TFV RG 1% gel did not compromise product efficacy. Collectively, these data suggest the TFV RG 1% gel is just as effective as the original TFV 1% gel but is safer to the rectal epithelium.

Resistance, and Cross-resistance

HIV-1 isolates with reduced susceptibility to unformulated tenofovir have been selected *in vitro.*[18,19] These viruses expressed a K65R mutation in reverse transcriptase (RT) and showed a 2-4 fold reduction in susceptibility to tenofovir.

Cross-resistance among certain nucleoside reverse transcriptase inhibitors (NRTIs) has been recognized.[13,18] The M184V/I and/or K65R substitutions selected *in vitro* by the combination of FTC and unformulated TDF are also observed in some HIV-1 isolates from subjects failing treatment with TDF in combination with either lamivudine (3TC) or FTC, and either abacavir, didanosine, or zalcitabine.

2.3 Animal Studies

2.3.1 Animal Studies of FTC/TDF in Combination

Information regarding animal studies can be found in the Investigator's Brochure.[14]

2.3.2 TFV 1% Gel (Original Vaginal Formulation)

Information regarding animal studies can be found in the Investigator's Brochure.[17]

2.3.3 TFV RG 1% Gel (Reformulated Reduced-Glycerin)

Rohan and colleagues conducted the first study to evaluate the rectal toxicity of the TFV RG 1% gel.[20] Male and female New Zealand rabbits were administered one of four gels; TFV 1% gel,

0.1% griffithsin (GRFT), TFV 1% gel/0.1% GRFT in combination, and TFV RG 1% gel once a day for 28 consecutive days, at a dose volume of 1 mL. The only microscopic findings judged to be related to study-product involved males administered TFV 1% gel/0.1% GRFT rectal gel and TFV RG 1% gel. Findings in these groups consisted of minimal to mild depletion of secretory material from the mucosal cells and goblet cells of the rectum visible microscopically; however there was no apparent atrophy of the epithelium. This finding was not considered to be adverse. TFV 1% gel, 0.1% GRFT, TFV 1% gel/0.1% GRFT in combination, and TFV RG 1% gel did not result in systemic or local toxicity when administered rectally for 28 consecutive days.

Additional information regarding animal studies of TFV 1% gel (original vaginal formulation) can be found in the Investigator's Brochure.[17]

2.4 Clinical Studies

2.4.1 Clinical Studies of FTC/TDF (Truvada®)

Pharmacokinetics

A pharmacokinetics (PK) study was conducted to establish the bioequivalence of the FTC 200 mg/TDF 300 mg fixed-dose combination tablet relative to administration of FTC capsules and TDF tablets as their individual dosage forms.[14] The steady state PK of FTC and tenofovir were unaffected when FTC and TDF were administered together versus each agent dosed alone.

Truvada® may be administered with or without food.[14] *In vitro* and clinical PK drug-drug interaction studies have shown that the potential for CYP450 mediated interactions involving FTC and tenofovir with other medicinal products is low. FTC and tenofovir are primarily excreted renally by a combination of glomerular filtration and active tubular secretion. No drug-drug interactions due to competition for renal excretion have been observed; however, coadministration of FTC/TDF with drugs eliminated by active tubular secretion may increase concentrations of FTC, tenofovir, and/or the co-administered drug. Drugs that decrease renal function may increase concentrations of FTC and/or tenofovir.

Safety and Efficacy of FTC with TDF

Several studies have assessed the safety and efficacy of Emtriva® (FTC) with Viread® (TDF), albeit none using the fixed dose combination. Four-hundred and forty-seven HIV-1 infected patients have received combination therapy with Emtriva® and Viread® with either a non-nucleoside reverse transcriptase inhibitor (NNRTI) or protease inhibitor for 48 weeks in clinical studies. AEs and laboratory abnormalities observed in clinical trials were generally consistent with those seen in other studies in treatment-experienced or treatment-naïve patients receiving Emtriva® and/or Viread®.

Gilead Study 934 is a Phase 3, randomized, open-label, noninferiority, multicenter study designed to compare a regimen of TDF 300 mg + FTC 200 mg + efavirenz (EFV) QD with a regimen of zidovudine (ZDV) 300 mg/3TC 150 mg BID (as FD Combivir®) + EFV QD in antiretroviral (ARV)-naïve, HIV-1-infected participants.[21] The 48-week data demonstrated that using the time to loss of virologic response as the primary analysis (where missing, switch, or early termination is counted as a failure), the proportion of participants with plasma HIV-1 ribonucleic acid (RNA) levels < 400 copies/mL in an intent-to-treat (ITT) population (n = 487) was 84% in the TDF + FTC group compared with 73% in the ZDV/3TC group (P = 0.002). The

proportion of participants with plasma HIV-1 RNA levels < 50 copies/mL was 80% in the TDF+FTC group versus 70% in the ZDV/3TC group (p = 0.020). Significant differences were also seen between the TDF+FTC and the ZDV/3TC groups in the proportion of participants with increases in CD4+ cell counts (190 and 150 cells/mm^3, respectively; P = 0.002). Safety analysis, based on 511 participants who received any study medication, showed that discontinuation due to AEs occurred more frequently in the ZDV/3TC group (9%) than in the TDF + FTC group (4%) (P = 0.02). The most common AE resulting in discontinuation related to study drug was anemia for the ZDV/3TC group (14/254) and NNRTI-associated rash (2/257) for the TDF+FTC group. Renal safety was similar in the two groups, and no participant discontinued study medication because of renal events. A significantly (P<0.001) greater percentage of participants in the TDF+FTC arm had a lower mean increase from baseline in fasting total cholesterol levels (21 mg/dL) compared with participants in the ZDV/3TC arm (35 mg/dL). At week 48, total limb fat was significantly less in a subset of participants receiving ZDV/3TC (mean of 6.9 kg or 15.2 pounds; n = 49) compared with a subset of participants receiving TDF+FTC (mean 8.9 kg or 19.6 pounds; n = 51; P = 0.03). All participants with confirmed >400 copies/mL of HIV-1 RNA at week 48 or early discontinuation were analyzed for genotypic resistance. Genotype data were limited to 23 participants on ZDV/3TC and 12 participants on TDF+FTC and showed mostly M184V/I (3% in ZDV/3TC participants vs. 1% in TDF + FTC participants) and/or EFV-resistance mutations (7% in ZDV/3TC vs. 4% in TDF + FTC participants), with no participants developing the K65R mutation.

Exacerbations of hepatitis B virus (HBV) have been reported after discontinuation of TDF and FTC. HIV-infected persons co-infected with HBV may have increased values on liver function tests and exacerbation of hepatitis symptoms when TDF or FTC is stopped.[14] Usually symptoms are self-limiting; however, serious complications have been reported. Causal relationship to TDF or FTC discontinuation is unknown. Lactic acidosis and severe hepatomegaly with steatosis, including fatal cases, have been reported with use of nucleoside analogues alone or in combination, including FTC, TDF, and other ARVs.

The iPrEx study was a randomized, double-blind, international, Phase 3 clinical trial, designed to determine whether daily FTC/TDF 200 mg/300 mg tablets was safe and effective for prevention of HIV among MSM and transgender females who have sex with men.[22] Participants were randomized to receive either oral FTC/TDF or placebo. All participants were routinely counseled about safer sex practices, provided condoms and treated for sexually transmitted infections. Grant, et al. reported the safety of FTC/TDF 200 mg/300 mg tablets. No significant differences in the rate of safety endpoints (defined as any adverse event) were reported in the FTC/TDF arm vs. the placebo arm. Two AEs occurred more frequently in the FTC/TDF arm than in the placebo arm, moderate nausea (grade 2 and above), 22 vs. 10 events, p=0.04, and unintentional weight loss, 34 vs. 19 events, P=0.04. Mild elevations of creatinine were also reported, however, of these elevations, 26 (2%) were in the FTC/TDF arm and 15 (1%) were in the placebo group, p=0.08. All elevations resolved either spontaneously or upon study product discontinuation. Twenty-five participants (2%) randomized to FTC/TDF were permanently discontinued from study product. FTC/TDF proved to be safe and well-tolerated.

FTC/TDF is classified as a Food and Drug Administration (FDA) pregnancy Category B drug. Additional general information about FTC/TDF can be found in the most recent Truvada® package insert.[14]

Effectiveness as PrEP

On July 16, 2012, the US FDA approved the use of Truvada, to be taken once daily in combination with safer sex practices, to reduce the risk of sexually acquired HIV-1 infections in adults who are at high risk of becoming infected with HIV-1. Below is a summary of some of the data that was taken into consideration by the US FDA for this indication:

Partners PrEP

Partners PrEP, a study of TDF or FTC/TDF in serodiscordant heterosexual couples in Kenya and Uganda reported high efficacy against HIV acquisition, and the Data and Safety Monitoring Board overseeing the trial recommended stopping the placebo arm early.[23] The team enrolled a total of 4,758 HIV serodiscordant couples. Participants were randomized in a 1:1:1 ratio, to TDF, FTC/TDF, and a matched placebo. Findings from this study revealed 62% (95% CI 44 to 81%, $p<0.0001$) and 75% (95% CI 55 to 87%, $p < 0.0001$) reductions in HIV acquisition compared to those who received placebo in the TDF and FTC/TDF arms, respectively.

iPrEx

The iPrEx study completed with a cumulative follow-up of 3,324 person-years (median, 1.2 years; maximum, 2.8 years per participant), participants in the FTC/TDF group experienced an average of 44% fewer HIV infections than those who were randomized to placebo (95% Confidence Interval [CI], 15 to 63; P=0.005).[16] A total of 100 participants seroconverted while enrolled in the iPrEx study. Of those, 36 HIV infections occurred among the 1,251 participants randomized to receive FTC/TDF compared with 64 HIV infections among the 1,248 participants who were randomized to receive placebo. Oral FTC/TDF was found to be most effective among participants who were adherent to the daily drug regimen. Participants who took the drug on 50% or more days as measured by pill count, self-report, and dispensation records, experienced 50.2% fewer HIV infections (95% CI, 18 to 70; P=0.006). Those who took the drug on 90% or more days presented 73% fewer HIV infections (95% CI, 41 to 88; P=0.001). Overall, efficacy was greatest among participants who, at the time of enrollment, were at highest risk for HIV acquisition (58%), as captured by self-reports of unprotected RAI.

FEM-PrEP

The FEM-PrEP Study was a Phase 3, randomized, placebo-controlled, trial of the effectiveness of daily oral FTC/TDF for HIV prevention among HIV-uninfected women in Kenya, South Africa and Tanzania. The FEM-PrEP study enrolled HIV-negative women between the ages of 18 and 35 who were at higher risk for HIV. Higher risk was defined as: 1) has had at least one vaginal sex act in the last two weeks, or 2) has had more than one sexual partner in the last month. All participants in the study were provided comprehensive HIV prevention services, including male and female condoms, intensive risk-reduction behavioral counseling, and testing and treatment for sexually transmitted infections. This trial was stopped early for futility by the Independent Data Monitoring Committee on April 7, 2011 at which point the study had accumulated 56 endpoints, with 28 endpoints in the FTC/TDF arm and 28 endpoints in the placebo arm.[24] No significant safety concerns were noted. Final study results are pending.

2.4.2 TFV 1% Gel (Original Vaginal Formulation)

Pharmacokinetics

Rectal Administration

In a Phase 1 study, RMP-02/MTN-006, 12 men and women participants received sequentially: single-dose oral TDF, single-dose TFV 1% gel (original vaginal formulation) per rectum, 7 daily doses of TFV 1% gel (original vaginal formulation) per rectum. Blood, tissue, and luminal sampling followed each of these 3 dosing periods. At 30 minutes following single rectal, topical dosing, rectal tissue concentrations were 100-times greater than 30 minutes after single oral dosing. Multiple rectal doses resulted in five times greater concentrations in tissue when compared to a single rectal dose with no significant increase in plasma concentrations. Peripheral blood mononuclear cell (PBMC) tenofovir-diphosphate (TFV-DP) concentrations were below limits of quantitation in the vast majority of specimens collected in the 24 hours following a single rectal dose.[15]

Safety

Penile Administration

In a male tolerance study (CONRAD A04-099/IND 73,382),[25] TFV 1% gel was well-tolerated in men following seven days of once-daily exposure, for 6 to 10 hours, to the penis. There were few reported and observed genital findings after product use including mild pain (burning, irritation, discomfort) and pruritus. All observed findings were classified as mild, small in number and requiring no treatment. Reported symptoms were mild, of short duration and resolved by the final visit. There were no noticeable differences between signs and symptoms of genital irritation in the circumcised compared to uncircumcised group.

Rectal Administration

RMP-02/MTN-006 participants took a single oral dose of tenofovir disoproxil fumarate and applied a single rectal dose of TFV 1% gel (original vaginal formulation) or placebo gel before applying the same gel again for 7 days. This study made comparisons between the vaginally-formulated original TFV 1% gel and a single dose oral tenofovir tablet in 18 HIV-seronegative participants randomized 2:1 to active product or placebo. Single-dose topical exposure of the TFV 1% gel showed no increase in AEs compared with placebo. After 7-days of exposure to TFV 1% gel, a total of 53 adverse events were reported with all 12 participants reporting at least one event [41 Grade 1 AEs were reported in 12 participants, 6 Grade 2 AEs in 3 participants, and 6 Grade 3 AEs in 2 participants]. The majority of these were gastrointestinal. Conversely, there were significantly fewer AEs reported in the 6 participants randomized to placebo (HEC) [8 Grade 1 AEs in 4 participants and 1 Grade 2 AE in 1 participant] were reported. These findings support the use of a reduced-glycerin formulation in future rectally-applied tenofovir gel clinical trials.[15]

Effectiveness for Prevention of HIV

CAPRISA 004

The CAPRISA 004 trial was a Phase 2B trial that was designed to assess the effectiveness and safety of a TFV 1% gel, for the prevention of HIV acquisition in women.[26] A double-blind, randomized controlled trial was conducted comparing tenofovir gel (*n* = 445) with placebo gel (*n* = 444) when used in a pericoital regimen, in sexually active, HIV-uninfected 18 to 40 year-old women in urban and rural KwaZulu-Natal, South Africa HIV serostatus, safety, sexual behavior and gel and condom use were assessed at monthly follow-up visits for 30 months. HIV incidence in the tenofovir gel arm was 5.6 per 100 women-years, compared to 9.1 per 100 women-years in the placebo gel arm (incidence rate ratio = 0.61; *P*=0.017). Tenofovir gel reduced HIV acquisition by an estimated 39% overall, and by 54% in women with high gel adherence. No increase in the overall adverse event rates was observed.

However, the daily dosing regimen of TFV 1% gel (original vaginal formulation) used in the ongoing MTN-003 (VOICE) Phase 2B effectiveness study was not shown to be associated with reduced rates of HIV acquisition and the VOICE Data Safety Monitoring Board (DSMB) recommended that this arm of the VOICE study be stopped for futility.[27]

2.4.3 TFV RG 1% Gel

Safety

Rectal Administration

MTN-007 was designed to assess the safety, adherence, and acceptability of the TFV RG 1% gel. Sixty-five participants (45 men and 20 women) aged 18-61 were recruited from three US sites; Pittsburgh (PA), Birmingham (AL), and Boston (MA). Participants were randomized 1:1:1:1 to receive TFV RG 1% gel, HEC placebo gel (HEC), 2% N-9, or a no treatment arm (No Rx). The N-9 gel arm was included as a positive control for the mucosal safety assays. Participants were evaluated at Baseline, after a single dose, and after 7 daily doses of study product. Systemic and mucosal safety, acceptability, and adherence were evaluated at all three visits. Comprehensive mucosal safety evaluation included histology, fecal calprotectin, epithelial sloughing, cytokine expression (mRNA and protein), flow cytometry of mucosal T cell phenotype, and rectal microflora. All mucosal assays were performed on biopsies collected at 9 and 15 cm from the anal margin. Acceptability and adherence were determined by computer-administered questionnaires and interactive telephone response. Product adherence was ≥ 94% when measured with case report forms (CRFs). AEs were generally mild (N=121 (80%)) or moderate (N=27 (18%)). Two Grade 3 and one Grade 4 events occurred in the no treatment arm or before product use. There was no significant difference in the prevalence of AEs across the four arms of the study, further no participant discontinued study product due to an AE. Based on the MTN-007 data, it appears the TFV RG 1% gel was well tolerated and should be advanced to Phase 2 rectal microbicide development in MTN-017.[28]

2.5 Behavioral Studies

2.5.1 Adherence of FTC/TDF Tablet as Pre-Exposure Prophylaxis Among MSM and Transgender Females Who Have Anal Sex

In the iPrEx study 2,499 sexually active MSM received a daily oral dose of FTC/TDF fixed dose combination or a placebo pill. The study found participants with greater adherence to FTC/TDF tablet were less likely to become infected with HIV. Adherence measures included pill counts, self-report through computer-assisted self-interview (CASI), interviewer-administered interviews, and measurements of drug concentrations in participants' blood and hair. Median adherence based on pill count was 95%. Nevertheless, blood drug testing indicated that half of the participants had no detectable drug in their blood. The findings from the iPrEx study underscore the importance of adherence in microbicide trials and the need for improved assessment measures (iPrEx Fact Sheet: Adherence).

2.5.2 Acceptability of Rectal Use of TFV 1% Gel (Original Vaginal Formulation) and TFV RG 1% gel

RMP-02/MTN-006 assessed acceptability of the candidate microbicide TFV 1% gel (original vaginal formulation) and HEC placebo gel among 18 men and women who engage in receptive anal intercourse. Participants were randomly assigned to use one of the gels once per day for 7 days. Only 25% of participants in the tenofovir arm versus 50% in the control arm reported liking the gel very much. In addition, 42% of participants in the tenofovir group vs. 17% in the control group experienced symptoms or discomforts. Nevertheless, similar proportions in both groups (75% in tenofovir arm vs. 67% in control arm) indicated they would be very likely to use the gel in the future if it were found to be protective against HIV. None of these differences reached statistical significance. Nevertheless, likelihood to use in the future, despite small proportion of participants liking tenofovir and many experiencing symptoms or discomfort when using it, may indicate that concerns about HIV transmission could lead participants to tolerate discomfort but still want to use a microbicide gel that offered some protection against HIV. Microbicide acceptability is a multifactorial construct, and this small, diverse sample hinders definitive conclusion of acceptability. Yet, the acceptability evaluation of RMP-02/MTN-006 highlighted potential acceptability problems that need to be studied with larger samples.

MTN-007 evaluated TFV RG 1% gel as compared to a placebo and nonoxynol-9 gel among women and men who have anal sex. Product adherence was ≥ 94% based on used and unused applicator counts, and self-report via daily phone reporting system indicated mean adherence to gel use was 90.7% (standard deviation=19.1%). Participant's self-reported likelihood of future product use (acceptability) as reported via Web CASI was 86.7% (tenofovir 1% gel), 93.3% (HEC), and 62.5% (N-9).[28]

2.6 Rationale for Study Design

The tenofovir-emtricitabine combination pill has an established safety and efficacy profile when used as part of combined antiretroviral therapy to treat HIV infection. More recently it has demonstrated efficacy to prevent HIV infection when taken daily in the iPrEX study of at risk MSM and transgender females, again with a benign safety profile. Although this finding is promising, the success of HIV prevention strategies will ultimately depend on the availability of

different products and methods of administration that may be used according to individual preference. Consequently, alternatives to an oral medication need to be sought.

As most receptive anal intercourse is facilitated by the use of a lubricant, the use of a rectal gel microbicide has the potential advantage of both familiarity and context. The MTN-007 Phase 1 evaluation of rectal RG TFV 1% gel given for up to seven days demonstrated a good safety and acceptability profile.[29] This candidate rectal microbicide now needs to be evaluated with more prolonged dosing in the population for which it will ultimately be intended to ensure its safety and acceptability prior to consideration for inclusion in a rectal microbicide effectiveness study.

MTN 017 is designed to compare two products type; a pill and a rectal gel. The crossover design of MTN-017 will compare the treatments (daily FTC/TDF tablet, daily RG TFV 1% gel, and RAI-associated RG TFV 1% gel) in the same participants after 8 weeks of product use, while randomization of product sequence will ensure an unbiased estimate of the treatment effects including safety (See Section 8, Assessment of Safety) and acceptability. Further, the protocol includes a 7 day washout between regimen use periods, the prolonged intracellular half-lives of FTC-TP and TFV-DP, with half-lives of 40 and 60 hours, respectively, will allow a decay to ~6% and ~15% of peak concentrations.[30-32]

Mucosal Immunology and Pharmacodynamics

Recent Phase 1 rectal safety studies of tenofovir 1% gel (RMP-02/MTN-006 and MTN-007) have generated important data on the mucosal safety and *ex vivo* antiviral efficacy of the product following seven daily applications of the gel. The RMP-02/MTN-006 study demonstrated that use of the original tenofovir 1% gel did not induce significant mucosal inflammation but was associated with significant antiviral efficacy in the *ex vivo* explant challenge model.[15] The MTN-007 study demonstrated that the RG tenofovir 1% gel was also safe and did not appear to be associated with mucosal inflammation although there was some evidence of alterations in gene expression using microarray analysis of mucosal samples.[29]

It is important that mucosal safety and antiviral efficacy are evaluated following longer periods of dosing with tenofovir 1% gel. Consequently, we propose to undertake an assessment of mucosal safety and *ex vivo* explant infection in a subset of MTN-017 participants. This assessment will include histology, flow cytometry of mucosal mononuclear cells, and microarray assessment of gene expression. These evaluations will be conducted at baseline as well as after each dosing phase. It is hoped that these data will demonstrate whether extended dosing (8 weeks rather than 1 week) of study product is associated with mucosal changes above and beyond those seen in MTN-007.

2.7 Justification of Dosing

FTC/TDF Tablets
Choice of the FTC/TDF tablet strength is based on the available strength of Truvada®, a US Food and Drug Administration (FDA) approved medication with the indications of treatment and prevention[33] of HIV-1 infection. This once-daily film-coated tablet contains 200 mg of FTC and 300 mg of TDF, which is equivalent to 245 mg of tenofovir disoproxil, as active ingredients. Dosages used in MTN-017 are the same as licensed doses and the safety profile has been assessed as part of FDA licensure.

TFV RG 1% Gel Daily Dosing
Choice of the tenofovir 1% gel concentration is based on both animal and clinical evidence suggesting an appropriate safety profile and potency.

TFV RG 1% Gel RAI-Associated Dosing
The pericoital dosing regimen used in the CAPRISA 004 study was associated with a significant reduction in HIV acquisition.[34]

Intermittent dosing of rectal tenofovir gel associated with sexual activity may be a more feasible strategy for long-term usage. Data are needed on the safety, acceptability, and PK of this dosing schedule in at-risk men.

2.8 Incorporating Effective Antiretroviral HIV-1 Prevention Strategies

The MTN-017 Protocol Team will follow all relevant national policies regarding HIV-1 prevention. Locally approved effective agents will be incorporated into the HIV prevention package and presented to participants. See MTN-017 Study-Specific Procedures (SSP) Manual available at www.mtnstopshiv.org, for additional guidance.

3 OBJECTIVES

3.1 Primary Objectives

- **Safety:** To compare the safety profiles of daily FTC/TDF tablet, daily TFV RG 1% gel, and RAI-associated TFV RG 1% gel
- **Acceptability:** To evaluate and compare acceptability of daily FTC/TDF tablet, daily TFV RG 1% gel, and RAI-associated TFV RG 1% gel

3.2 Secondary Objectives

- **Pharmacokinetics:** To compare systemic and local PK among daily FTC/TDF tablet, daily TFV RG 1% gel, and RAI-associated TFV RG 1% gel

- **Adherence:** To evaluate and compare adherence to daily FTC/TDF tablet, daily TFV RG 1% gel, and RAI-associated TFV RG 1% gel

3.3 Exploratory Objectives

- **Pharmacodynamics:** To characterize pharmacodynamic responses following oral and rectal exposure to antiretroviral drugs

- **Mucosal immunity:** To characterize changes in mucosal immunity between baseline and the end of the daily FTC/TDF and TFV RG 1% gel product use

- **Correlation between PK and adherence:** To assess correlation of PK with adherence measures

- **Factors associated with adherence:** To identify factors associated with product adherence and whether they differ by product used (FTC/TDF or TFV RG 1% gel) or regimen (daily use or RAI-associated use)

- **Sexual activity and condom use:** To examine whether sexual activity or condom use varies by product used

- **Product sharing:** To determine the level of sharing of study products with non-participants and to assess with whom products are shared

- **Problem practices:** To determine the prevalence of behavioral practices associated with anal intercourse that may affect microbicide use

4 STUDY DESIGN

4.1 Identification of Study Design

MTN-017 is a Phase 2, multi-site, randomized, six-sequence, three-period, open-label crossover study.

4.2 Summary of Endpoints

Primary Endpoints

- **Safety:**
    - Grade 2 or higher adverse events

- **Acceptability:**
    - Participant self-report of ease of use, liking the product, and likelihood of product use if shown to be effective

Secondary Endpoints

- **Pharmacokinetics**:
    - Tenofovir concentrations in blood plasma, rectal tissue and rectal fluid
    - Tenofovir-diphosphate concentrations in PBMC and rectal tissue
    - Emtricitabine concentrations in blood plasma, rectal tissue and rectal fluid
    - Emtricitabine-triphosphate concentrations in PBMC and rectal tissue

    Note: Rectal tissue will be collected on a subset of participants taking part in the Rectal Biopsy/Fluid Subset

- **Adherence:**
    - Percentage of prescribed doses taken orally or administered rectally in an 8-week period

Exploratory Endpoints

- **Pharmacodynamics**:
    - Determination of HIV replication in rectal tissue and anti-HIV activity from rectal fluid, at sites with capacity

- **Mucosal Immunity**:
    - Mediators of mucosal immunity at enrollment and at the end of each study period, at sites with capacity

- **Correlation between PK and adherence**:
    - PK measurements will be compared with adherence measures

- **Factors associated with product adherence:**
    - o Identify associations between number of pills and applicators used during the 8-week cycles and acceptability, demographic, and background factors

- **Sexual activity and condom use:**
    - o Participant self-reported frequency of sexual activity and condom use during the trial

- **Product sharing:**
    - o Participant self-reported sharing of study product, quantity of shared study product, and with whom it was shared

- **Problem practices:**
    - o Practices associated with anal intercourse that may affect microbicide use

4.3 Description of Study Population

The study population will be HIV-uninfected males or transgender females who are 18 years of age or older who practice receptive anal intercourse.

4.4 Time to Complete Accrual

Accrual is expected to be completed in approximately 6-9 months per site.

4.5 Study Groups

Approximately 186 individuals will be randomized equally across all 6 sequences. All study participants will complete each study period (oral, rectal, RAI-associated) once in a randomly assigned order.

Table 2: Study Groups

Sequence	**Period 1** (8 weeks)	Washout (~1 week)	**Period 2** (8 weeks)	Washout (~1 week)	**Period 3** (8 weeks)	**Follow-up** (~1 Week)
1	Oral (Daily FTC/TDF)		Rectal (Daily TFV RG 1% gel)		Rectal (RAI-associated TFV RG 1% gel)	
2	Rectal (RAI-associated TFV RG 1% gel)		Oral (Daily FTC/TDF)		Rectal (Daily TFV RG 1% gel)	
3	Rectal (Daily TFV RG 1% gel)		Rectal (RAI-associated TFV RG 1% gel)		Oral (Daily FTC/TDF)	
4	Rectal (Daily TFV RG 1% gel)		Oral (Daily FTC/TDF)		Rectal (RAI-associated TFV RG 1% gel)	
5	Oral (Daily FTC/TDF)		Rectal (RAI-associated TFV RG 1% gel)		Rectal (Daily TFV RG 1% gel)	
6	Rectal (RAI-associated TFV RG 1% gel)		Rectal (Daily TFV RG 1% gel)		Oral (Daily FTC/TDF)	

4.6 Sequence and Duration of Trial Period

The total duration of participation from the Enrollment Visit to the Termination Date is anticipated to be 27 weeks; this includes three eight-week study product use periods and two one-week washout periods plus a one-week follow-up period after the Period 3 End Visit. All participants reporting an AE will be followed clinically until the AE resolves (returns to baseline) or stabilizes. Visits may be completed within specified windows around target dates. Detailed information regarding visit windows will be thoroughly described in the MTN-017 SSP Manual.

4.7 Expected Duration of Participation

The expected duration of scheduled follow-up is approximately 27 weeks per participant.

4.8 Sites

Sites approved by the MTN Executive Committee will participate in MTN-017.

5 STUDY POPULATION

5.1 Selection of the Study Population

The inclusion and exclusion criteria in Sections 5.2 and 5.3 will be utilized to ensure the appropriate selection of study participants.

5.1.1 Recruitment

Participants will be recruited from a variety of venues including, primary care health clinics and community-based locations. Participants also will be referred to the study from other local research projects and health and social service providers serving the target study population. Recruitment materials will be approved by site Institutional Review Boards/Ethics Committees (IRBs/ECs) prior to use per local and International Conference on Harmonisation (ICH)/Good Clinical Practices (GCP) requirements. Site community representatives should advise on these materials before they are submitted to the IRB/EC for review.

5.1.2 Retention

Once a participant is enrolled and randomized in MTN-017, the study site will make every effort to retain the participant in follow-up to minimize possible bias associated with loss-to-follow-up. A retention rate of 95% will be targeted at each site. Each study site will be responsible for developing and implementing local Standard Operating Procedures (SOPs) to ensure and target high rates of retention.

5.2 Inclusion Criteria

Participants must meet all of the following criteria to be eligible for inclusion in the study:

1. Male or transgender female $\geq$ age of 18 at Screening, verified per site SOP

2. Able and willing to provide written informed consent

3. HIV-1 uninfected at Screening and Enrollment, per applicable algorithm in Appendix II

4. Able and willing to provide adequate locator information, as defined in site SOP

5. Available to return for all study visits, barring unforeseen circumstances and willing to comply with study participation requirements

6. In general good health at Screening and Enrollment, as determined by the site IoR or designee

7. Per participant report, a history of consensual RAI at least once in the past 3 months

8. Per participant report at Screening and Enrollment, agrees not to engage in receptive or insertive sexual activity with another study participant for the duration of study participation.

9. Willing to use study-provided condoms for the duration of the study for penetrative intercourse

10. Willing to not take part in other research studies involving drugs, medical devices, vaccines or genital products for the duration of study participation (including the time between Screening and Enrollment)

11. Men and transgender females who agree to take part in the PK, PD and Mucosal Immunology Subset, must also agree to abstain from:

    a. Inserting anything into the rectum, including abstaining from RAI for 72 hours after the collection of biopsies

    b. Taking non-steroidal anti-inflammatory drugs (NSAIDs), aspirin and/or other drugs that are associated with increased likelihood of bleeding following mucosal biopsy collection for 72 hours prior to and following the collection of biopsies.

5.3 Exclusion Criteria

Males and transgender females who meet any of the following criteria are excluded from the study:

1. At Screening, participant-reported symptoms, and/or clinical or laboratory diagnosis of active anorectal or reproductive tract infection requiring treatment per current World Health Organization (WHO) guidelines or symptomatic urinary tract infection (UTI). Infections requiring treatment include symptomatic *Chlamydia trachomatis* (CT) infection, *Neisseria gonorrhea* (GC), syphilis, active herpes simplex virus (HSV) lesions, anogenital sores or ulcers, or symptomatic genital warts.

    Note: HSV-1 or HSV-2 seropositive diagnosis with no active lesions is allowed, since treatment is not required.

    In cases of non-anorectal GC/CT identified at screening, one re-screening 2 months after the screening visit will be allowed

2. History of inflammatory bowel disease as reported by participant history

3. At Screening:

    a. Positive for hepatitis B surface antigen
    b. Positive for hepatitis C antibody
    c. Hemoglobin < 10.0 g/dL
    d. Platelet count less than 100,000/mm^3
    e. White blood cell count < 2,000 cells/mm^3 or > 15,000 cells/mm^3
    f. Calculated creatinine clearance less than 60 mL/min by the Cockcroft-Gault formula where creatinine clearance in mL/min = (140 - age in years) x (weight in kg) x (1 for male)/72 x (serum creatinine in mg/dL)

g. Serum creatinine > 1.3 x the site laboratory upper limit of normal (ULN)
h. Alanine transaminase (ALT) and/or aspartate aminotransferase (AST) > 2.5× the site laboratory ULN
i. *PK, PD and Immunological Subset only*: International normalized ratio (INR) > 1.5× the site laboratory ULN or partial thromboplastin time (PTT) > 1.25× the site laboratory ULN

4. Known allergy to methylparaben and/or propylparaben

5. Known allergy to any of the study products.

6. Per participant report, use of the following medications and/or products within 12 weeks prior to screening, and/or anticipated use or unwillingness to abstain from use throughout study participation:

   a. Any investigational products
   b. Systemic immunomodulatory medications
   c. Use of Heparin, including Lovenox®
   d. Warfarin
   e. Plavix® (clopidogrel bisulfate)
   f. Rectally-administered medications or products, containing N-9 or corticosteroids

7. By participant report, use of post-exposure prophylaxis (PEP) for HIV exposure within the 12 weeks prior to screening or anticipated use during study participation.

8. Symptoms suggestive of acute HIV seroconversion at Screening and Enrollment

9. Has any other condition that, in the opinion of the IoR/designee, would preclude informed consent, make study participation unsafe, complicate interpretation of study outcome data, or otherwise interfere with achieving the study objectives would make the patient unsuitable for the study or unable/unwilling to comply with the study requirements. Such conditions may include, but are not limited to, colorectal abnormalities, substance abuse, or renal, hepatic, hematological, gastrointestinal, endocrine, pulmonary, neurological or psychiatric disease.

5.4 Co-enrollment Guidelines

As indicated in Section 5.2, participants should not take part in other research studies involving drugs, medical devices, vaccines or genital products after the Screening Visit and while taking part in MTN-017, unless approved by the Protocol Safety Review Team (PSRT) and Protocol Chair and Co-Chair. Participation in the following types of studies may be allowed at the discretion of the IoR/designee:

- Participants may take part in ancillary studies approved by MTN-017 Protocol Chair and Co-Chair
- Participants who become infected with HIV may take part in observational and/or interventional studies for HIV-positive persons

Should any participant report concurrent participation in contraindicated studies after enrolling in MTN-017, the IoR/designee will consult the PSRT regarding ongoing product use and other potential safety considerations associated with co-enrollment.

6 STUDY PRODUCT

6.1 Regimen

Study participants will be randomized to one of six regimen sequences (Sequence 1-6). Each sequence will consist of three eight week periods of study product administration followed by at least a one-week washout period. The duration of product administration including the two washout periods is approximately 26 weeks.

Participants will receive study product that includes once-daily rectally-administered TFV RG 1% gel, once daily FTC/TDF tablet, and RAI-dependent rectally-administered TFV RG 1% gel (BAT 24) dosing. BAT 24 dosing refers to how participants will be instructed to insert the TFV RG 1% gel before and after sex. Participants will be instructed to insert one dose of TFV RG 1% gel up to 12 hours prior to RAI and one applicator of gel as soon as possible after RAI, but within 12 hours of intercourse. Participants will be instructed not to insert more than two doses within a 24 hour period. The products will be administered in the order designated by their randomized sequence (1-6).

Table 3: Study Regimen

Sequence	**Period 1** (8 weeks)	Washout (~1 week)	**Period 2** (8 weeks)	Washout (~1 week)	**Period 3** (8 weeks)	**Follow-up** (~1 Week)
1	Oral (Daily FTC/TDF)		Rectal (Daily TFV RG 1% gel)		Rectal (RAI-associated TFV RG 1% gel)	
2	Rectal (RAI-associated TFV RG 1% gel)		Oral (Daily FTC/TDF)		Rectal (Daily TFV RG 1% gel)	
3	Rectal (Daily TFV RG 1% gel)		Rectal (RAI-associated TFV RG 1% gel)		Oral (Daily FTC/TDF)	
4	Rectal (Daily TFV RG 1% gel)		Oral (Daily FTC/TDF)		Rectal (RAI-associated TFV RG 1% gel)	
5	Oral (Daily FTC/TDF)		Rectal (RAI-associated TFV RG 1% gel)		Rectal (Daily TFV RG 1% gel)	
6	Rectal (RAI-associated TFV RG 1% gel)		Rectal (Daily TFV RG 1% gel)		Oral (Daily FTC/TDF)	

6.2 Administration

6.2.1 FTC/TDF 200mg/300mgTablet (Truvada®)

Study participants will be instructed to take one FTC/TDF tablet, by mouth, once daily for the 8-week study period. FTC/TDF should be taken close to the same time each day. If a participant misses a dose, the missed dose should be taken as soon as possible, unless the next dose is estimated to be due within 6 hours. If the next dose is estimated to be due within 6 hours, the missed dose must be skipped. The next dose must be taken as originally scheduled.

6.2.2 TFV RG 1% gel

Daily TFV RG 1% gel
Study participants will be instructed to insert one dose (the entire content of one applicator) of TFV RG 1% gel into the rectum, once daily for the 8-week study period. The gel should be inserted close to the same time each day.

If a participant misses a dose, the missed dose should be inserted or taken as soon as possible, unless the next dose is estimated to be due within 6 hours. If the next dose is estimated to be due within 6 hours, the missed dose must be skipped. The next dose will be inserted rectally as originally scheduled.

RAI-Associated TFV RG 1% gel
During the RAI-associated TFV RG 1% gel administration for the 8-week study period, participants will be instructed to dose using the BAT 24 dosing regimen. Participants will be instructed to insert one dose of TFV RG 1% gel up to 12 hours prior to RAI and one applicator of gel as soon as possible after RAI, but within 12 hours of intercourse. Participants will be instructed not to insert more than two doses within a 24 hour period. In the event that a participant does not engage in RAI in the preceding 6 days, they will receive instructions to apply gel using the BAT 24 regimen on the 7th day. Thus, all participants will administer a minimum of two doses per week during the RAI-associated period.

6.3 Formulation

6.3.1 FTC/TDF 200mg/300mgTablet (Truvada®)

FTC/TDF is a fixed dose combination tablet containing FTC and TDF. FTC is a synthetic nucleoside analogue of cytidine. One FTC/TDF tablet contains 200 mg FTC plus 300 mg of TDF. FTC/TDF should be stored at 25°C (77°F). Excursions are permitted between 15°C and 30°C (59°F and 86°F).

6.3.2 Tenofovir RG 1% Gel

TFV RG 1% gel (weight/weight) is a transparent gel formulation of tenofovir (PMPA, 9-[(R)-2-(phosphonomethoxy)propyl]adenine monohydrate), formulated in purified water with edetate disodium, citric acid, glycerin, methylparaben, propylparaben, hydroxyethylcellulose, and pH adjusted to 4-5. The TFV RG 1% gel has lower glycerin content than the TFV 1% gel (original vaginal formulation) and a significantly reduced osmolality (approximately 800 versus

approximately 3000 mmol/kg, respectively). Compared with the TFV 1% gel, the TFV RG 1% gel contains an increased concentration of HEC (not considered significant) to maintain the viscosity similar to the original gel. The TFV RG 1% gel will be filled into applicators to form pre-filled, single-use applicators. Each pre-filled applicator will contain a dose of approximately 4 grams (equal to approximately 4 mL) of TFV RG 1% gel for delivery. The TFV RG 1% gel must be stored at controlled room temperature, 25°C (77°F). Excursions are permitted between 15°C and 30°C (59°F and 86°F).

6.4 Supply and Accountability

6.4.1 Supply

FTC/TDF 200mg/300mgTablet (Truvada®)
FTC/TDF tablets are supplied by Gilead Sciences, Inc. (Foster City, CA, USA).

TFV RG 1% gel
The TFV RG 1% gel is supplied by CONRAD (Arlington, VA, USA).

6.4.2 Accountability

The Clinical Research Site (CRS) Pharmacist of Record (PoR) is required to maintain complete records of all study products received. All unused study products must be returned to the MTN CORE Pharmacist after the study is complete unless otherwise instructed by the MTN CORE Pharmacist. Procedures to be followed will be provided in the MTN-017 Pharmacy Manual.

6.4.3 Study Product Dispensing

Study products will be dispensed by the pharmacist to enrolled study participants or to study staff on behalf of the participant, upon receipt of a written prescription from an authorized prescriber. An authorized prescriber includes the IoR or a licensed clinician directly responsible to the IoR as noted on the FDA 1572 Form.

Tablets or pre-filled applicators of study gel will be dispensed in quantities sufficient to last until the next scheduled study visit. Dispensing will take place at Initiate Period Visits and at Mid-Period Visits. In the event that additional study products between visits are needed, participants will be instructed to contact the study site. The pharmacist will record the dispensing of any additional study product on the documents maintained by the PoR or designee.

6.4.4 Male Condoms and Lubricant

All participants will be offered male condoms. The condoms will be made available in the clinic and will be dispensed by the clinic staff. Clinic staff will also offer participants a lubricant approved for use during the gel administration periods to facilitate applicator insertion.

6.4.5 Retrieval of Unused Study Products

Study participants will be instructed to return all unused study products to the site at each scheduled study visit. In the event that unused study products are not returned at the end of

each study period visit, site staff members will make every effort to encourage participants to return study product as soon as possible. If study product is not returned within the time frames outlined below the MTN-017 PSRT must be notified.

6.4.6 Study Product Permanent Discontinuation

Participants who are permanently discontinued from study product use will be instructed to return all unused study product to the site. The PoR will store returned unused study products in designated areas within the study pharmacy. Following permanent discontinuation and the Period End Visits, study product must be returned within 5 working days. If it is not returned within 5 working days, study staff will conduct outreach to retrieve the unused product from the participant (e.g., at their home).

Oral and gel study product must be retrieved within 24 hours when product use is permanently discontinued for HIV seroconversion.

Oral study product must be retrieved within 24 hours when product use is permanently discontinued due to Grade 3 or higher renal or hepatic toxicity.

6.4.7 Study Product Temporary Hold

Study product use for a participant may require a temporary hold. If oral or gel study product use is being held with expected duration of at least 7 days, the product should be retrieved within 7 working days. It is not necessary to retrieve study product from participants whom product use is being held for less than 7 days.

6.5 Adherence Counseling

Study product adherence counseling will be provided to all study participants by site staff. Counseling will be provided in accordance with standard methods based on participant-centered strategies with discussions focused on describing experiences and identifying factors facilitating the ease/comfort of product use. Participants will also be counseled against product sharing and the impact of this could have on the outcome of the trial. Counseling will be non-judgmental and will aim to create a supportive environment where participants can discuss their experience using the products while feeling empowered that gel or pill use are decisions they are free to make. To that end, there will be no consequences if study product sharing is revealed. Additional counseling regarding the issue of sharing will be provided. Towards the end of the discussion, counselors will help participants address what they can do or what can be done to increase ease/comfort/efficacy of product use.

6.6 Concomitant Medications and Practices

With the exception of those listed below as prohibited, enrolled participants may use concomitant medications permitted during study participation. Throughout the course of the study, all concomitant medications, including those used to treat AEs, will be recorded on forms designed for that purpose. Concomitant use of emtricitabine, tenofovir disoproxil fumarate or tenofovir disoproxil fumarate/ emtricitabine or any other medication for pre- and post- exposure prophylaxis for possible HIV exposure may be permitted at the discretion of the IoR. If any of

these agents become standard of care for HIV prevention their concomitant use is permitted. Prescription medications, over-the-counter preparations, vitamins and nutritional supplements, and herbal preparations will be recorded as concomitant medications.

Note: Use of lubricants, douches and/or enemas that do not contain N-9 or corticosteroids are permitted by the study. Information regarding the use of these products will be recorded at all scheduled clinic visits.

6.7 Prohibited Medications and Practices

Study participants will be advised not to use the following products within 12 weeks of the Screening Visit and throughout study participation:, systemic immunomodulatory medications, Heparin, including Lovenox®, Warfarin, Plavix® (clopidogrel bisulfate), rectally-administered medications or products, containing N-9 or corticosteroids, or any investigational products unless otherwise permitted.

Furthermore, a subset of participants will be counseled not to use NSAIDs, aspirin and/or other drugs that are associated with increased likelihood of bleeding for 72 hours prior to and following mucosal biopsies. Should a participant report the use of such drugs within 72 hours prior to a biopsy visit, collection of biopsies at that visit will be performed at IoR discretion. Participants will be appropriately counseled regarding the potential risks and documentation of the decision process will be included in the participants' study documents. Rapid PSRT consultation can be requested at IoR discretion, if needed.

7 STUDY PROCEDURES

An overview of the study visit and evaluations schedule is provided in Appendix I. Presented in this section is additional information on visit-specific study procedures. Detailed instructions to guide and standardize procedures across sites are provided in the MTN-017 SSP Manual available at www.mtnstopshiv.org.

Figure 2: Study Schedule

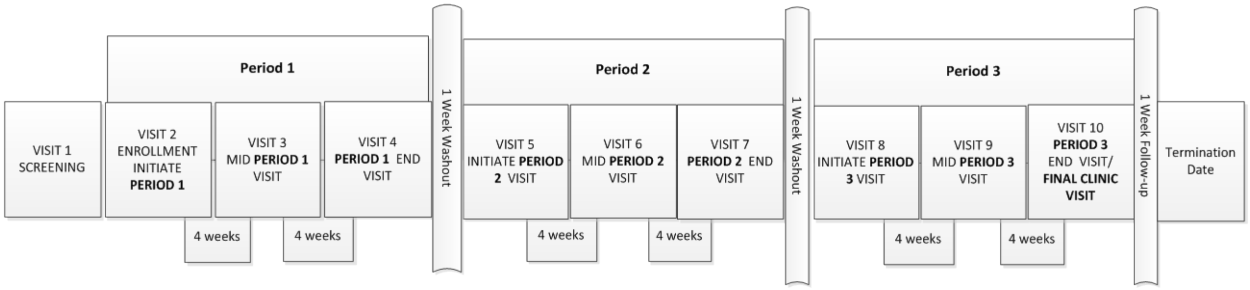

7.1 Screening Visit

Screening may take place up to 30 days prior to the Enrollment/Initiate Period 1 Visit. Multiple screening visits may be conducted, if needed, to complete all required procedures. Written informed consent will be obtained before any screening procedures are initiated. Screening will discontinue once ineligibility is determined for participants who do not meet the eligibility criteria.

Table 4: Screening

<table>
<tr><th colspan="3">Screening Visit</th></tr>
<tr><th colspan="2">Component</th><th>Procedures</th></tr>
<tr><td colspan="2">Administrative and Regulatory</td><td>• Obtain written informed consent
• Assign Participant Identification Number (PTID)
• Collect demographic data
• Collect locator information
• Assess eligibility
• Provide reimbursement
• Schedule next study visit/contact
• Provide available test results</td></tr>
<tr><td colspan="2">Counseling</td><td>• Provide HIV pre-/post-test and HIV/STI risk reduction counseling

<u>Rectal Biopsy/Fluid Subset only:</u>
• Rectal biopsy/fluid procedural counseling</td></tr>
<tr><td colspan="2">Clinical</td><td>• Collect medical history
• Perform physical exam
• Perform rectal exam
• Collect concomitant medications
• Treat for UTIs/RTIs/STIs or refer for other findings*</td></tr>
<tr><td rowspan="3">Laboratory</td><td>Urine</td><td>• Dipstick UA
• NAAT for GC/CT</td></tr>
<tr><td>Blood</td><td>• Complete blood count (CBC) with differential and platelets
• AST/ALT
• Creatinine
• Syphilis rapid plasma reagin (RPR)
• HIV-1 serology
• HSV 1/2 antibody
• HBsAg
• Hepatitis C antibody

<u>Rectal Biopsy/Fluid Subset only:</u>
• Coagulation (PT/INR)</td></tr>
<tr><td>Rectal</td><td>• Collect rectal swab for NAAT for GC/CT
• HSV 1/2 detection*</td></tr>
<tr><td colspan="2">Study Product/Supplies</td><td>• Provision of male condoms</td></tr>
</table>

*If indicated

7.2 Study Visits

Windows for study visits will be provided in the SSP.

7.2.1 Period 1- Visit 2 (Enrollment Visit, Day 0), Period 2- Visit 5 (Week 9), Period 3- Visit 8 (Week 18)

If ineligibility is determined at Visit 2- Enrollment all procedures will discontinue. At Visit 5 and Visit 8, prior to the initiation of study product, all AEs need to have resolved or stabilized. If the adverse event has not resolved within 7 days following Visit 2 or 7, the PSRT should be consulted regarding progression into the next dosing period.

Table 5: Initiate Period Visits

<table>
<tr><th colspan="3">Period 1- Visit 2 (Enrollment Visit, Day 0), Period 2- Visit 5 (Week 9),
Period 3- Visit 8 (Week 18)</th></tr>
<tr><th colspan="2">Component</th><th>Procedures</th></tr>
<tr><td colspan="2">Administrative and Regulatory</td><td>• Review/Update locator information
• Confirm participant eligibilityǂ
• Provide reimbursement
• Schedule next study visit/contact
• Provide available test results
• Randomizationǂ</td></tr>
<tr><td colspan="2">Behavioral/ Counseling/Participant Orientation</td><td>• Administer baseline behavioral questionnaire (CASI)ǂ
• Provide HIV pre-/post-test and HIV/STI risk reduction counselingǂ*
• Provide protocol adherence counseling
• Product use instructions and adherence counseling
Note: See Section 7.9 for specific instructions regarding study product initiation for participants in the Rectal Biopsy/Fluid Subset who are randomized to daily TFV RG 1% gel and RAI-associated TFV RG 1% gel during Period 1.

<u>Rectal Biopsy/Fluid Subset only:</u>
• Rectal biopsy/fluid procedural counselingǂ</td></tr>
<tr><td colspan="2">Clinical</td><td>• Review/Update medical history
• Review/Update concomitant medications
• Perform physical examǂ*
• Perform rectal exam
• Record/Update AEs
• Treat for UTIs/RTIs/STIs or refer for other findings*
• Hepatitis B vaccination or documentation of declination of vaccination*

Note: Participants found to be negative for HBsAg at screening are given information and offered the HBV vaccine series starting at their enrollment visits. For enrolled participants who are susceptible but decline vaccination at enrollment, the vaccine series may be initiated at any time during follow-up. The hepatitis B vaccine is not considered a study product in MTN-017.</td></tr>
<tr><td rowspan="2">Laboratory</td><td>Urine</td><td>• Dipstick UA*
• NAAT for GC/CT*</td></tr>
<tr><td>Blood</td><td>• Plasma archiveǂ
• HIV-1 serologyǂ*
• CBC with differential and platelets*</td></tr>
</table>

		• AST*/ALT*<br>• Creatinine*<br>• Syphilis RPR*
	**Rectal**	• HSV 1/2 detection*<br>• Anal human papillomavirus (HPV)╪<br>• Collect rectal sponge for PD<br>• Collect rectal sponge for adherence PK^<br>• NAAT for GC/CT╪*<br><br><u>Rectal Biopsy/Fluid Subset only:</u><br>• Collect rectal sponge for mucosal immunology╪<br>• Collect rectal biopsies for PD╪<br>• Collect rectal biopsies for mucosal immunology╪
**Study Product/Supplies**		• Provision of study product<br>• Observe first dose and/or simulation of first dose with SMS training<br>• Provision of male condoms<br>• Provision of lubricant*

* If indicated, ╪ Mandatory at Visit 2 (Enrollment/Initiate Period 1), ^ Visit 5 and 8 only

7.3 Follow-Up Phone Calls

Study staff should follow the guidelines provided in Section 9.0 if AEs are reported via phone contact.

Study staff will follow-up with participants via phone call 48-72 hours and two weeks following the expected date of study product initiation for each period (Period 1, 2 and 3). Study staff will inquire about AEs they might have experienced as a result of the study product or procedures performed during the preceding visit.

All participants will be instructed to call-in to the clinic to report any new or worsening AEs within 7 days following the Final Clinic Visit 10 (Week 26).

<u>Rectal Biopsy/Fluid Subset only:</u>
Participants taking part in the Rectal Biopsy/Fluid Subset will be called 48-72 hours after Period 1 End Visit and Period 2 End Visit.

Table 6: Follow-Up Phone Call

Follow-up Phone Call	
**Component**	**Procedures**
**Administrative and Regulatory**	• Reimbursement~
**Clinical**	• Record/update AEs

~ Sites to reference SOPs regarding participant reimbursement

7.4 Mid-Period Visits

7.4.1 Period 1- Visit 3 (Week 4), Period 2- Visit 6 (Week 13), Period 3- Visit 9 (Week 22)

The procedures listed below will be performed approximately 4 weeks after the participant initiates Period 1, Period 2 and Period 3.

Table 7: Mid-Period Visits

<table>
<tr><th colspan="3">Period 1- Visit 3 (Week 4), Period 2- Visit 6 (Week 13), Period 3- Visit 9 (Week 22)</th></tr>
<tr><th colspan="2">Component</th><th>Procedures</th></tr>
<tr><td colspan="2">Administrative and Regulatory</td><td>• Review/Update locator information
• Schedule next study visit
• Provision of available test results
• Provide reimbursement</td></tr>
<tr><td colspan="2">Behavioral/Counseling</td><td>• Provide HIV pre-/post-test and HIV/STI risk reduction counseling
• Provide protocol adherence counseling
• Product use instructions and adherence counseling
• Conduct Data Convergence Interview</td></tr>
<tr><td colspan="2">Clinical</td><td>• Review/update medical history
• Review/update concomitant medications
• Perform rectal exam
• Perform physical exam*
• Record/update AEs
• Treat for UTIs/RTIs/STIs or refer for other findings*
• Hepatitis B vaccination or documentation of declination of vaccination*

Note: Participants found to be HBV susceptible at screening are given information and offered the HBV vaccine series starting at their enrollment visits. For enrolled participants who are susceptible but decline vaccination at enrollment, the vaccine series may be initiated at any time during follow-up. The hepatitis B vaccine is not considered a study product in MTN-017.</td></tr>
<tr><td rowspan="3">Laboratory</td><td>Urine</td><td>• Dipstick UA*
• NAAT for GC/CT*</td></tr>
<tr><td>Blood</td><td>• HIV-1 serology
• CBC with differential and platelets*
• AST*/ALT*
• Creatinine*
• Syphilis RPR*</td></tr>
<tr><td>Rectal</td><td>• Collect rectal sponge for adherence PK
• Collect rectal sponge for PD
• Collect rectal swab for NAAT for GC/CT*
• HSV 1/2 detection*</td></tr>
<tr><td colspan="2">Study Product/Supplies</td><td>• Provision of study product
• Collect unused product
• Provision of male condoms
• Provision of lubricant*</td></tr>
</table>

***** If indicated

7.5 Period End Visits

7.5.1 Period 1- Visit 4 (Week 8) and Period 2- Visit 7 (Week 17), Period 3- Final Clinic Visit/Early Termination- Visit 10 (Week 26)

The following procedures will be completed approximately 8 weeks after the participant initiates product use during Period 1, Period 2 and Period 3.

Table 8: Period End Visits

<table>
<tr><th colspan="3">Period 1- Visit 4 (Week 8) and Period 2- Visit 7 (Week 17), Period 3- Final Clinic Visit/Early Termination- Visit 10 (Week 26)</th></tr>
<tr><th colspan="2">Component</th><th>Procedures</th></tr>
<tr><td colspan="2">Administrative and Regulatory</td><td>• Review/Update locator information
• Schedule next study visit or contact▲*
• Provision of AE dial-in instructions (See Section 7.3)♦
• Provision of available test results
• Provide reimbursement</td></tr>
<tr><td colspan="2">Behavioral/Counseling</td><td>• Follow-up behavioral questionnaire (CASI)
• In-depth phone interview on a subset of participants∆
• Data Convergence Interview
• HIV pre-/post-test and HIV/STI risk reduction counseling

<u>Rectal Biopsy/Fluid Subset only:</u>
• Rectal biopsy/fluid procedural counseling</td></tr>
<tr><td colspan="2">Clinical</td><td>• Review/update medical history
• Review/update concomitant medications
• Perform a rectal exam
• Perform physical exam♦*
• Record/update AEs
• Treat for UTIs/RTIs/STIs or refer for other findings*
• Hepatitis B vaccination or documentation of declination of vaccination*

Note: Participants found to be HBV susceptible at screening are given information and offered the HBV vaccine series starting at their enrollment visits. For enrolled participants who are susceptible but decline vaccination at enrollment, the vaccine series may be initiated at any time during follow-up. The hepatitis B vaccine is not considered a study product in MTN-017.</td></tr>
<tr><td rowspan="2">Laboratory</td><td>Urine</td><td>• Dipstick UA*
• NAAT for GC/CT♦*</td></tr>
<tr><td>Blood</td><td>• CBC with differential and platelets ♦*
• AST♦*, ALT♦*
• Creatinine
• Plasma for storage
• Blood for PK
• HIV-1 serology
• Syphilis RPR*</td></tr>
</table>

	**Rectal**	• Collect rectal sponge for adherence PK<br>• Collect rectal sponge for PD<br>• Collect rectal swab for NAAT for GC/CT<br>• HSV 1/2 detection*<br><br><u>Rectal Biopsy/Fluid Subset only:</u><br>• Collect rectal sponge for mucosal immunology<br>• Collect rectal biopsies for PK<br>• Collect rectal biopsies for PD<br>• Collect rectal biopsies for mucosal immunology
**Study Product/Supplies**		• Collect unused study product<br>• Provision of male condoms

* If indicated, ▲Mandatory at Visits 4 and 7, ∆ Mandatory at Visit 4, ♦ Mandatory at Visit 10

7.6 Follow-up Procedures for Participants Who Discontinue Study Product

A participant who discontinues study product will be encouraged to remain in the study if they are willing, for safety evaluations according to the study follow-up schedule with the following exceptions described in the sections below.

7.6.1 Participants Who Become Infected with HIV-1

Participants who become infected with HIV-1 will be referred for medical care. Participants who seroconvert while enrolled in MTN-017 will undergo additional procedures. CD4, HIV RNA and HIV drug-resistance testing will be performed. Study staff, with written permission from the participant, may contact the medical care provider to inform him/her of the participant's involvement in MTN-017. The participant will be offered the option to continue follow-up visits per the original study schedule until his/her originally scheduled study exit date. For those who choose to remain in follow-up, protocol-specified procedures will continue except the following:

- HIV-1 serology
- Provision of study product
- Rectal exams, unless required for follow-up of AE
- PK, PD and mucosal immunology specimen collection
- Provision of counseling:
    - HIV pre- and post-test
    - Adherence (Product use/protocol)

HIV/STI risk reduction counseling will be modified to address primary and secondary prevention for infected men and infected transgender females.

In the event a participant becomes infected with HIV-1, the behavioral-related assessments will be administered according to guidance from the protocol team.

7.6.2 Participants who Either Voluntarily or by the Site Investigator Discretion Temporarily or Permanently Discontinue Study Product Use

All protocol-specified study procedures will continue except:

- Provision of study product
- Provision of product use/protocol adherence counseling
- Rectal exams, unless required for AE follow-up

If scheduled for collection, the following procedures will be performed as follows:

- PK, PD and mucosal immunology specimen collection and associated procedures will be performed, ideally, at the visit in which study product use is temporarily or permanently discontinued, but may be collected up to 2 weeks following the temporary/permanent discontinuation of product.
- The following behavioral assessments will be performed at the visit in which study product use is temporarily or permanently discontinued, or at the visit in which site staff first learn that the participant has stopped/intends to stop study product use
    - Follow-up Behavioral Questionnaire
    - Data Convergence Interview
    - In-depth Phone Interview

Note: In the event study product is stopped at an interim visit, the SSP should be referenced for procedural guidance, as some of the aforementioned procedures may need to be collected at a follow-up visit.

These study procedures will be omitted at subsequent visits that occur during the time off study product. Completion of these procedures will resume if and when the participant resumes study product use.

7.7 Interim Visits

Interim visits may be performed at any time during the study. All interim contacts and visits will be documented in participants' study records and on applicable CRFs.

Table 9: Interim Visit(s)

Interim Visit(s)	
**Component**	**Procedures**
**Administrative and Regulatory**	• Review/update locator information*<br>• Schedule next visit or contact*<br>• Disclosure of available test results*
**Counseling**	• Provide participant-HIV pre-/post-test and HIV/STI risk reduction counseling*<br>• Participant-adherence counseling*

<table>
<tr><td colspan="2">Clinical</td><td>Record/update AEs*
Review/update medical history*
Review/update concomitant medications*
Perform a rectal exam*
Perform a physical exam*
Treat for UTIs/RTIs/STIs or refer for other findings*</td></tr>
<tr><td rowspan="3">Laboratory</td><td>Urine</td><td>NAAT for GC/CT*
Dipstick UA*</td></tr>
<tr><td>Blood</td><td>Chemistries*
CBC with differential and platelets*
HIV-1 serology*
Blood for PK*
Plasma for storage*
Hepatitis B vaccination or documentation of declination of vaccination*
Note: Participants found to be HBV susceptible at screening are given information and offered the HBV vaccine series starting at their enrollment visits. For enrolled participants who are susceptible but decline vaccination at enrollment, the vaccine series may be initiated at any time during follow-up. The hepatitis B vaccine is not considered a study product in MTN-017.</td></tr>
<tr><td>Rectal</td><td>HSV 1/2 detection*
Collect rectal swab for NAAT for GC/CT*</td></tr>
<tr><td colspan="2">Study Product</td><td>Collect unused study product*
Provision of study product*
Provide male condoms*
Provision of lubricant*</td></tr>
</table>

* if indicated

7.8 Pharmacokinetics and Pharmacodynamics

All participants will have blood (plasma and PMBCs) collected for PK. It is anticipated that a maximum of 24 mL (3 8-mL CPTs) will be required for PK at each time point (Period 1 End, Period 2 End, and Period 3 End). In addition, rectal fluid will be collected via sponge for PK and PD.

Table 10: PK and PD Sample Collection Schedule

Specimens	All Participants	
	**PK Specimen Collection Adherence**	**PD Specimen Collection**
**Blood (Plasma and PBMC)**	Period 1 End<br>Period 2 End<br>Period 3 End	

Specimens	All Participants	
	PK Specimen Collection Adherence	PD Specimen Collection
**Rectal Fluid**	Mid-Period 1<br>Period 1 End<br>Initiate Period 2<br>Mid-Period 2 Use<br>Period 2 End<br>Initiate Period 3<br>Mid-Period 3 Use<br>Period 3 End	Enrollment<br>Mid-Period 1<br>Period 1 End<br>Initiate Period 2<br>Mid-Period 2<br>Period 2 End<br>Initiate Period 3<br>Mid-Period 3<br>Period 3 End

7.9 Intensive Pharmacokinetics, Pharmacodynamics and Immunological Sample Collection

A subset of approximately 36 participants (the Rectal Biopsy/Fluid Subset) enrolled at designated rectal biopsy/fluid subset sites will have rectal fluid and biopsies collected at each of the following time points, Enrollment, Period 1 End, Period 2 End and Period 3 End Visits, for PK, PD and mucosal immunology. Approximately 15 biopsies will be collected at Enrollment and approximately 20 will be collected at all other time points. The rationale for including 36 participants is based upon previously completed Phase 1 studies with similar sampling parameters. Participants at designated rectal biopsy/fluid subset site(s) will be able to opt-in to participation. Participants must agree to abstain from inserting anything into the rectum, including abstaining from using the study gel and having RAI for 72 hours after the collection of these samples. In addition, participants will be instructed to refrain from the use of NSAIDs, aspirin and/or other drugs that are associated with the increased likelihood of bleeding for 72 hours prior to and following mucosal biopsy collection. Detailed instructions to guide and standardize procedures, including the initiation of study product following the Enrollment Visit for participants randomized to daily TFV RG 1% gel, are provided in the MTN-017 SSP Manual available at www.mtnstopshiv.org.

Table 11: PK/PD/Immunology Sample Collection Schedule

Specimens	Subset of Participants N= Approximately 36		
	PK Specimen Collection	PD Specimen Collection	Mucosal Immunology
**Rectal Sponge**			Enrollment<br>Period 1 End<br>Period 2 End<br>Period 3 End
**Rectal Biopsies**	Period 1 End<br>Period 2 End<br>Period 3 End	Enrollment<br>Period 1 End<br>Period 2 End<br>Period 3 End	Enrollment<br>Period 1 End<br>Period 2 End<br>Period 3 End

7.10 Behavioral Measures

The behavioral measures of this protocol will focus mainly on acceptability of and adherence to gel and pill use.

7.10.1 Acceptability of Gel and Pill Use

Microbicide acceptability has been defined as the voluntary sustained use of a microbicide product in the context of alternatives.[7] As described by Morrow and Ruiz[35], microbicide acceptability in MTN-017 will be assessed considering the following factors:

- **Vehicle-associated:** Formulation, texture and viscosity, product scent, color, taste, and desirable/appealing elements of product vehicle
- **Application-associated:** Clarity of instructions, ability to adhere to instructions, ease of product preparation, and ease of application
- **Use-associated:** Frequency and timing of product use, partner-specific (e.g., main or casual) , odor post-application and during or after sex, leakage post-application and during or after sex, lubrication and drying effects including post-application and during or after sex, product consistency post-use, desirable/appealing elements of use, use with/without condom, changes in hygiene practices secondary to use, changes in sexual pleasure secondary to use
- **Related Co-variables:** History of anal product use, frequency of anal sex, relationship ‘‘harmony’’; relationship communication

We will use the following behavioral assessments to evaluate the factors listed above:

1. Baseline Behavioral Questionnaire
2. Follow-up Behavioral Questionnaire- Acceptability Component
3. In-depth Phone Interviews

Baseline Behavioral Questionnaire

All participants will complete a CASI questionnaire at the Visit 2 (Enrollment/Initiate Period 1) at a private computer terminal located at the study site. In addition to demographics, this questionnaire will explore participants’ sexual risk behavior and other behavioral practices, will include a baseline rating for likelihood of using a microbicide in the form of a gel or pill in the future, as well as a survey of practices associated with anal intercourse that may affect microbicide use, i.e., problem practices.

Note: These data will not be available in real-time, no adjustment of counseling will be performed based on problem practices reported.

Follow-up Behavioral Questionnaire

Acceptability Component

All participants will complete a behavioral questionnaire via CASI. The Acceptability Component (Gel) will be administered both after the participant has completed daily gel use and RAI-associated gel use periods. It will explore the participants’ sexual risk behavior and other behavioral practices associated with product use, experiences using the rectally-administered gel, any problems they

may have had, product side-effects and likelihood of using a rectally-applied microbicide in the future. The Acceptability Component (Pill) will be administered after 8 weeks of pill use. It will explore participants' sexual risk behavior and other behavioral practices associated with product use, experiences taking the pill, reasons that prevented them from taking it (including any pill sharing), any problems the participant may have had or product side effects, and likelihood of using a pill for HIV-prevention in the future.

In addition, participants will be asked whether they prefer using a gel daily or with RAI or taking a pill. Our acceptability measurement will take into account ease of use, liking the products, and likelihood of product use and will compare acceptability ratings using three paired t-tests: the pill vs. the daily gel, the pill vs. the gel with RAI, and for the daily gel vs. gel with RAI.

Problem Practices Component
Follow-up assessment of practices associated with anal intercourse that may affect microbicide use, i.e., problem practices, will be evaluated within the Follow-up Behavioral Questionnaire at the Period End Visits.

Note: These data will not be available in real-time, no adjustment of counseling will be performed based on problem practices reported.

7.10.2 Adherence

Following Pool et al.[36] and Tolley et al.[37] a mixed methods triangulation model will be used to increase the accuracy of adherence and sexual behavior measurement. Adherence data will be collected from several sources and discrepancies will be discussed with participants, analyzed and resolved (data convergence interview). The method is schematically presented in Figure 3.

Figure 3: Method for Converging Adherence

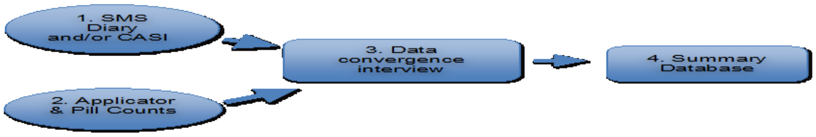

Adapted from Pool R, Montgomery CM, Morar NS, et al. A mixed methods and triangulation model for increasing the accuracy of adherence and sexual behavior data: The Microbicides Development Programme. Plos One 2010;5(7):e11600.

The following behavioral measures will be used to assess adherence:

Follow-up Behavioral Questionnaire- Adherence Component
At the Period End Visits, participants will respond to recall questions on adherence to gel and pill use via Computer-Assisted Self-Interview (CASI) within the Follow-up Behavioral Questionnaire. Questions will be worded so as to normalize lack of adherence.

Short Message Service (SMS) Diary
Short message service (SMS) will be employed as a measure to monitor adherence. Participants will indicate around what time of the day they expect to self-administer the product dose. A central server will be programmed to generate text messages on a daily basis around that time with a few short questions answerable with a couple of keystrokes. A monetary incentive will be tied to each session to encourage responses with participants being alerted that they will earn the incentive just for replying regardless of what their reply is. A bonus will also be given monthly to those who respond to 90% of the daily messages.

Applicator and Pill Counts
Participants will be instructed to save all unused study product (pills and gel applicators) and return them at their mid-product use and product use end visits. Site staff will tally all unused product and this will constitute a second measure of adherence. Participants will be asked to report if all missing product was used or if some unreturned product was not used (e.g., misplaced).

Participants will have received more doses than those needed for 4 weeks; therefore, there should always be remaining unused applicators and pills. Lack of returned study product may indicate product dumping or sharing and will be explored by the counselor during the Data Convergence Interview.

Data Convergence Interview
Data on self-reports of product use, sexual behavior, and condom use sent through SMS and/or collected via CASI, and study product counts will be entered on a single data comparison form that will be made available to the site counselor. The Data Convergence Interview will be conducted at mid-product use visits and product use end visits when there are discrepancies between adherence data collected from the SMS report and/or CASI and the returned study product counts. In such cases, the counselor will review the information with the participant to elicit information about possible discrepancies. The counselor's approach will be non-judgmental, reminding the participant that regardless of level of adherence, sexual behavior or condom use, they will not be disqualified from the study. These sessions will be audio recorded to ensure the quality and consistency of the counseling across all study sites. This analysis of data on the same topic emanating from different sources is generally referred to as research triangulation.[36] This process will allow for clarification of discrepancies between data sources and will be more informative than any single data source taken alone; all the independent data sources will be available to allow for analysis of different estimates of adherence.

In the rare case in which a participant attends the visit, returns the applicators, but does not stay to speak with a counselor, we will analyze the available data from the SMS system and the returned applicators to determine the most likely adherence rate on a case-by-case basis.

Summary Database
The data comparison form together with the converged result of most likely adherence to product use will constitute the summary database on adherence, sexual behavior and condom use during the study.

In-depth Phone Interviews
A subset of about 40 participants will participate in phone interviews to be held remotely by bilingual interviewers. Participants will be sequentially selected from each sequence as they complete their first study period at Visit 4. About half of the participants will be selected based on high adherence to product use, and the other half based on very poor adherence. It is anticipated that the participants may feel more comfortable discussing problems with product use with an interviewer that is not the same person who provided instructions on product use. Sensitive issues regarding sexual behavior and lack of condoms use are also likely to be enhanced with this procedure. Through open-ended questions using an interview guide, the interviews will explore factors across cultures that may facilitate or encourage adherence to product use and trial procedures. This interview will also explore product acceptability. The guide will have a summary section with pre-coded answers and summary fields so the interviewer can fill in the major findings during or immediately after the interview, thereby being able to give quick feedback to trial team during monthly phone calls. The interview will be audio recorded and transcribed.

7.11 Clinical Evaluations and Procedures

The following physical and rectal exam components will be conducted at select visits.

Physical Exam

- Height (may be omitted after the Screening Visit)
- Weight
- Vital signs
    - Temperature
    - Pulse
    - Blood pressure
- General appearance
- Abdomen
- Other components as indicated by participant symptoms

Rectal Exam and Specimen Collection
The participant will be positioned in the left lateral decubitus position for the following procedures:

- Visual and digital rectal exam: The examiner will conduct a visual examination of the anus and surrounding area and note any abnormality. The examiner will then insert a lubricated gloved finger into the anal canal and sweep around the internal anorectal circumference.
- Rectal swabs for GC/CT: A lubricated plastic anoscope will be gently and fully inserted (until the lateral ‘wings’ touch the anal margin) and the obturator removed. The anal swab will be

inserted through the anoscope and placed in contact with the rectal mucosa, turned around 360 degrees and removed.

- Rectal sponges for adherence PK, PD and mucosal immunology: A lubricated plastic anoscope will be gently and fully inserted (until the lateral 'wings' touch the anal margin) and the obturator removed. Sponges for PK, PD and mucosal immunology will be simultaneously inserted into the rectum so they are in contact with rectal mucosa proximal to the anoscope and held there for 2 minutes before removal. The anoscope will then be slowly removed.
- Flexible sigmoidoscopy and biopsy: A flexible sigmoidoscope will be inserted to 15 cm and biopsies taken using biopsy forceps on a subset of participants.
- Rectal lavage: A 125 mL of saline (0.9%) enema will be inserted through the anus and the contents squeezed into the rectum (Rectal Biopsy/Fluid Subset only).

7.12 Laboratory Evaluations

Local Laboratory

The local laboratory will run the following, as indicated:

- Urine specimens
  - Urinalysis
  - Urine GC/CT by NAAT

- Rectal specimens
  - Rectal GC/CT by NAAT via anoscope at sites with capacity

- Blood specimens
  - HIV-1 serology, with confirmatory testing as needed
  - CBC with differential and platelets
  - Syphilis testing by RPR with confirmatory testing as needed
  - Creatinine, AST, ALT
  - Hepatitis B surface antigen
  - Hepatitis C antibody
  - HSV serology +/-
  - HSV 1/2 viral detection
  - Coagulation (PT/INR)

Network Laboratory (NL)

The MTN NL will run the following, as indicated:

- Blood specimens
  - PK
  - Plasma archive
  - Plasma for storage (Confirmatory HIV serology)

- Rectal specimens
    - Rectal sponges via anoscope for:
        - Adherence PK
        - PD
        - Mucosal immunology (Rectal Biopsy/Fluid subset only)
    - Rectal GC/CT NAAT via anoscope at sites without capacity
    - Anal HPV typing via swab
    - On the Rectal Biopsy/Fluid Subset, biopsies via flexible sigmoidoscopy for:
        - PK
        - PD
        - Mucosal immunology

7.13 Specimen Collection and Processing

Each study site will adhere to the standards of good clinical laboratory practice, the MTN Network Laboratory Manual (http://www.mtnstopshiv.org), in accordance with current US Division of AIDS (DAIDS) Laboratory Requirements, MTN-017 Study-Specific Procedures Manual (http://www.mtnstopshiv.org), and site standard operating procedures for proper collection, processing, labeling, transport, and storage of specimens at the local laboratory. Specimen collection, testing, and storage at the site laboratories will be documented when applicable using the Laboratory Data Management System (LDMS). In cases where laboratory results are not available due to administrative or laboratory error, sites are permitted to re-draw specimens.

7.14 Specimen Handling

Specimens will be handled in accordance with current requirements for DAIDS Sponsored and/or Funded Laboratories in Clinical Trials. (http://www.niaid.nih.gov/labsandresources/resources/daidsclinrsrch/documents/labpolicy.pdf)

7.15 Biohazard Containment

Appropriate blood and secretion precautions will be employed by all personnel in the drawing of blood and shipping and handling of all specimens as recommended by the CDC and NIH. All biological specimens will be transported using packaging mandated by Code of Federal Regulations (CFR) 42 Part 72. All dangerous goods materials, including diagnostic specimens and infectious substances, must be transported according to instructions detailed in the International Air Transport Association (IATA) Dangerous Goods Regulations. Biohazardous waste will be contained according to institutional, transportation/carrier, and all other applicable regulations.

8 ASSESSMENT OF SAFETY

8.1 Safety Monitoring

Site IoRs are responsible for continuous close safety monitoring of all study participants. A sub-group of the Protocol Team, including the Protocol Chair and Co-Chair, DAIDS Medical Officer, Protocol Safety Physicians, CONRAD Representative and SCHARP Clinical Affairs Safety Associate will serve as the PSRT. The MTN Statistical Data Management Center (SDMC) prepares routine AE and clinical data reports or review by the PSRT, which meets via conference call approximately once per month or more frequently as needed throughout the period of study implementation to review safety data, discuss product use management, and address any potential safety concerns.

8.2 Clinical Data and Safety Review

A multi-tiered safety review process will be followed for the duration of this study. The study site investigators are responsible for the initial evaluation and reporting of safety information at the participant level, and for alerting the PSRT if unexpected concerns arise.

Participant safety is also monitored at the Network level through a series of routine reviews conducted by the SDMC Clinical Affairs staff, the PSRT and study sponsors. Additional reviews may be conducted at each of these levels as dictated by the occurrence of certain events.

MTN SDMC Clinical Affairs staff will review incoming safety data on an ongoing basis. Events identified as questionable, inconsistent, or unexplained will be queried for verification.

The PSRT will meet approximately every month via conference call to review clinical data reports generated by the MTN SDMC. The content, format and frequency of the clinical data reports will be agreed upon by the PSRT and the SDMC in advance of study implementation. In addition to the routine safety data reviews, the PSRT will convene on an ad hoc basis to make decisions regarding the handling of any significant safety concerns. If necessary, experts external to the MTN representing expertise in the fields of microbicides, biostatistics, HIV transmission and medical ethics may be invited to join the PSRT safety review. A recommendation to pause or stop the trial may be made by the PSRT at any such time that the team agrees that an unacceptable type and/or frequency of AEs has been observed.

If the protocol team has serious safety concerns they may request a review of data by the Study Monitoring Committee (SMC). SMC may recommend that the study proceed as designed, proceed with design modifications, or be discontinued. Members of the SMC will be independent investigators with no interest (financial or otherwise) in the outcomes of this study. If at any time a decision is made to discontinue enrollment and/or study product use in all participants, CONRAD will notify the FDA and the Clinical Research Site (CRS) Principal Investigator will notify the Institutional Review Board (IRB) expeditiously.

8.3 Adverse Events Definitions and Reporting Requirements

8.3.1 Adverse Events

An AE is defined as any untoward medical occurrence in a clinical research participant administered an investigational product and which does not necessarily have a causal relationship with the investigational product. As such, an AE can be an unfavorable or unintended sign (including an abnormal laboratory finding, for example), symptom or disease temporally associated with the use of an investigational product, whether or not considered related to the product. This definition is applied to all study sequences, and is applied to all sequences beginning at the time of enrollment (i.e., once participant is randomized). The term "investigational product" for this study refers to FTC/TDF (Truvada®), TFV RG 1% gel and the study gel applicator.

Study participants will be provided instructions for contacting the study site to report any AEs they may experience. In cases of potentially life-threatening events, participants will be instructed to seek immediate emergency care. Where feasible and medically appropriate, participants will be encouraged to seek evaluation where a study clinician is based, and to request that the clinician be contacted upon their arrival. With appropriate permission of the participant, whenever possible, records from all non-study medical providers related to AEs will be obtained and required data elements will be recorded on study clinical research forms (CRFs). All participants reporting an AE will be followed clinically until the AE resolves (returns to baseline) or stabilizes.

Study site staff will document in source documents and the appropriate AE Log CRF all AEs reported by or observed in enrolled study participants regardless of severity and presumed relationship to study product. AE severity will be graded per the Division of AIDS Table for Grading Adult and Pediatric Adverse Events, Version 1.0, December 2004 (Clarification dated August 2009) and the Rectal Grading Table for Use in Microbicide Studies, Addendum 3 in the Division of AIDS Table for Grading Severity of Adult and Pediatric Adverse Events, Version 1.0, December 2004 (Clarification dated May 2012) all available on the RSC website at http://rsc.tech-res.com/safetyandpharmacovigilance/. In cases where a genital AE is covered in both tables, the Rectal Grading Table for Use in Topical Microbicide Studies will be the grading scale utilized.

8.3.2 Serious Adverse Events

SAEs will be defined by the Manual for Expedited Reporting of Adverse Events to DAIDS (Version 2.0, January 2010), as any AEs that:

- Results in death
- Is life-threatening
- Results in persistent or significant disability/incapacity
- Is a congenital anomaly/birth defect
- Requires inpatient hospitalization or prolongation of existing hospitalization
  Note: Per ICH SAE definition, hospitalization itself is not an AE, but is an outcome of the event. Thus, hospitalization in the absence of an AE is not regarded as an AE, and is not

subject to expedited reporting. The following are examples of hospitalization that are not considered to be AEs:

- o Protocol-specified admission (e.g., for procedure required by study protocol)
- o Admission for treatment of target disease of the study, or for pre-existing condition (unless it is a worsening or increase in frequency of hospital admissions as judged by the clinical investigator)
- o Diagnostic admission (e.g., for a work-up of an existing condition such as persistent pretreatment lab abnormality)
- o Administrative admission (e.g., for annual physical)
- o Social admission (e.g., placement for lack of place to sleep)
- o Elective admission (e.g., for elective surgery)

Important medical events that may not result in death, be life-threatening, or require hospitalization may be considered a serious adverse drug experience when, based upon appropriate medical judgment, they may jeopardize the patient or subject and may require medical or surgical intervention to prevent one of the outcomes listed above.

8.3.3 Adverse Event Relationship to Study Product

Relatedness is an assessment made by a study clinician of whether or not the event is related to the study agent. Relatedness will be categorized according to the Manual for Expedited Reporting of Adverse Events to DAIDS (Version 2.0, January 2010), as follows:

- *Related:* There is a reasonable possibility that the AE may be related to the study agent(s)
- *Not Related:* There is not a reasonable possibility that the AE is related to the study agent(s)

8.4 Expedited Adverse Event Reporting Requirements

8.4.1 Adverse Event Reporting to DAIDS

Requirements, definitions and methods for expedited adverse event (EAE) reporting are outlined in Version 2.0 of the Manual for Expedited Reporting of Adverse Events to DAIDS, which is available on the Regulatory Support Center (RSC) website at http://rsc.tech-res.com/safetyandpharmacovigilance/. For each study participant, EAE reporting will be undertaken throughout the scheduled duration of follow-up, i.e., from the time of random assignment through study termination.

The DAIDS Adverse Experience Reporting System (DAERS), an internet-based reporting system, must be used for EAE reporting to DAIDS. In the event of system outages or technical difficulties, EAEs may be submitted via the DAIDS EAE Form. For questions about DAERS, please contact DAIDS-ES at DAIDS-ESSupport@niaid.nih.gov. Site queries may also be sent from within the DAERS application itself.

Where DAERS has not been implemented, sites will submit EAEs by documenting the information on the current DAIDS EAE Form. This form is available on the RSC website, http://rsc.tech-res.com/safetyandpharmacovigilance/. For questions about EAE reporting, please contact the RSC, DAIDSRSCSafetyOffice@tech-res.com.

EAE reporting procedures specific to this protocol are that once the sites have submitted EAEs via DAERS (as above), the RSC Safety Office will prepare the draft safety reports and send them to the CONRAD and DAIDS MO for review.

Study sites will be contacted by the DAIDS MO if any further information or clarification is needed after the report is evaluated by CONRAD and DAIDS MO. The RSC Safety Office will then prepare the final report which will go to CONRAD for signature and submission to the FDA. Copies of this final report will be filed with CONRAD and RSC. Additionally, the RSC Safety Office will distribute safety reports to all DAIDS sites that use products under investigation in this study.

For all EAEs submitted, sites must file an RSC update with the final or stable outcome unless the initial EAE submitted had a final or stable outcome noted already.

8.4.2 Reporting Requirements for this Study

- The SAE Reporting Category, as defined in the Manual for Expedited Reporting of Adverse Events to DAIDS, Version 2.0, January 2010, will be used for this study

- The study agents for which expedited reporting are required are:
  - FTC/TDF tablet
  - TFV RG 1% gel (See SSP for expedited requirements for reporting to CONRAD)
  - Study gel applicator

- Study staff will also report on CRFs the following subset of AEs reported by or observed in enrolled participants:
  - All anorectal AEs Grade 1 and higher
  - All AEs of severity Grade 2 and higher

8.4.3 Grading Severity of Events

The grading of severity of events and the reporting period will be the same for all AEs, as described in Section 8.3.1.

8.4.4 Expedited AE Reporting Period

- The EAE reporting period for this study begins once the participant is randomized and continues up through study termination.

- After the protocol-defined AE reporting period, unless otherwise noted, only suspected, unexpected serious adverse reactions (SUSARs) as defined in Version 2.0 of the Manual for Expedited Reporting of Adverse Events to DAIDS will be reported to DAIDS if the study staff become aware of the events on a passive basis (from publicly available information)

8.5 Social Harms Reporting

Although study sites make every effort to protect participant privacy and confidentiality, it is possible that participants' involvement in the study could become known to others and that

social harms may result. Social harms that are judged by the IoR/designee to be serious or unexpected will be reported to the PSRT and responsible site IRBs according to their individual requirements.

8.6 Regulatory Requirements

Information on all reported AEs will be included in reports to the US FDA and other government and regulatory authorities, as applicable. Site IoRs/designees will submit AE information in accordance with local regulatory requirements. This reporting will include site IRB/EC-mandated reporting of AEs, SAEs and any other relevant safety information.

9 CLINICAL MANAGEMENT

Guidelines for clinical management and temporary product hold/permanent discontinuation of study product are outlined in this section. In general, the IoR/designee has the discretion to hold study product temporarily at any time if s/he feels that continued product use would be harmful to the participant or interfere with treatment deemed clinically necessary. Unless otherwise specified below, the IoR/designee should immediately notify the PSRT upon initiation of a temporary product hold and consult the PSRT for further guidance on resuming study product, continuing the hold temporarily, or progressing to permanent discontinuation of study product. The IoR/designee will document all temporary product holds and permanent discontinuations on applicable CRFs.

9.1 Grading System

AE severity grading is described in Section 8.3.1.

9.2 Dose Modification Instructions

No dose modifications will be undertaken in this study.

9.3 General Criteria for Temporary Hold and/or Permanent Discontinuation of Study Product

- Acquisition of HIV-1 infection (permanent discontinuation); study product should be held beginning immediately upon recognition of the first reactive immunoassay. If via the algorithm in Appendix II the participant is determined to be HIV-uninfected, the participant may resume product use.

- Report of use of PEP for HIV exposure (permanent discontinuation)

- Hepatitis B infection (permanent discontinuation)

- Participant is unable or unwilling to comply with required study procedures, or otherwise might be put at undue risk by continuing product use, according to the judgment of the

IoR/designee (temporary hold). The IoR/designee must consult the PSRT on all temporary product holds instituted for this reason for further guidance on resuming product use, continuing the temporary hold, or progressing to permanent discontinuation.

9.4 Temporary Product Hold/Permanent Discontinuation in Response to Adverse Events

AE severity grading is described in Section 8.3.1.

Grade 1 or 2
In general, a participant who develops a Grade 1 or 2 AE, regardless of relationship to study product may continue product use. If the IoR opts to temporarily hold study product, the PSRT must be notified.

Grade 3
Participants who develop a Grade 3 AE that is judged by the IoR/designee to be unrelated to study product may continue product use, but must immediately consult the PSRT regarding further management.

For participants who develop a Grade 3 AE that is judged by the IoR/designee to be related to product, study product must be temporarily held until the PSRT can be consulted regarding potential resumption or other further management.

If a recurrence of the same Grade 3 AE judged to be related to study product recurs at any time during the study, study product must be temporarily held until the PSRT can be consulted regarding permanent discontinuation or other further management.

Grade 4
A participant who develops a Grade 4 AE, regardless of relationship to study product, will have the study product temporarily held. The IoR/designee must consult the PSRT and continue the temporary product hold until a recommendation is obtained from the PSRT. If, in consultation with the PSRT, product use is resumed and the same AE recurs at Grade 4 level at any time during the study, study product must then be permanently discontinued.

9.5 Criteria for Early Termination of Study Participation

Participants may voluntarily withdraw from the study for any reason at any time. The IoR also may withdraw participants from the study to protect their safety and/or if they are unwilling or unable to comply with required study procedures, after consultation with the PSRT. Participants also may be withdrawn if CONRAD, Gilead Sciences, Inc., government or regulatory authorities, including the FDA and Office for Human Research Protections (OHRP), or site IRBs/ECs terminate the study prior to its planned end date. Every reasonable effort is made to complete a final evaluation of participants who withdraw or are withdrawn from the study prior to completing follow-up. Study staff members will record the reason(s) for all withdrawals in participants' study records. If, after consultation with the PSRT, participants who voluntarily withdraw from the study wish to re-join the study, they may resume product use (if applicable) and follow-up through their originally scheduled study exit date.

9.6 HIV-1 Infection

The MTN-017 study is designed to minimize potential risks to participants who may become infected with HIV, with particular attention to minimizing potential exposure to HIV mono or dual therapy, with routine monthly testing for HIV. The informed consent process includes a thorough discussion of potential risks of study product exposure in HIV-infected participants, including the potential for selection of drug-resistant virus. The eligibility criteria exclude men and transgender females with known HIV-1 infection. Routine monitoring for HIV-1 infection takes place at monthly visits to minimize the potential for HIV-infected men and transgender females to continue taking study products if they develop HIV infection.

Participants identified as infected with HIV are managed or referred for management according to the local standard of care. Written SOPs for referral for HIV-1 care and treatment are in place at each study site. Study site investigators have identified facilities offering psychological and social services and medical care, including antiretroviral therapy (ART), to people infected with HIV-1 in the study countries. Some of the research sites are part of health care institutions that provide HIV-1 care and support, and can refer participants to those services.

The level of care provided at the referral sites is at a level that meets or exceeds the community standard for HIV-1 care. At every study visit, study staff will actively follow-up to see if the participant sought care. Additional counseling also may be needed to help ensure the participant receives appropriate care. All follow-up actions, outcomes, counseling, and plans for next steps are documented in participant study records. Results of study laboratory testing may be helpful in clinical management; these results are provided to the participant and his medical provider in real-time.

9.7 Hepatitis B Infection

MTN-017 will be carried out in countries with high endemic rates of HBV infection. While TDF has potent activity against HBV, severe acute exacerbations of hepatitis B have been reported in patients who are coinfected with HBV and HIV and have discontinued TDF. Although transaminitis associated with ongoing TDF use is uncommon, it has been reported. For these reasons, several mechanisms for protecting participants against AEs associated with TDF use, particularly in the setting of pre-existing or newly acquired HBV infection, are planned for the MTN-017 study.

First, all participants undergo screening for active HBV with assessment of hepatitis B surface antigen (HBsAg) at the screening visit. Those with active HBV infection receive standardized counseling relevant to natural history and transmission risks of HBV, and are excluded from enrollment. Those who test negative for HBsAg are offered immunization against HBV and are considered eligible for enrollment. Participants who decline HBV immunization are also eligible for enrollment.

Participants who decline immunization for HBV, and thus remain vulnerable to HBV infection over the course of the trial, will be offered HBV vaccination at all study visits. During follow-up, HBV serology may be drawn for suspected HBV infection at the discretion of the investigator of record or designee. Those participants with newly detected HBsAg will have study product

discontinued and will be followed monthly for an additional three months with transaminases to ensure that post-cessation hepatitis flares are diagnosed and managed appropriately.

9.8 Genital Sexually Transmitted Infection/Reproductive Tract Infection

The IoR/designee should manage STI/RTI per current WHO guidelines, available at http://www.who.int/en/. Observed single oral dose treatment should be provided whenever possible.

The study product need not be held in the event of an STI/RTI requiring treatment, unless other temporary product hold/permanent discontinuation guidelines apply. Should the IoR/designee determine that a temporary product hold is warranted, consultation with the PSRT is required.

10 STATISTICAL CONSIDERATIONS

10.1 Overview and Summary of Design

This is a phase 2, multi-site, randomized, six-sequence, three-period, open-label, crossover study. All the enrolled participants will use all three treatment regimens (i.e., daily FTC/TDF tablet, daily TFV RG 1% gel, and RAI-associated TFV RG 1% gel). Study participants will be randomized to the six different sequences of treatment regimen. The total length of follow-up is approximately 27 weeks or 189 days, which includes 24 weeks (168 days) of product use, or three eight week on-treatment periods, where treatment periods 1 and 2 will be followed by a one-week washout period and Period 3 will be followed by a one-week follow-up period. Each one-week period will be used to collect data on adverse events that may have resulted from study product use and/or procedures that occurred during the previous period.

10.2 Study Endpoints

Primary Endpoints

- **Safety:**
  - o Grade 2 or higher adverse events

- **Acceptability:**
  - o Participant self-report of ease of use, liking the product, and likelihood of product use if shown to be effective

Secondary Endpoints

- **Pharmacokinetics:**
  - o Tenofovir concentrations in blood plasma, rectal tissue and rectal fluid
  - o Tenofovir-diphosphate concentrations in PBMC and rectal tissue
  - o Emtricitabine concentrations in blood plasma, rectal tissue and rectal fluid
  - o Emtricitabine-triphosphate concentrations in PBMC and rectal tissue

Note: Rectal tissue will be collected on a subset of participants taking part in the Rectal Biopsy/Fluid Subset

- **Adherence:**
  - o Percentage of prescribed doses taken orally or administered rectally in an 8-week period

Exploratory Endpoints

- **Pharmacodynamics**:
  - o Determination of HIV replication in rectal tissue and anti-HIV activity from rectal fluid, at sites with capacity

- **Mucosal Immunity:**
  - o Mediators of mucosal immunity at enrollment and at the end of each study period, at sites with capacity

- **Correlation between PK and adherence:**
  - o PK measurements will be compared with adherence measures

- **Factors associated with adherence:**
  - o Identify associations between number of pills and applicators used during the 8-week cycles and acceptability, demographic, and background factors

- **Sexual activity and condom use:**
  - o Participant self-reported frequency of sexual activity and condom use during the trial

- **Product sharing:**
  - o Participant self-reported sharing of study product, quantity of shared study product, and with whom it was shared

- **Problem practices:**
  - o Practices associated with anal intercourse that may affect microbicide use

10.3 Primary Study Hypotheses

The study hypotheses for the primary objectives are:

- **Safety:** Daily FTC/TDF tablet (oral) and rectally-administered TFV RG 1% gel (dosed daily and RAI-dependent) will be generally safe and well-tolerated.

- **Acceptability:** Daily FTC/TDF tablet (oral) and rectally-administered TFV RG 1% gel (dosed daily and RAI-dependent) will be acceptable to at-risk HIV-uninfected males and transgender females who practice receptive anal intercourse.

10.4 Sample Size and Power Calculations

Sample size/power formulas for a parallel design (i.e., independent groups of participants on each treatment regimen) can be used to compute sample size/power. The sample size resulting from the assumption of independent groups can then be adjusted to reflect that there will be intra-participant correlation in the crossover study design. This sample size adjustment is obtained using the formula:

$N' = N(1-\rho)/2$

where N' is the sample size for the crossover study, N is the total number of participants necessary for a parallel design with two arms (N/2 in each arm) and ρ is the correlation between responses within a single participant during different treatment periods (intra-participant correlation).

To our knowledge, there are no data available to estimate the intra-participant correlation in this population of participants for any of the outcomes. However, for all outcomes it is highly likely that this correlation will be positive and large.

10.4.1 Primary Endpoints

Safety

Based on previous studies we expect to observe rates of the primary safety endpoints between 20% to 50%. Table 12 shows the minimum detectable difference in rates of safety events assuming 80% power, α=0.05, a two-sided test based on the Normal approximation of the Binomial distribution, varying rates of safety events in the treatment regimen with the lower rate, varying values of ρ (the intra-participant correlation), a sample size of 186 participants and 5% loss to follow-up (a working sample size of 176). The 5% loss to follow-up is based upon previously completed studies, MTN-007[28] and RMP 002/MTN-006[15] where loss-to-follow up rates were as follows: 4 of 65 (6%), 0 of 18 (0%), respectively. (Data on file)

Table 12: Minimum Detectable Differences in Rates of Safety Outcomes Assuming 80% Power

	P			
	0.0	0.3	0.5	0.7
Rate of Safety Event in Treatment Regimen with Lower Rate	Minimum Detectable Difference Between Treatment Regimens			
20%	13.3%	11.1%	9.4%	7.3%
50%	14.7%	12.3%	10.4%	8.1%

If there is no intra-participant correlation for safety outcomes, the study will have 80% power to detect a minimum difference of 13.3% to 14.7% depending on the rate of the safety event in the treatment regimen with the lower rate. If the intra-participant correlation is moderately high (0.5) this minimum detectable difference ranges from 9.4% to 10.4%.

Acceptability

Based on previous studies we expect to observe a high rate of acceptability (>80%) which equates to ≤20% of participants reporting they would be "unlikely" to use the study product in

the future. The table. below shows the minimum detectable difference in rates of 1-acceptability assuming 80% power, α=0.05, a two-sided test based on the Normal approximation of the Binomial distribution, varying rates of 1-acceptability in the treatment regimen with the lower rate, varying values of ρ (the intra-participant correlation), a sample size of 186 participants, and 5% loss to follow-up (a working sample size of 176).

Table 13: Minimum Detectable Difference in Rates of Acceptability Assuming 80% Power

	P			
	0.0	0.3	0.5	0.7
1-Acceptability Rate in the Treatment Regimen with the Lower Rate of 1-Acceptability	Minimum Detectable Difference Between Treatment Regimens			
5%	8.7%	7.3%	6.2%	4.8%
10%	10.7%	9.0%	7.6%	5.9%
15%	12.1%	10.1%	8.6%	6.6%
20%	13.3%	11.1%	9.4%	7.3%

If there is no intra-participant correlation for acceptability, the study will have 80% power to detect a minimum difference of 8.7% to 13.3% depending on the rate of acceptability in the treatment regimen with the lower rate. If the intra-participant correlation is moderately high (0.5) this minimum detectable difference ranges from 6.2% to 9.4%.

10.4.2 Secondary Endpoints

Pharmacokinetics

For PK endpoints, steady-state drug concentrations will be determined at each of 3 end-of-period visits. Samples to be collected will include blood plasma (TFV, FTC), PBMC (TFV-DP, FTC-TP), rectal tissue (TFV, FTC, TFV-DP, FTC-TP), and rectal fluid (TFV, FTC). Time of the prior dose will be recorded to inform the PK analysis. Data will be compared between dosing arms to determine steady-state drug concentration differences between study products.

Product Adherence

Based on previous studies we expect to observe a high rate of participants reporting taking at least 90% of expected doses. Table 14 shows the minimum detectable difference in rates of >90% adherence assuming 80% power, α=0.05, a two-sided test based on the Normal approximation of the Binomial distribution, varying rates of >90% adherence in the treatment regimen with the lower rate, varying values of ρ (the intra-participant correlation), and a sample size of 186 participants, and 5% loss to follow-up (a working sample size of 176).

Table 14: Minimum Detectable Difference in Rates of >90% Adherence Assuming 80% Power

	P			
	0.0	0.3	0.5	0.7
>90% Adherence Rate in the Treatment Regimen with the Lower Rate	Minimum Detectable Difference Between Treatment Regimens			
60%	14.0%	11.7%	9.9%	7.7%
70%	12.7%	10.6%	9.0%	7.0%
80%	10.5%	8.8%	7.4%	5.8%
90%	7.3%	6.1%	5.2%	4.0%

If there is no intra-participant correlation for adherence, the study will have 80% power to detect a minimum difference of 7.3% to 14.0% depending on the rate of adherence in the treatment regimen with the lower rate. If the intra-participant correlation is moderately high (0.5) this minimum detectable difference ranges from 5.2% to 9.9%.

10.5 Participant Accrual, Follow-up and Retention

The accrual period will be approximately 6-9 months at each site. Approximately 186 participants will be enrolled.

Each participant will be followed for approximately 27 consecutive weeks (three periods of 8 weeks with a washout period of 1 week between Period 1 and Period 2, a washout period of 1 week between Period 2 and Period 3, plus an additional week beyond the Period 3 End Visit to collect any new or worsening AEs). Study termination is defined as the 7th day after the Period 3 Final Clinic Visit date. For participants who terminate early from the study, their termination date is considered the date the Early Termination Visit is completed, or the date the participants considered no longer in the study. In a crossover study, it is important to have completeness of the data such that the target retention should be set at 100%. Therefore, once a participant has enrolled in the study, the study site will make every reasonable effort to retain the participant for the entire study period so that the participant is evaluable. A maximum of 5% loss-to-follow-up of enrolled participants is targeted.

Note: Replacement participants will be considered in consultation with the SMC if loss to follow-up is higher than expected.

10.6 Randomization

Randomization to product sequence will be stratified by site. Within each site, participants will be randomly assigned to one of the six study sequences outlined in Table 15 below. In an unblinded trial, special care needs to be taken to assure that the study staff cannot control or guess assignment. The MTN Statistical and Data Management Center will coordinate the randomization procedures, which will be specified in the SSP manual.

Table 15: Study Regimen

Sequence	**Period 1** (8 weeks)	Washout (~1 week)	**Period 2** (8 weeks)	Washout (~1 week)	**Period 3** (8 weeks)	**Follow-up** (~1 Week)
1	Oral (Daily FTC/TDF)		Rectal (Daily TFV RG 1% gel)		Rectal (RAI-associated TFV RG 1% gel)	
2	Rectal (RAI-associated TFV RG 1% gel)		Oral (Daily FTC/TDF)		Rectal (Daily TFV RG 1% gel)	
3	Rectal (Daily TFV RG 1% gel)		Rectal (RAI-associated TFV RG 1% gel)		Oral (Daily FTC/TDF)	
4	Rectal (Daily TFV RG 1% gel)		Oral (Daily FTC/TDF)		Rectal (RAI-associated TFV RG 1% gel)	
5	Oral (Daily FTC/TDF)		Rectal (RAI-associated TFV RG 1% gel)		Rectal (Daily TFV RG 1% gel)	
6	Rectal (RAI-associated TFV RG 1% gel)		Rectal (Daily TFV RG 1% gel)		Oral (Daily FTC/TDF)	

10.7 Data and Safety Monitoring and Analysis

10.7.1 Study Monitoring Committee

No Data and Safety Monitoring Board oversight is planned for this study. The MTN SMC will conduct interim reviews of study progress, including rates of participant accrual, retention, completion of primary and main secondary endpoint assessments, and study or lab issues. These reviews will take place approximately every 4-6 months, or as needed. At the time of these reviews, or at any other time, the SMC may recommend that the study proceed as designed, proceed with design modifications, or be discontinued. Safety monitoring will be done by the Protocol Safety Review Team.

10.7.2 Primary Analysis

All primary analyses will be based on data from the participants with data in all three periods of study (participants with at least one follow-up visit in each period of the study). Baseline values between these participants and those participants lost to follow-up will be described and used to interpret the generalizability of the results. Additionally, secondary analyses will analyze endpoints among all participants (evaluable and lost to follow-up) in only the first period of follow-up to describe the sensitivity of the results to loss to follow-up of participants.

Conditional logistic regression models controlling for period and sequence will be used to compare the three product regimens for the safety and adherence endpoints. In the event that participants have more than one safety event per period of treatment regimen a Generalized Estimating Equation model with a Poisson (log) link, an offset of the number of visits per treatment period, an exchangeable correlation structure, and robust errors will be used.

10.7.3 Secondary Analyses

All secondary analyses will be based on data from the participants with data in all three periods of study (participants with at least one follow-up visit in each period of the study). Baseline values between these participants and those participants lost to follow-up will be described and used to interpret the generalizability of the results. Additionally, secondary analyses will analyze endpoints among all participants (evaluable and lost to follow-up) in only the first period of follow-up to describe the sensitivity of the results to loss to follow-up of participants.

Conditional logistic regression models controlling for period and sequence will be used to compare the three product regimens for binary outcomes and a mixed effects models will be used to compare the three product regimens for continuous outcomes. The mixed effects models will include fixed effects for product regimen, period, and sequence and random effects for participant within sequence. If the continuous outcome data are not Normally distributed, prior to formal analyses transformations to achieve Normality will be explored as well as categorization of the outcome data and alternative models appropriate for categorical data will be used.

10.7.4 Missing Data

We expect little to no missing data. Data will be considered missing (no data on outcome measures) if a participant does not return for a follow-up visit. However, if the probability of missing measurements depends on either covariates or on the measurement outcomes of participants, then the methods described above may give biased inferences and point estimates. If a substantial amount of endpoint data is missing (e.g., follow-up data missing in at least 10% of participants), then secondary analyses of the endpoints will be conducted using methods that relax the missing completely at random assumption to a missing at random assumption. For a univariate binary and quantitative outcome, respectively, a generalized linear model with a binomial or normal error distribution will be used for estimation and testing.

11 DATA HANDLING AND RECORDKEEPING

11.1 Data Management Responsibilities

CRFs will be developed by the MTN SDMC in conjunction with the protocol team. Quality control reports and queries routinely will be generated and distributed by the SDMC to the study sites for verification and resolution. As part of the study activation process, each study site must identify all CRFs to be used as source documents. Data are transferred to the MTN SDMC, entered, and cleaned using the DataFax data management system.

11.2 Source Documents and Access to Source Data/Documents

All study sites will maintain source data/documents in accordance with current DAIDS policies. (http://www.niaid.nih.gov/labsandresources/resources/daidsclinrsrch/Pages/Default.aspx)

Each IoR/designee will maintain, and store securely, complete, accurate and current study records throughout the study. In accordance with U.S. regulations regarding testing investigational products, the IoR/designee will maintain all study documentation for at least two years following the date of marketing approval for the study product being tested for the indication in which they were studied. If no marketing application is filed, or if the application is not approved, the records will be retained for two years after the investigation is discontinued and the US FDA is notified.

Study records must be maintained on site for the entire period of study implementation. Thereafter, instructions for record storage will be provided by DAIDS. No study records, including audio recordings, may be moved to an off-site location or destroyed prior to receiving approval from DAIDS.

11.3 Quality Control and Quality Assurance

All study sites will conduct quality control and quality assurance procedures in accordance with current DAIDS policies.
(http://www.niaid.nih.gov/labsandresources/resources/daidsclinrsrch/documents/qmppolicy.pdf)

12 CLINICAL SITE MONITORING

Study monitoring will be carried out by Pharmaceutical Product Development, Inc. (PPD) (Wilmington, NC) in accordance with current DAIDS policies. Study monitors will visit the site to do the following:

- Review informed consent forms, procedures, and documentation
- Assess compliance with the study protocol, GCP guidelines, and applicable regulatory requirements (US and non-US), including CFR Title 45 Part 46 and Title 21 Parts 50, 56, and 312
- Perform source document verification to ensure the accuracy and completeness of study data
- Verify proper collection and storage of biological specimens
- Verify proper storage, dispensing, and accountability of investigational study products
- Assess implementation and documentation of internal site quality management procedures

The IoR/designee will allow study monitors to inspect study facilities and documentation (e.g., informed consent forms, clinic and laboratory records, other source documents, CRFs), as well as observe the performance of study procedures. The IoR/designee also will allow inspection of all study-related documentation by authorized representatives of the MTN CORE, SDMC, NL, CONRAD, Gilead Sciences, Inc., NIAID, FDA, OHRP and local and US regulatory authorities. A site visit log will be maintained at the study site to document all visits.

13 HUMAN SUBJECTS PROTECTIONS

13.1 Institutional Review Boards/Ethics Committees

Site investigators will make efforts to minimize risks to participants. Participants and study staff members will take part in a thorough informed consent process. Before beginning the study, the IoR will have obtained IRB approval and the protocol will have been submitted to the FDA. The IoR will permit audits by authorized representatives of the MTN CORE, SDMC, NL, CONRAD, Gilead Sciences, Inc., NIAID, FDA, OHRP and local and US regulatory authorities.

13.2 Protocol Registration

Prior to implementation of this protocol, and any subsequent full version amendments, each site must have the protocol and the protocol consent forms approved, as appropriate, by their local IRB and any other applicable regulatory entity (RE). Upon receiving final approval, sites will submit all required protocol registration documents to the DAIDS Protocol Registration Office (DAIDS PRO) at the RSC. The DAIDS PRO will review the submitted protocol registration packet to ensure that all of the required documents have been received.

Site-specific informed consent forms (ICFs) *will not* be reviewed or approved by the DAIDS PRO, and sites will receive an Initial Registration Notification when the DAIDS PRO receives a complete registration packet. Receipt of an Initial Registration Notification indicates successful completion of the protocol registration process. Sites will not receive any additional notifications from the DAIDS PRO for the initial protocol registration. A copy of the Initial Registration Notification should be retained in the site's regulatory files.

Upon receiving final IRB/Ethics Committee (EC) and any other applicable RE approval(s) for an amendment, sites should implement the amendment immediately. Sites are required to submit an amendment registration packet to the DAIDS PRO at the RSC. The DAIDS PRO will review the submitted protocol registration packet to ensure that all the required documents have been received. Site-specific ICF(s) *will not* be reviewed and approved by the DAIDS PRO and sites will receive an Amendment Registration Notification when the DAIDS PRO receives a complete registration packet. A copy of the Amendment Registration Notification should be retained in the site's regulatory files.

For additional information on the protocol registration process and specific documents required for initial and amendment registrations, refer to the current version of the DAIDS Protocol Registration Manual.

13.3 Study Coordination

CONRAD holds an Investigational New Drug (IND) application for this study. Copies of all regulatory documents submitted to this IND by CONRAD are forwarded to DAIDS for cross-referencing with other INDs for the study products. Assignment of all sponsor responsibilities for this study will be specified in Clinical Trial Agreements (CTA) executed by NIAID and CONRAD, and Gilead Sciences, Inc.

Study implementation will also be guided by a common study-specific procedures manual that provides further instructions and operational guidance on conducting study visits; data and forms processing; specimen collection, processing, and shipping; AE assessment, management and reporting; dispensing study products and documenting product accountability; and other study operations. Standardized study-specific training will be provided to all sites by the MTN CORE, SDMC, NL and other designated members of the Protocol Team.

Close coordination between protocol team members is necessary to track study progress, respond to queries about proper study implementation, and address other issues in a timely manner. The PSRT will address issues related to study eligibility and AE management and reporting as needed to assure consistent case management, documentation, and information-sharing across sites. Rates of accrual, adherence, follow-up, and AE incidence will be monitored closely by the team as well as the SMC.

13.4 Risk Benefit Statement

13.4.1 Risks

General
It is not expected that this trial will expose human subjects to unreasonable risk.

Phlebotomy may lead to discomfort, feelings of dizziness or faintness, and/or bruising, swelling and/or infection.

Insertion of a lubricated anoscope will likely cause some discomfort

There is the risk of mild discomfort in addition to a slight risk of bleeding with the insertion of rectal swabs and sponges.

Use of an applicator to deliver a microbicide into the rectal compartment may be associated with minor anorectal trauma including lacerations and bruising in the anorectal area.

Disclosure of HIV and STI status may cause worry, sadness or depression. Disclosure of HIV-positive status has been associated with depression, suicidal ideation, and denial as well as social isolation. Trained counselors will be available to help participants deal with these feelings. Participation in clinical research includes the risks of loss of confidentiality and discomfort with the personal nature of questions when discussing sexual behaviors.

Participants at sites requiring partner or local health authority notification in response to diagnosed STI or HIV infection could have problems in their relationships with their sexual partners. Participants also could have problems in their partner relationships associated with use or attempted use of study gel.

Site staff will make every effort to protect participant privacy while in the study. Although study sites make every effort to protect participant privacy and confidentiality, it is possible that participants' involvement in the study could become known to others, and that social harms may result (i.e., because participants could become known as or at "high risk" for HIV infection). For

example, participants could be treated unfairly or discriminated against, or could have problems being accepted by their families and/or communities.

A subset of participants will have a flexible sigmoidoscopy. A flexible sigmoidoscopy is a commonly practiced medical procedure. The procedures done in this study will not involve any increased risk over usual flexible sigmoidoscopy performed for clinical indications. The risks associated with these procedures include mild discomfort and the feeling of having a “bloated stomach” as well as flatulence following the procedure. Endoscopic biopsies are painless and heal quickly within 3 days. On extremely rare occasions, the endoscopic procedure or biopsies may lead to pain, infection (sepsis), bleeding or perforation of the gastrointestinal tract. Perforation occurs approximately once out of every 1,000 procedures. If this extremely rare complication occurs, antibiotics and surgery to repair the tear may be necessary.

A subset of participants will also have an enema. A hollow tube about the thickness of a pencil will be used to put approximately 125 mL of saline (salt water) into the rectum and cleanse the bowel of fecal matter. An enema may be standard procedure prior to insertion of an anoscope or flexible sigmoidoscope since fecal matter can obscure the test. This procedure may be repeated if the initial enema does not produce intended results. The main risk from having an enema is temporary discomfort. This may cause a “bloated” or “crampy” feeling. Some air may be pumped into the rectum as well, causing flatulence. The tube is small, but it might cause some anal or rectal discomfort if the subject has any hemorrhoids or other painful conditions.

General Disclaimer

The drugs used in this study may have side effects, some of which are listed below. Please note that these lists do not include all the side effects seen with these drugs. These lists include the more serious or common side effects with a known or possible relationship. If you have questions concerning additional study drug side effects please ask the medical staff at your site.

Use of Combination Antiretroviral Drugs

Immune Reconstitution Syndrome:
In some people with advanced HIV infection, symptoms from other infections or certain diseases may occur soon after starting combination anti-HIV treatment but can also occur later. Some of these symptoms may be life threatening. If you start having new symptoms, or notice that existing symptoms are getting worse after starting your antiretroviral therapy, tell your healthcare provider right away.

The use of potent antiretroviral drug combinations may be associated with an abnormal placement of body fat and wasting. Some of the body changes include:

- Increase in fat around the waist and stomach area
- Increase in fat on the back of the neck
- Thinning of the face, legs and arms
- Breast enlargement

Nucleoside Analogue
Lactic acidosis (elevated lactic acid levels in the blood) and severe hepatomegaly (enlarged liver) with steatosis (fatty liver) that may result in liver failure, other complications or death have

been reported with the use of antiretroviral nucleoside analogues alone or in combination. The liver complications and death have been seen more often in women on these drug regimens. Some nonspecific symptoms that might indicate lactic acidosis include: unexplained weight loss, stomach discomfort, nausea, vomiting, fatigue, cramps, muscle pain, weakness, dizziness and shortness of breath.

Risks associated with FTC/TDF

The following side effects have been associated with the use of **emtricitabine**:
- Headache
- Dizziness
- Tiredness
- Inability to sleep, unusual dreams
- Loose or watery stools
- Upset stomach (nausea) or vomiting
- Abdominal pain
- Rash, itching, which sometimes can be a sign of an allergic reaction
- Skin darkening of the palms and/or soles
- Increased cough
- Runny nose
- Abnormal liver function tests, which could mean liver damage
- Increases in pancreatic enzyme (substances in the blood), which could mean a problem with the pancreas
- Increased triglycerides
- Increased creatine phosphokinase, which could mean muscle damage

The following side effects have been associated with the use of **tenofovir**:
- Upset stomach, vomiting, gas, loose or watery stools
- Generalized weakness
- Dizziness
- Depression
- Headache
- Abdominal pain
- Worsening or new kidney damage or failure
- Inflammation or swelling and possible damage to the pancreas and liver
- Shortness of breath
- Rash
- Allergic reaction: symptoms may include fever, rash, upset stomach, vomiting, loose or watery stools, abdominal pain, achiness, shortness of breath, a general feeling of illness or a potentially serious swelling of the face, lips, and/or tongue
- Bone pain and bone changes such as thinning and softening which may increase the risk of breakage
- Muscle pain and muscle weakness

NOTE: If participants become infected with Hepatitis B, they should be aware that their liver function tests may increase, and symptoms associated with hepatitis (an acute inflammation of the liver) may worsen if the drug tenofovir or emtricitabine is stopped.

Risks associated with Tenofovir 1% Gel

Tenofovir 1% gel has been generally well-tolerated when used topically in the vagina and rectum.

The following side effects have been associated with the use of TFV 1% gel-

Penile application of TFV 1% gel (original vaginal formulation):

- Mild pain (burning, irritation, discomfort)
- Pruritus of the penis when exposed to gel

Rectal application of TFV 1% gel or TFV RG 1% gel:

- Mild rectal fullness
- Incontinence or diarrhea
- Flatulence
- Mild abdominal pain
- Proctalgia

Some of the possible side effects of TFV RG 1% gel:

- Dryness, itching, burning or pain in the genital area.

13.4.2 Benefits

Participants and others may have future benefit from information learned from this study. Specifically, information learned in this study may lead to the development of safe and effective interventions to prevent HIV transmission. Participants also may appreciate the opportunity to contribute to the field of HIV prevention.

Participants will receive HIV/STI risk reduction counseling, HIV and STI testing, physical exams, and routine laboratory testing related to blood, liver, and kidney function. Participants will be provided or referred for STI treatment as needed. For other medical conditions identified during the study, participants will be referred to local health care services. Some participants may benefit from earlier diagnosis and treatment of medical conditions identified during the study.

13.5 Informed Consent Process

Written informed consent will be obtained from each participant prior to initiation of study procedures. Written informed consent also will be obtained for long-term specimen storage and possible future testing, although consent for specimen storage is not required for study participation. In obtaining and documenting informed consent, the IoR and their designees will comply with applicable local and US regulatory requirements and will adhere to GCP. Study staff must document the informed consent process in accordance with current DAIDS policies. Participants will be provided with copies of the informed consent forms if they are willing to receive them.

The informed consent process will cover all elements of informed consent required by research regulations. In addition, the process specifically will address the following topics of import to this study:

- The need to practice safe sex behaviors
- The randomization to study sequence
- The importance of participants in each study group to the success of the study
- The importance of adherence to the study visit and procedures schedule
- The potential medical risks of study participation (and what to do if such risks are experienced)
- The potential social harms associated with study participation (and what to do if such harms are experienced)
- The benefits of study participation
- The distinction between research and clinical care
- The right to withdraw from the study at any time

13.6 Participant Confidentiality

All study procedures will be conducted in private, and every effort will be made to protect participant privacy and confidentiality to the extent possible. Each study site will implement confidentiality protections that reflect the local study implementation plan and the input of study staff and community representatives to identify potential confidentiality issues and strategies to address them. In addition to local considerations, the protections described below will be implemented at all sites.

All study-related information will be stored securely at the study site. All participant information will be stored in locked areas with access limited to study staff. All laboratory specimens, study data collection, and administrative forms will be identified by coded number only to maintain participant confidentiality. All records that contain names or other personal identifiers, such as locator forms and informed consent forms, will be stored separately from study records identified by code number. All local databases will be secured with password protected access systems. All digital audio files will be stored on password-protected computers. Forms, lists, logbooks, appointment books, and any other listings that link participants' ID numbers to identifying information will be stored in a separate, locked file in an area with limited access. Participants' study information will not be released without their written permission, except as necessary for review, monitoring, and/or auditing by the following:

- US regulatory authorities, including representatives of the US Federal Government, including the US Food and Drug Administration (FDA), OHRP, NIH, and/or contractors of the NIH
- Representatives of CONRAD and/or Gilead Sciences, Inc.
- Representatives of the MTN CORE, SDMC, and/or NL
- Study staff
- Local regulatory authorities, including site IRBs/ ECs

After receiving appropriate approval, all study documents/data will be properly disposed of, including the proper destruction and/or deletion of paper files, electronic study data, electronic documents. Audio files will be transcribed and immediately destroyed following a transcription quality assurance check. A member of the MTN Behavioral Research Working Group (BRWG) or designee is responsible for ensuring that these files have been destroyed.

MTN has obtained a Certificate of Confidentiality from the US Department of Health and Human Services that is applicable for the US sites participating in this study. This Certificate protects study staff from being compelled to disclose study-related information by any US Federal, State or local civil, criminal, administrative, legislative or other proceedings. It thus serves to protect the identity and privacy of study participants.

13.7 Special Populations

13.7.1 Pregnant Females

MTN-017 will not enroll biologically female participants.

13.7.2 Children

The NIH has mandated that children be included in research trials when appropriate. This study meets “Justifications for Exclusion” criteria for younger children as set forth by the NIH. Specifically, “insufficient data are available in adults to judge potential risk in children” and “children should not be the initial group to be involved in research studies.” This study does not plan to enroll children under 18 years old.

13.8 Compensation

Pending IRB/EC approval, participants will be compensated for time and effort in this study, and/or be reimbursed for travel to study visits. Site-specific reimbursement amounts will be specified in the study informed consent forms of each individual site.

13.9 Communicable Disease Reporting

Study staff will comply with local requirements to report communicable diseases including HIV identified among study participants to health authorities. Participants will be made aware of reporting requirements during the informed consent process.

13.10 Access to HIV-related Care

13.10.1 HIV Counseling and Testing

Participant-centered HIV test-related counseling will be provided to all potential study participants who consent to undergo HIV screening to determine their eligibility for this study, and to all enrolled participants at each follow-up HIV testing time point. Testing will be performed in accordance with the algorithm in Appendix II. Counseling will be provided in accordance with standard HIV counseling policies and methods at each site and additionally will emphasize the unknown efficacy of the study products in preventing HIV infection. In accordance with the policies of the NIH, participants must receive their HIV test results to take part in this study. Condoms will be provided to participants throughout the duration of their participation.

13.10.2 Care for Participants Identified as HIV-Positive

Identified as HIV-Positive Prior to Enrollment
An individual who has been identified as infected with HIV will be managed or referred for management according to the local standard of care.

Identified as HIV-Positive While on Study Product
Please refer to Section 9.5 and 9.6 for further details. Should an individual test positive for HIV after enrollment, follow-up procedures will be performed as per Section 7.6.1.

13.11 Study Discontinuation

This study may be discontinued at any time by NIAID, the MTN, CONRAD, the US FDA, OHRP, other government or regulatory authorities, or site IRBs/ECs.

14 PUBLICATION POLICY

DAIDS/NIAID and MTN policies and a CTA between CONRAD, Gilead Sciences, Inc., and NIAID, will govern publication of the results of this study. Any presentation, abstract, or manuscript will be submitted by the investigator to the MTN Manuscript Review Committee, DAIDS/NIAID, National Institute of Mental Health (NIMH) and CONRAD for review prior to submission.

15 APPENDICES

APPENDIX I: SCHEDULE OF STUDY VISITS AND EVALUATIONS

	Visit 1<br><br>SCR	Visit 2<br><br>ENR Initiate Period 1	Visit 3<br><br>Mid Period 1	Visit 4 End Period 1 Visit	Visit 5 Initiate Period 2 Visit	Visit 6 Mid Period 2 Visit	Visit 7 Period 2 End Visit	Visit 8 Initiate Period 3 Visit	Visit 9 Mid Period 3 Visit	Visit 10 End Period 3 Visit/ Final Clinic/ Early Term
**ADMINISTRATIVE AND REGULATORY**										
Informed consent (SCR/ENR)	X									
Assign PTID	X									
Collect demographic data	X									
Locator information	X	X	X	X	X	X	X	X	X	X
Assess eligibility	X									
Confirm participant eligibility		X								
Provide reimbursement	X	X	X	X	X	X	X	X	X	X
Schedule next study visit/contact	X	X	X	X	X	X	X	X	X	*
Provide available test results	X	X	X	X	X	X	X	X	X	X
Randomization		X								
Provision of AE dial-in instructions										X
**BEHAVIORAL/COUNSELING**										
Baseline Behavioral Questionnaire (CASI)		X								
HIV pre-/post-test and HIV/STI risk reduction counseling	X	X	X	X	*	X	X	*	X	X
Protocol adherence counseling		X	X		X	X		X	X	
Product use instructions and adherence counseling		X	X		X	X		X	X	
Data Convergence Interview			X	X		X	X		X	X
Follow-up Behavioral Questionnaire (CASI)				X			X			X
In-depth phone interview (sub-set)				X						
Rectal biopsy/fluid procedural counseling <u>(Rectal biopsy/fluid subset only)</u>	X	X		X			X			X
**CLINICAL**										
Medical history	X	X	X	X	X	X	X	X	X	X
Physical exam	X	X	*	*	*	*	*	*	*	X
Rectal exam	X	X	X	X	X	X	X	X	X	X
Record/update AEs		X	X	X	X	X	X	X	X	X
Concomitant medications	X	X	X	X	X	X	X	X	X	X
Treat for UTI/RTI/STI or refer	*****	*****	*****	*****	*****	*****	*****	*****	*****	*****
Hepatitis B vaccination or decline of vaccination		*****	*****	*****	*****	*****	*****	*****	*****	*****
**LABORATORY**										
**URINE**										
Dipstick UA	X	*	*	*	*	*	*	*	*	*
NAAT for GC/CT	X	*	*	*	*	*	*	*	*	X
**BLOOD**										
CBC w/ diff and platelets	X	*	*	*	*	*	*	*	*	X
AST/ALT	X	*	*	*	*	*	*	*	*	X
Creatinine	X	*	*	X	*	*	X	*	*	X
Plasma for archive		X								
Plasma for storage				X			X			X
Blood for PK (PBMC and plasma)				X			X			X
Syphilis RPR	X	*	*	*	*	*	*	*	*	*
HIV-1 serology	X	X	X	X	*	X	X	*	X	X
HSV ½ serology	X									

HBsAg	X									
Hepatitis C antibody	X									
Coagulation (PT/INR)<br>(Rectal biopsy/fluid subset only)	X									
**RECTAL**										
HSV 1/2 detection	*	*	*	*	*	*	*	*	*	*
Rectal sponge for adherence PK			X	X	X	X	X	X	X	X
Rectal sponge for PD		X	X	X	X	X	X	X	X	X
Rectal sponge for mucosal immunology (Rectal biopsy/fluid subset only)		X		X			X			X
Rectal biopsies for PK (Rectal biopsy/fluid subset only)				X			X			X
Rectal biopsies for PD (Rectal biopsy/fluid subset only)		X		X			X			X
Rectal biopsies for mucosal immunology (Rectal biopsy/fluid subset only)		X		X			X			X
Anal HPV		X								
NAAT for GC/CT	X	X	*	X	*	*	X	*	*	X
**STUDY PRODUCT/SUPPLIES**										
Provision of study product		X	X		X	X		X	X	
Observe first dose and/or simulation of a first dose with SMS training		X			X			X		
Collect unused study product			X	X		X	X		X	X
Provision of male condoms	X	X	X	X	X	X	X	X	X	X
Provision of lubricant		*	*		*	*		*	*	

X= required,*= if indicated

NOTE: In addition to the procedures listed above, study staff will follow-up with participants via phone call 48-72 hours and again 14 days after Visit 2 (Enrollment), Visit 5 and Visit 8 to collect AEs. Sites will reference SOPs regarding participant reimbursement.

APPENDIX II: ALGORITHM FOR HIV ANTIBODY FOR SCREENING AND FOLLOW-UP

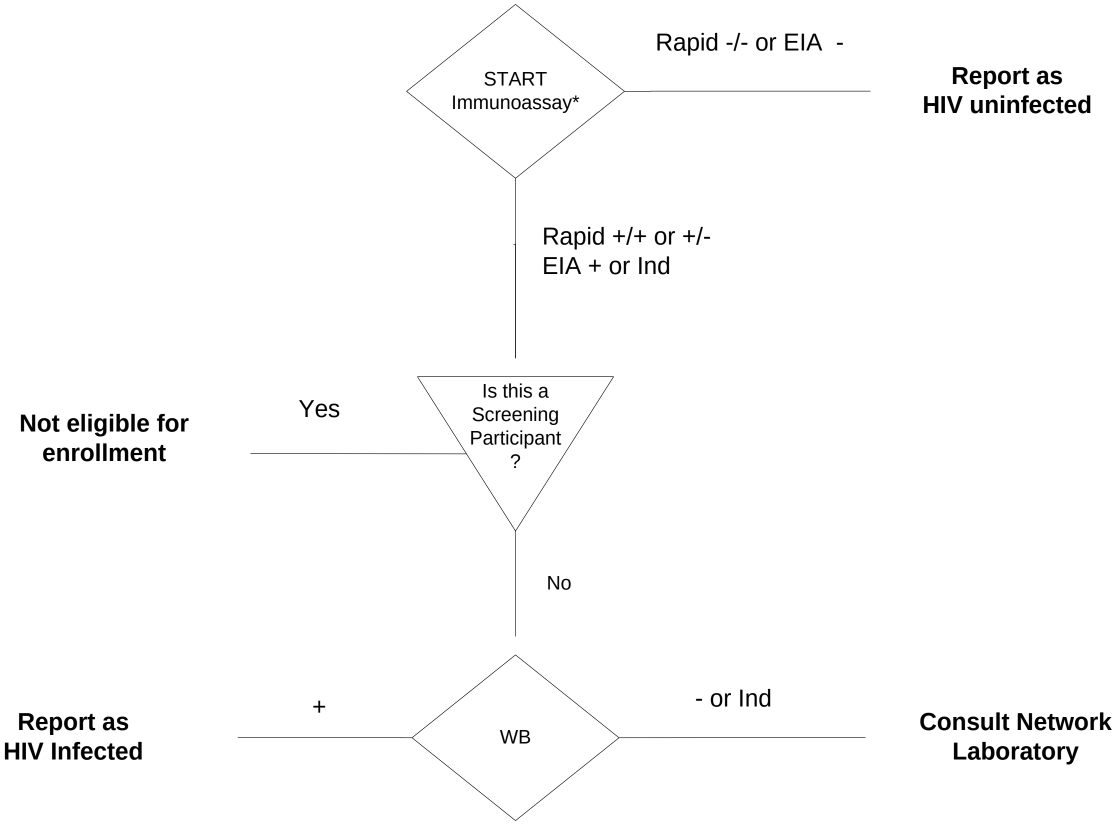

*CLIA certified labs may perform 1 rapid test
Ind: Indeterminate test results
EIA: Enzyme Immunoassay

APPENDIX III: SAMPLE INFORMED CONSENT FORM (SCREENING, ENROLLMENT, LONG-TERM STORAGE)

SAMPLE INFORMED CONSENT FORM
DIVISION OF AIDS, NIAID, NIH

MTN-017

A Phase 2 Randomized Sequence Open Label Expanded Safety and Acceptability Study of Oral Emtricitabine/Tenofovir Disoproxil Fumarate Tablet and Rectally-Applied Tenofovir Reduced-Glycerin 1% Gel

Version 1.0

July 17, 2012

PRINCIPAL INVESTIGATOR: [Sites to insert]
PHONE: [Sites to insert]
Short Title for the Study: Safety and acceptability study of oral FTC/TDF tablet and rectally-applied TFV RG 1% gel

INFORMED CONSENT
You are being asked to take part in this research study because you are a male or a transgender female (a male who identifies as a female), who is 18 years of age or older and reported at least one experience of receptive anal sex in the past 3 months. Approximately 186 men and transgender females will participate in this study across multiple sites in multiple countries. The United States National Institutes of Health (NIH) and CONRAD sponsor this Microbicide Trials Network (MTN) study. Gilead Sciences, Inc. also supplies product for this study. The person in charge of this study at this site is *[INSERT NAME OF PRINCIPAL INVESTIGATOR]*. Before you decide if you want to join this study, we want you to know about the study. This consent form gives you information about the study. The study staff will talk with you about it and answer your questions. Once you read and understand this study, you will be asked to sign this form if you agree to participate. You will be offered a copy of this form to keep. You may decide to stop being in the study at any time.

What happens if you do not want to take part?
Before you learn more about the study it is important that you understand:

- You do not have to be in this study if you do not want to.
- You can stop taking part at any time. This will not affect the service you get at this clinic
- If you decide to stop taking part, you may join another study, if we have one and you qualify.

Why is this research being done?
There are two main purposes of this study. The first is to compare the safety of two study products, a gel and an oral tablet. The gel contains a drug called tenofovir. The oral tablet, named Truvada®, contains two drugs, tenofovir and emtricitabine. Truvada® is licensed for treatment and prevention of HIV in adults and children. In July 2012, the US Food and Drug Administration approved Truvada® to be used for pre-exposure prophylaxis (PrEP) in combination with safer sex practices to reduce the risk of sexually-acquired HIV infection in adults at high risk. [Sites to modify and/or include language as appropriate.] We will see if tenofovir gel, (either used daily or before and after anal sex), is as safe as Truvada® taken daily. When the gel is used before and after anal sex no more than two doses should be applied within a 24 hour period (even if you have more than one episode of sex that day) and if you have a full week without anal sex, you will be asked to take 2 doses of gel on the last day of the week. Tenofovir gel will be inserted into the rectum with an applicator and Truvada® will be taken by mouth.

Another main purpose of this study is to find out how males and transgender females feel about using the study products.

Who will be in this research study and what will I be asked to do if I join?
There will be between up to 30 participants enrolled at each site. Approximately 186 participants will be enrolled in this study at many sites. Each participant will use the rectal gel daily for 8 weeks, use the rectal gel with receptive anal intercourse for 8 weeks, and take a daily tablet for 8 weeks. If you decide to take part in the study, the order in which you will use the products will be randomized (chosen “by lot” [or other equivalent local term, for example, throwing dice]). Neither you nor the study staff can choose or change the order in which you will use the products.

What will happen during study visits?

Screening Procedures –
Your first visit will happen today after you read, discuss, understand and sign this form to agree to participate. The procedures done at this visit will take about [Sites to insert time].

Study staff will:
- Ask you where you live and how we may contact you while you are taking part in this study, ask questions about your health (including what medications you are taking) and other questions to determine if you are eligible to participate in this study
- Perform a physical exam, measure your weight, take your temperature, pulse, blood pressure, and perform other procedures and perform other tests to better understand your health
- Perform a rectal exam. Rectal swabs will be taken, these will be used to test for infections passed through sex
- You will talk with study staff about HIV, HIV testing, and ways to prevent HIV and other infections passed through sex
- Take a blood sample [site to insert amount] to test for:
  - Hepatitis B and C
  - Health of your blood, liver and kidneys

- Infections passed through sex, including HIV. You will be told your HIV test result as soon as it is available [sites to add expected timeframe]. You will talk with the study staff about the meaning of your tests and feelings you may have about the results. Sometimes HIV tests are not clearly positive, but also not clearly negative. In that case, we will do more tests until we know your status for sure. You must receive HIV test results to be in the study. If the test shows that you have HIV, you cannot join the study. We will refer you to available sources of medical care and other services you may need. The study staff will tell you if there are other studies you may be eligible for.

- Ask you to provide a urine sample to test for infections
- Give you male condoms
- Give you treatment for infections other than HIV passed through sex and urinary tract infections, if you need treatment. This study does not provide care or treatment for HIV.
- Give you information for other services, if needed
- Schedule your next visit to enroll in MTN-017, if you are eligible

Results of tests listed above will be available within [*site to specify timeframe*] of your visit. The study staff will review your test results when they are available. You may return when the results are available. If the results show you are eligible to join the study, the study staff will review the study again with you and answer any questions you may have.

Enrollment and Follow-up Procedures –
If you are eligible, your next visit will be within thirty days of today's visit. That visit is the Enrollment/Initiate Period 1 Visit and you will begin using study product at that visit. After your screening visit, you will have the following study visits and phone calls:

PERIOD 1

- Visit 2: Enrollment Visit/ Initiate Period 1 (Day 0)
- Phone Call: Follow-up (48-72 hours after Visit 2)
- Phone Call: Follow-up (Week 2)
- Visit 3: Mid Period 1 Visit (Week 4)
- Visit 4: End Period 1 Visit (Week 8)

PERIOD 2

- Visit 5: Initiate Period 2 Visit (~Week 9)
- Phone Call: Follow-up (48-72 hours after Visit 5)
- Phone Call: Follow-up (Week 11)
- Visit 6: Mid Period 2 Visit (Week 13)
- Visit 7: Period 2 End Visit (Week 17)

PERIOD 3

- Visit 8: Initiate Period 3 Visit (Week 18)
- Phone Call: Follow-up (48-72 hours after Visit 8)
- Phone Call: Follow-up (Week 20)
- Visit 9: Mid Period 3 Visit (Week 22)
- Visit 10: Period 3 End Visit (Week 26)

You will be told the order in which you will use the products at the Enrollment Visit.

You will be asked to:
- Answer questions to confirm that you are able to join the study
- Agree to the rules of the study, such as not using certain rectal products for the length of your study participation. Study staff will provide you with a list of products that are not okay for you to use.

During the study, you will be asked to:
- Provide updated information about where you live and how to contact you
- Tell study staff if you have experienced any changes in your health, including changes to your medicine, or any problems related to the study since your last visit
- Answer questions about your behavior, sexual behavior, sexual behavior practices related to your gel use, and your use of the study products. Some of these questions may be asked by computer and by daily text messages. The study staff will show you how to use the computer and how to send and receive text messages. This information is private and it will not be placed in your medical file.
- Schedule your next visit or study contact

You will also receive counseling. You talk with study staff about:
- When and how to use the study gel and tablets
- How to follow the rules of the study
- Sexually transmitted infections (STIs), HIV, HIV/STI testing, and ways to prevent HIV and other infections passed through sex

The following clinical procedures will be performed:
- A physical exam
- At some visits a blood sample [sites to insert amount] will be taken to test:
  - The health of your blood, liver and kidneys
  - For infections passed through sex, including HIV
  - For how much of the study product is in your body.
- An extra sample of blood [site to insert amount] will be drawn at certain visits in case there is a question about your lab results
- A urine sample will be collected to test for infections
- A rectal exam will be performed at all visits. Rectal swabs/sponges will be taken and may be used to test:
  - For infections passed through sex
  - How your rectal fluids protect against HIV in the laboratory
  - For how much of the study product is in your rectal fluids

  To collect these samples the clinician and/or designee will need to insert a short hollow tube called an anoscope inside your rectum.

- If your screening tests show that you do not have immunity to hepatitis B (immunity means protection against infection), you will be offered the hepatitis B vaccine. If you choose to get hepatitis B vaccine, you will have this vaccine three times [sites to insert local guidelines, if applicable].

As part of the clinical procedures you will:

- Receive treatment or be referred for treatment for problems (including infections passed through sex) that the study staff may find. You will also be offered male condoms.
- Receive test results, when they are available

You will be asked to use study product; as part of using study product you will:

- Receive study gel or tablets to be used daily or study gel to be used when you have anal sex, and talk with study staff about how to take the tablets and how to correctly use gel. You will also receive lubricant to aid in using the gel applicator.
- Be asked to bring unused gel applicators and tablets with you to your visits so that they can be counted by study staff
- Be called by study staff so that you can report any health problems or other problems since your last visit. You can always call study staff if you have any problems related to the study product or your participation in this study.
- Answer a few short questions by cell phone via text messages that will ask about your use of the study product and sexual activity. You will be paid for each text message session. You will receive this questionnaire every day at a time that is convenient for you. The answers are confidential and will only be labeled with your study ID number.
- Review with the study staff your answers to the daily questions sent by text message, and the number of doses returned to the clinic, so that you can help the study staff to understand when and how you used the study products. These conversations will be audio-recorded to ensure the session is done correctly at all sites.
- Answer questions on a computer about your behavior, including your sexual behavior, and your experiences using the study product and participating in the study. You will answer questions on a computer during each study period. This information is confidential and WILL NOT be placed in your medical record. The questionnaire will only be labeled with your study ID number.

Other Procedures:

- If you are having health problems or there is a change in your health, the doctor may perform other tests or ask you to return to the clinic more often.
- Receive instruction on how to contact the site if you have any issues, especially after your final clinic visit

What are the possible risks, side effects, and discomforts of this research study?

RISKS FROM TRUVADA®

The drugs used in this study may have side effects, some of which are listed below. Please note that these lists do not include all the side effects seen with these drugs. These lists include the more serious or common side effects with a known or possible relationship. If you have questions concerning additional study drug side effects please ask the medical staff at your site.

Use of Combination Antiretroviral Drugs

Immune Reconstitution Syndrome: In some people with advanced HIV infection, symptoms from other infections or certain diseases may occur soon after starting combination anti-HIV treatment but can also occur later. Some of these symptoms may be life threatening. If you start having new symptoms, or notice that existing symptoms are getting worse after starting your antiretroviral therapy, tell your healthcare provider right away.

The use of potent antiretroviral drug combinations may be associated with an abnormal placement of body fat and wasting. Some of the body changes include:

- Increase in fat around the waist and stomach area
- Increase in fat on the back of the neck
- Thinning of the face, legs and arms
- Breast enlargement

Nucleoside Analogue

Lactic acidosis (elevated lactic acid levels in the blood) and severe hepatomegaly (enlarged liver) with steatosis (fatty liver) that may result in liver failure, other complications or death have been reported with the use of antiretroviral nucleoside analogues alone or in combination. The liver complications and death have been seen more often in women on these drug regimens. Some nonspecific symptoms that might indicate lactic acidosis include: unexplained weight loss, stomach discomfort, nausea, vomiting, fatigue, cramps, muscle pain, weakness, dizziness and shortness of breath.

Risks associated with Truvada®

The following side effects have been associated with the use of emtricitabine, one of the drugs in Truvada®

- Headache
- Dizziness
- Tiredness
- Inability to sleep, unusual dreams
- Loose or watery stools
- Upset stomach (nausea) or vomiting
- Abdominal pain
- Rash, itching, which sometimes can be a sign of an allergic reaction
- Skin darkening of the palms and/or soles
- Increased cough
- Runny nose
- Abnormal liver function tests, which could mean liver damage
- Increases in pancreatic enzyme (substances in the blood), which could mean a problem with the pancreas
- Increased triglycerides
- Increased creatine phosphokinase, which could mean muscle damage

The following side effects have been associated with the use of tenofovir, the other drug in Truvada®:

- Upset stomach, vomiting, gas, loose or watery stools
- Generalized weakness

- Dizziness
- Depression
- Headache
- Abdominal pain
- Worsening or new kidney damage or failure
- Inflammation or swelling and possible damage to the pancreas and liver
- Shortness of breath
- Rash
- Allergic reaction: symptoms may include fever, rash, upset stomach, vomiting, loose or watery stools, abdominal pain, achiness, shortness of breath, a general feeling of illness or a potentially serious swelling of the face, lips, and/or tongue
- Bone pain and bone changes such as thinning and softening which may increase the risk of breakage
- Muscle pain and muscle weakness

NOTE: If you become infected with Hepatitis B, you should be aware that your liver function tests may increase, and symptoms associated with hepatitis (an acute inflammation of the liver) may worsen if the drug tenofovir or emtricitabine is stopped.

RISKS FROM TENOFOVIR GEL

The gel can cause side effects. We do not yet know all the side effects of tenofovir gel on the rectum. Some men who used tenofovir gel in other studies complained of mild burning, irritation, or itching of the penis when exposed to gel. Others had mild rectal fullness, incontinence or diarrhea, gas, mild abdominal pain, or crampy pain in the rectum when using the gel rectally.

Risks from phlebotomy (blood tests)

- You may feel discomfort or pain when your blood is drawn.
- You may feel dizzy or faint.
- You may have a bruise, swelling, small clot, or infection where the needle goes in your arm or finger

Risks of finger and anoscope rectal exams

- You may feel discomfort or pressure when your rectum is examined
- You may experience some discomfort when the swab/sponge is inserted into the rectum, and occasionally minor rectal bleeding may occur

Risks from the applicator

- You may experience some discomfort from the applicator since the applicator has been designed for a vaginal rather than rectal use
- You may experience lacerations and bruising where the applicator is inserted

Other Possible Risks:

- You may become embarrassed, worried, or nervous answering personal questions about your sexual behavior, discussing ways to protect against HIV and other infections passed during sex, and your test results.
- You may become worried or nervous while waiting for your test results

- If you have HIV or other infections, knowing this could make you worried or nervous. A trained counselor will help you deal with any feelings or questions you have.
- If you become infected with HIV while using gel or tablets, it is possible that the medications in Truvada (tenofovir and emtricitabine) would not work against the HIV in your body. If this happened, it could limit your options for HIV treatment. It is for this reason that you must stop using gel or tablets if you become infected with HIV. Study doctors are available to discuss this with you. They can also do blood tests that will show which HIV medications might work best for you.
- Participants also could have problems in their partner relationships associated with use or attempted use of study gel.
- It is possible that your participation in this study could become known to others. This may cause you problems. You are encouraged to tell study staff about any issues you have as a result of taking part in this study.

We will make every effort to protect your privacy while you are having the study visits, exams, and tests. Reports via computer or text messages will be stored in computers that are password-protected and will not include personal information that could identify or link information to you; only your study ID number will be recorded. You will be shown how to erase the text message sessions from your mobile phone by study staff. When staff talk with you about how and when you used the study products they will audio record the discussion using a digital audio recorder. In addition, if you agree and are selected to participate in the interviews, these will be audio recorded. The audio files will be put into writing by the person interviewing you or by another person who does not know you and does not have your personal information. The audio recordings will be destroyed as soon as they have been put into writing, usually this is about three months after your interview. The person in charge at this site will make sure that these records have been destroyed. You should NOT identify anyone in the in-depth interviews and any names that might be mentioned on the recording will NOT be noted. Instead a generic description will be used in the transcript (i.e., if you refer to a friend’s name, “FRIEND1” will be noted).

What are possible benefits from taking part in this study?

There are no direct benefits for taking part in this study, but you or others may have future benefit from information learned in this study. You may also learn more about HIV and other diseases and ways to protect yourself from infection. It is important that you know, however, that you will not be paid any additional money (beyond the reimbursement described below for study participation) if the study product being studied is eventually licensed for use. You will have physical and rectal exams. If these tests show that you might have any health problems, you will be told about medical care and other services available to you. This will be available to you even if you do not enroll in this study. You will get counseling and free condoms. If you have infections passed through sex, other than HIV infection, you will be offered medicine to treat them or provided information for where you may receive treatment. This treatment or referral for treatment is available to you even if you do not enroll in this study. This study does not provide medication for treatment of HIV/AIDS. If you become infected with HIV, you will be told where you can go for medical care, counseling, and other services.

What if there is new information learned during this study?
We will tell you about new information from this or other studies that may affect your willingness to stay in this study.

A description of this clinical trial will be available on http://www.ClinicalTrials.gov. This website will not include information that can identify you. At most, the website will include a summary of the results. You can search this website at any time.

Is it possible that I may be taken out of the study without my consent?
A study doctor may need to remove you from the study early without your permission if:

- The study is cancelled by the US Food and Drug Administration (FDA), US NIH, the drug companies supporting this study, CONRAD and Gilead, the US Office for Human Research Protections (OHRP), the MTN, the local government or regulatory agency, or the Institutional Review Board (IRB)/ the Ethics Committee (EC). An IRB is a committee that watches over the safety and rights of research participants
- The Study Monitoring Committee (SMC) recommends that the study be stopped early (A SMC reviews the progress of the study and the kinds of effects that people report while they are participating in the study)
- You cannot come to appointments
- You are unable or unwilling to follow study instructions
- Other administrative reasons

The study doctor may ask you to stop using the study products but continue to come in for follow-up visits and procedures if:

- You are unwilling to comply with study procedures
- A study doctor decides that using the gel or tablets would be harmful to your health
- You become infected with HIV
- You become infected with Hepatitis B
- You require a treatment that may not be taken while using the study products

Will there be any payments if I take part in this research study?
[*Site to insert information about local reimbursement:*] You will receive [*Site to insert amount* xx] for your time, effort, and travel to and from the clinic at each scheduled visit. You will receive [Site to insert amount $xx] for any visits which occur in between your normally scheduled visits, these are called interim visits. For phone calls and/or text messages, you will receive [Site to insert amount $xx]. For the in-depth phone interviews, you will receive [Site to insert amount $xx].

What are the costs?
There is no cost to you for study-related visits, study products, physical exam, laboratory tests or other procedures. *[Site to include additional information as applicable according to site capacity]*

Are there any other studies if you cannot join this one?
There may be other studies going on at this study clinic or in the community for which you may be eligible. If you wish, we will tell you about other studies that we know about. There

may also be other places where you can go for HIV counseling and testing. We will tell you about those places if you wish. If you choose not to take part in this study, it will have no effect on the regular medical care that is available to you at this clinic or elsewhere.

Who will know about my participation in this research study?
Any information about you obtained from this research will be kept as private as possible. All records related to your involvement in this research study will be kept in a [site to insert]. Your identity on these records will be indicated by a number rather than by your name, and the information linking these numbers with your name will be kept separate from the research records.

Efforts will be made to keep your information confidential. However, it is not possible to guarantee confidentiality. Your personal information may be disclosed if required by law. Any publication of this study will not use your name or identify you personally. *[Sites to insert if applicable:]* The study staff may use your personal information to verify that you are not in any other research studies.

Your records may be reviewed by:
- Study monitors
- Study staff
- CONRAD, the organization that supplies the study gel
- Gilead, the organization that supplies the study tablets
- Representatives of the US Federal Government, including the US Food and Drug Administration (FDA), the US Office for Human Research Protections (OHRP), NIH, and/or contractors of the NIH
- [Insert applicable local authorities, e.g., Ministry of Health, medicine control authority]
- [Insert names of applicable IRBs/ECs]

[Site to include/amend the following:] [LOCAL/STATE/NATIONAL] regulations require study staff to report the names of people who test positive for HIV and other infections passed during sex to the [LOCAL HEALTH AUTHORITY]. Outreach workers from the [LOCAL HEALTH AUTHORITY] may then contact you about informing your partners, since they also should be tested. If you do not want to inform your partners yourself, the outreach workers will contact them, according to the confidentiality guidelines of the [LOCAL HEALTH AUTHORITY].

[US sites to include:]In addition to the efforts made by staff to keep your personal information confidential, a Certificate of Confidentiality has been obtained from the US Federal Government for this study. This Certificate protects study staff from being forced to tell people who are not connected with this study, such as the court system, about your participation or information you provide for study purposes. However, if the study staff learns of possible child, elder or dependent adult abuse and/or neglect or a risk of harm to you or others, they will be required to tell the proper authorities. This Certificate does not prevent you from releasing information about yourself or your participation in the study.

What if I am injured as a result of participating in this study?
[Sites to specify institutional policy:] If you are injured, the *[institution]* will give you immediate necessary treatment for your injuries. You *[will/will not]* have to pay for this

treatment. This institution or the United States National Institutes of Health does not have a program to provide money for injuries. You will be told where you can receive additional treatment for injuries if needed.

May I withdraw my consent for participation in this research study?
[Sites to specify institutional policy:] Taking part in this study is completely voluntary. You may choose not to take part in this study or leave this study at any time. If you choose to stop participation or to leave the study, you will not lose the benefits of this clinic, nor will the confidentiality of the care provided for you here be affected. You should feel free coming back to this facility even if you decide not to participate in this study. If you want the overall results of the study after the study is over, let the study staff members know.

What do I do if I have questions?

If you ever have any questions about the study, or if you have a research-related injury, you should contact [INSERT NAME OF THE INVESTIGATOR OR OTHER STUDY STAFF] at [INSERT TELEPHONE NUMBER AND/OR PHYSICAL ADDRESS].

If you have questions about your rights as a research participant, you should contact [INSERT NAME OR TITLE OF PERSON ON THE IRB OR OTHER ORGANIZATION APPROPRIATE FOR THE SITE] at [INSERT PHYSICAL ADDRESS AND TELEPHONE NUMBER].

If you have questions about whom to contact at the research site, you should contact [INSERT NAME OF THE INVESTIGATOR OR COMMUNITY EDUCATOR OR COMMUNITY ADVISORY BOARD (CAB) MEMBER] at [INSERT PHYSICAL ADDRESS AND TELEPHONE NUMBER].

Optional Study Activities:

The following procedures are optional study activities. You do not have to agree to participate in these activities to join this study.

Phone Interview

You are also being asked to participate in an optional study activity. The purpose of this activity is to gain further understanding of your experiences using the study products, such as your preferences and opinions about the study product, your experiences with using the gel, and any problems you may have had using the study products. If you choose to participate in this additional study activity, you will be compensated for your time and effort. [Site to insert amount $xx and how participants will be compensated, e.g., next scheduled visit, money will be loaded onto a visa card, etc.]

You may be asked to participate in an interview by phone with a trained staff member who does not work at this facility, to discuss your use of the study product and your feelings about the study product and trial participation. About 40 individuals will be selected for this interview across multiple sites. This interview will take place at or around Visit 4. The interview will be audio-recorded to make sure they are done the same way with everyone and so that there is a record of the discussion.

____________________ Initials & Date	Yes, if chosen, I agree to participate in the phone interview
____________________ Initials & Date	No, I do want to participate in the phone interview

Extra Samples Group

Approximately 36 participants across a few sites will provide rectal tissue (biopsies) and rectal fluid to help researchers better understand where the study drugs go and how they work against HIV in the laboratory.

[Sites not participating in the Rectal Tissue and Fluid Subset please insert the following language:]
This research site is not participating in the collection of these extra samples.

[Sites participating in the Rectal Tissue and Fluid Subset please insert the following language:]

You may choose to provide rectal tissue and rectal fluid, but you do not have to agree to these extra procedures to participate in MTN-017. About [site to insert] participants who agree to provide rectal tissue and fluid samples at this site will be asked to provide these samples. You will have an enema and an exam of your rectum (flexible sigmoidoscopy). For the enema, a hollow tube about the thickness of a pencil will be used to put some fluid into your rectum to flush it out. This may need to be repeated so that any stool that is there is removed. A flexible sigmoidoscopy is when a flexible, hollow tube is placed inside your rectum so that the study doctor can take samples of rectal tissue. Study clinicians will take approximately 20 small tissue samples about the size of a grain of rice, from your rectum. Also, rectal fluid will be collected using a sponge.

If you agree to participate in the Extra Sample Group, the following risks apply:

- You will have an enema before the flexible sigmoidoscopy. The main risk from having an enema is temporary discomfort.
- You may experience some mild discomfort and feel like you have a "bloated stomach" from the air from the flexible sigmoidoscope
- Even though the risk is low, you may experience infection, mild rectal irritation and may feel a sudden urge to have a bowel movement
- You may experience limited rectal bleeding (1 to 2 days after the procedure) related to the biopsies. We will test a small amount of your blood [xx mL] to ensure we can safely take these tissue samples.
- You may experience low blood pressure
- Even though the risk is very rare, there is a chance that you may have a hole or a tear in the intestine. This happens once out of every 1,000 procedures. If this were to happen, surgery to repair the tear may be necessary. It is important that you do not put anything in your rectum for 3 days after the biopsies, this includes having receptive anal intercourse, because you may be at higher risk for getting or spreading an infection until the biopsy sites have healed
- There is the risk of discomfort and a small risk of bleeding with the insertion of rectal sponges. You should not use drugs that increase your likelihood of bleeding if you participate in this Extra Samples group.

If you agree, approximately 15 biopsies and rectal fluid samples will be taken Enrollment. Approximately 20 biopsies and rectal fluid samples will be taken at each of the following visits: Visit 4, 7, 10. You must agree to abstain from receptive anal intercourse and inserting anything into your rectum for three days after the collection of these biopsies. In addition, you must refrain from taking drugs associated with increase bleeding for three days before and for three days after the collection of these samples. Study staff will either call you to check on your health or you will be asked to call-in to report any issues you may have. Study staff will provide you with information about what you can expect during these procedures and what behaviors you should avoid. [Insert site information regarding participant reimbursement, if applicable.]

____________ Yes, if chosen, I want to take part in the Extra Samples Group
Initials & Date

____________ No, I do not want to take part in the Extra Samples Group
Initials & Date

Storage and Future Testing of Specimens and Related Information

There might be a small amount of your biological specimens left over after we have done all of the study-related testing after your study visits. We would like to ask your permission to store these samples and related health data for use in future studies. If you agree, your samples will be stored safely and securely at facilities that are designed so that only approved researchers will have access to the samples. In general, specimens remain in country for storage and testing. However, if storage space becomes limited, specimens may be shipped to a central location for the study that may be outside the country. All efforts will be made to have testing performed locally. However, some specialty tests or equipment may only be available at laboratories where we are certain that the test can be performed accurately or validated. Because of this some specimens may be shipped to those specialty laboratories for testing and they may be outside of this country. Some employees of the facilities will need to have access to your samples to store them and keep track of where they are, but these people will not have information that directly identifies you.

You can still enroll in this study if you decide not to have these samples stored for future studies. If you do not want the samples stored, we will destroy the leftover specimens. Any future studies that may be done will also have to be approved by an IRB/EC. [Sites to specify institutional policy:] There is no time limit on how long your samples or health data will be stored or when these leftover specimens may be tested.

_______________ I agree to allow my biological specimens and health data
Initials & Date to be used in future research studies.

_______________ I do not agree to allow my biological specimens and Initials &
Initials & Date health data to be used in future research studies.

SIGNATURES- VOLUNTARY CONSENT

[Insert signature blocks as required by the local IRB/EC:] If you have read this consent form, or had it read and explained to you, and you understand the information, and you voluntarily agree to have the study, please sign your name or make your mark below.

______________________	___________________________	_______________
Participant Name (print)	Participant Signature/Mark	Date and Time

______________________	___________________________	_______________
Study Staff Conducting Consent Discussion (print)	Study Staff Signature	Date and Time

______________________	___________________________	_______________
Witness Name (print)	Witness Signature	Date and Time

References

1. McGowan I. Microbicides for HIV prevention: reality or hope? *Curr.Opin.Infect.Dis.* 2010; 23: 26-31.

2. Leynaert B, Downs AM, De V, I. Heterosexual transmission of human immunodeficiency virus: variability of infectivity throughout the course of infection. European Study Group on Heterosexual Transmission of HIV. *Am.J.Epidemiol.* 1998; 148: 88-96.

3. Vittinghoff E, Douglas J, Judson F, McKirnan D, MacQueen K, Buchbinder SP. Per-contact risk of human immunodeficiency virus transmission between male sexual partners. *Am.J.Epidemiol.* 1999; 150: 306-311.

4. Carlos JA, Bingham TA, Stueve A, Lauby J, Ayala G, Millett GA, Wheeler D. The role of peer support on condom use among Black and Latino MSM in three urban areas. *AIDS Educ.Prev.* 2010; 22: 430-444.

5. Gorbach PM, Manhart LE, Hess KL, Stoner BP, Martin DH, Holmes KK. Anal intercourse among young heterosexuals in three sexually transmitted disease clinics in the United States. *Sex Transm.Dis.* 2009; 36: 193-198.

6. Coly A, Gorbach PM. Microbicide acceptability research: recent findings and evolution across phases of product development. Curr Opin HIV AIDS 2008;3(5):581-586.

7. Carballo-Dieguez A, Stein Z, Saez H, Dolezal C, Nieves-Rosa L, Diaz F. Frequent use of lubricants for anal sex among men who have sex with men: the HIV prevention potential of a microbicidal gel. Am J Public Health 2000;90(7):1117-1121.

8. Chariyalertsak S, Kosachunhanan N, Saokhieo P et al. HIV incidence, risk factors, and motivation for biomedical intervention among gay, bisexual men, and transgender persons in Northern Thailand. PLoS One 2011;6(9):e24295.

9. Lansky A, Brooks JT, DiNenno E, Heffelfinger J, Hall HI, Mermin J. Epidemiology of HIV in the United States. J Acquir Immune Defic Syndr 2010;55 Suppl 2:S64-S68. 2012.

10. Sanchez J, Lama JR, Peinado J et al. High HIV and ulcerative sexually transmitted infection incidence estimates among men who have sex with men in Peru: awaiting for an effective preventive intervention. J Acquir Immune Defic Syndr 2009;51 Suppl 1:S47-S51.

11. van GF, Varangrat A, Wimonsate W et al. Trends in HIV Prevalence, Estimated HIV Incidence, and Risk Behavior Among Men Who Have Sex With Men in Bangkok, Thailand, 2003-2007. J Acquir Immune Defic Syndr 2009.

12. Kinsler JJ, Cunningham WE, Nurena CR et al. Using Conjoint Analysis to Measure the Acceptability of Rectal Microbicides Among Men Who Have Sex with Men in Four South American Cities. AIDS Behav 2011.

13. Gilead Sciences. Viread Investigator's Brochure. September 2006.

14. Gilead Sciences. Truvada® (emtricitabine and tenofovir disoproxil fumarate) tablets. Package Insert, July 2011.

15. Anton P, Cranston R, Carballo-Dieguez A, Kashuba A, Khanukhova E, Elliott J, Janocko LE, Cumberland W, Mauck C, McGowan I, RMP-02/MTN-006: A phase 1 placebo-controlled trial of rectally applied 1% vaginal TFV gel with comparison to oral TDF. Paper # 34. 18th Conference on Retroviruses and Opportunistic Infections. Boston. 2011.

16. Dezzutti C, Rohan LC, Lynam J, Wang L, Friend D. TFV reformulation results in improved product safety for rectal application. 18th Conference on Retroviruses and Opportunistic Infections.Boston. 2011.

17. CONRAD. Tenofovir Gel (GS-1278) Investigator's Brochure. Fourth Edition, November 12, 2010.

18. Gilead Sciences. Viread (tenofovir disoproxil fumarate) tablets. Package Insert. November 2008.

19. Gilead Sciences. Viread company core safety information. September 2006.

20. Rohan. Unpublished data on file. Rectal gel (TFV/GRFT) and RGVF of TFV gel: 28-day rectal dose toxicity study in male and female rabbits.

21. Gallant JE, DeJesus E, Arribas JR et al. Tenofovir DF, emtricitabine, and efavirenz vs. zidovudine, lamivudine, and efavirenz for HIV. N Engl J Med 2006;354:251-260.

22. Grant RM, Lama JR, Anderson PL et al. Preexposure chemoprophylaxis for HIV prevention in men who have sex with men. N Engl J Med 2010;363(27):2587-2599.

23. Baeten JM, Donnell D, Ndase P, Mugo NR, Campbell JD, Wangisi J, Tappero JW, Bukusi EA, Cohen CR, Katabira E, Ronald A, Tumwesigye E, Were E, Fife KH, Kiarie J, Farquhar C, John-Stewart G, Kakia A, Odoyo J, Mucunguzi A, Nakku-Joloba E, Twesigye R, Ngure K, Apaka C, Tamooh H, Gabona F, Mujugira A, Panteleeff D, Thomas KK, Kidoguchi L, Krows M, Revall J, Morrison S, Haugen H, Emmanuel-Ogier M, Ondrejcek L, Coombs RW, Frenkel L, Hendrix C, Bumpus NN, Bangsberg D, Haberer JE, Stevens WS, Lingappa JR, Celum C. Antiretroviral Prophylaxis for HIV Prevention in Heterosexual Men and Women. *New England Journal of Medicine* 2012.

24. NIAID Bulletin. The FEM-PrEP HIV Prevention Study and Its Implications for NIAD Research. (http://www.niaid.nih.gov/news/newsreleases/2011/Pages/FEMPrEP.aspx) Accessed 7 December 2011.

25. Schwartz JL, Poindexter A, Wheeless A, Mauck CK, Callahan MM. Safety evaluation of 1% tenofovir gel in healthy men. *Int.J.STD AIDS* 2009; 20: 384-386.

26. Abdool Karim Q, Abdool Karim SS, Frohlich JA, et al. Effectiveness and safety of tenofovir gel, an antiretroviral microbicide, for the prevention of HIV infection in women. Science 2010;329:1168-74.

27. Press Release: NIH Discontinues Tenofovir Vaginal Gel in 'VOICE' HIV Prevention Stuidy. http://www.nih.gov/news/health/nov2011/niaid-25.htm accessed January 4, 2012.

28. MTN-007, unpublished, McGowan, data on file.

29. McGowan I, Hoesley C, Andrew P, et al. MTN-007: A phase 1 randomized, double-blind,placebo-controlled rectal safety and acceptability study of tenofovir 1% gel. Paper #34LB presented at: CROI 2012; 5-8 March 2012; Seattle, WA.

30. Jackson A, Moyle G, Watson V, et al. Tenofovir (TFV), Emtricitabine (FTC) Intracellular (IC) and Plasma, and Efavirenz (EFV) Plasma Concentration Decay Following Drug Intake Cessation: Implications for HIV Treatment and Prevention. Abstract N-111 presented at: CROI 2012; 5-8 March 2012; Seattle, WA.

31. Chen J, Flexner CW, Liberman RG, Skipper PL, Louissaint NA, Tannenbaum SR, Hendrix CW, Fuchs EJ. Comparative plasma and intracellular pharmacokinetics of microdosing and standard dosing of zidovudine with accelerator mass spectrometry (AMS). 11th International Workshop on Clinical Pharmacology of HIV therapy. Sorrento, Italy, April, 2010 (Abstract LB-04).

32. Louissaint N, Cao Y, Tannenbaum S, Skipper P, Liberman R, Chen J, Nimmagadda S, Anderson J, Hendrix CW. Single dose 14C-Tenofovir Distribution into Blood, Colon, and Vagina in Healthy Volunteers. Keystone Symposium: Protection from HIV: Targeted Intervention Strategies. March 20 - 25, 2011. Whistler, British Columbia, Canada. (Poster 309).

33. Press Release: FDA approves first drug for reducing the risk of sexually acquired HIV infection. http://www.fda.gov/NewsEvents/Newsroom/PressAnnouncements /ucm312210.htm accessed 16 July 2012.

34. Abdool KQ, Abdool Karim SS, Frohlich JA, Grobler AC, Baxter C, Mansoor LE, Kharsany AB, Sibeko S, Mlisana KP, Omar Z, Gengiah TN, Maarschalk S, Arulappan N, Mlotshwa M, Morris L, Taylor D. Effectiveness and safety of tenofovir gel, an antiretroviral microbicide, for the prevention of HIV infection in women. *Science* 2010; 329: 1168-1174.

35. Morrow KM, Ruiz MS. Assessing microbicide acceptability: a comprehensive and integrated approach. AIDS Behav 2008 Mar;12(2):272-83.

36. Pool R, Montgomery CM, Morar NS, Mweemba O, Ssali, A ,Gafos M, et al. A mixed methods and triangulation model for increasing the accuracy of adherence and sexual behaviour data: The Microbicides Development Programme. Plos One 2010;5(7):e11600.

37. Tolley EE, Harrison PF, Goetghebeur E, Morrow K, Pool R, Taylor D, et al. Adherence and its measurement in phase 2/3 microbicide trials. AIDS Behav 2010 Oct;14(5):1124-36.
